# Loss of microRNA-15a/16-1 function promotes neuropathological and functional recovery in experimental traumatic brain injury

**DOI:** 10.1172/jci.insight.178650

**Published:** 2024-06-24

**Authors:** Chao Zhou, Shun Li, Na Qiu, Ping Sun, Milton H. Hamblin, C. Edward Dixon, Jun Chen, Ke-Jie Yin

**Affiliations:** 1 Department of Neurology, University of Pittsburgh School of Medicine, Pittsburgh, Pennsylvania, USA.; 2 Geriatric Research Education and Clinical Center, Veterans Affairs Pittsburgh Healthcare System, Pittsburgh, Pennsylvania, USA.; 3 Division of Biomedical Sciences, School of Medicine, University of California Riverside, Riverside, California, USA.; 4 Department of Neurosurgery, University of Pittsburgh School of Medicine, Pittsburgh, Pennsylvania, USA.

**Keywords:** Inflammation, Therapeutics, Behavior, Demyelinating disorders, Drug therapy

## Abstract

The diffuse axonal damage in white matter and neuronal loss, along with excessive neuroinflammation, hinder long-term functional recovery after traumatic brain injury (TBI). MicroRNAs (miRs) are small noncoding RNAs that negatively regulate protein-coding target genes in a posttranscriptional manner. Recent studies have shown that loss of function of the miR-15a/16-1 cluster reduced neurovascular damage and improved functional recovery in ischemic stroke and vascular dementia. However, the role of the miR-15a/16-1 cluster in neurotrauma is poorly explored. Here, we report that genetic deletion of the miR-15a/16-1 cluster facilitated the recovery of sensorimotor and cognitive functions, alleviated white matter/gray matter lesions, reduced cerebral glial cell activation, and inhibited infiltration of peripheral blood immune cells to brain parenchyma in a murine model of TBI when compared with WT controls. Moreover, intranasal delivery of the miR-15a/16-1 antagomir provided similar brain-protective effects conferred by genetic deletion of the miR-15a/16-1 cluster after experimental TBI, as evidenced by showing improved sensorimotor and cognitive outcomes, better white/gray matter integrity, and less inflammatory responses than the control antagomir–treated mice after brain trauma. miR-15a/16-1 genetic deficiency and miR-15a/16-1 antagomir also significantly suppressed inflammatory mediators in posttrauma brains. These results suggest miR-15a/16-1 as a potential therapeutic target for TBI.

## Abstract 2

miR-15a/16-1 inhibition alleviates neuroinflammation, preserves white and grey matter integrity, reduces brain tissue loss, and improves sensorimotor and cognitive recovery after experimental traumatic brain injury.

## Graphical abstract

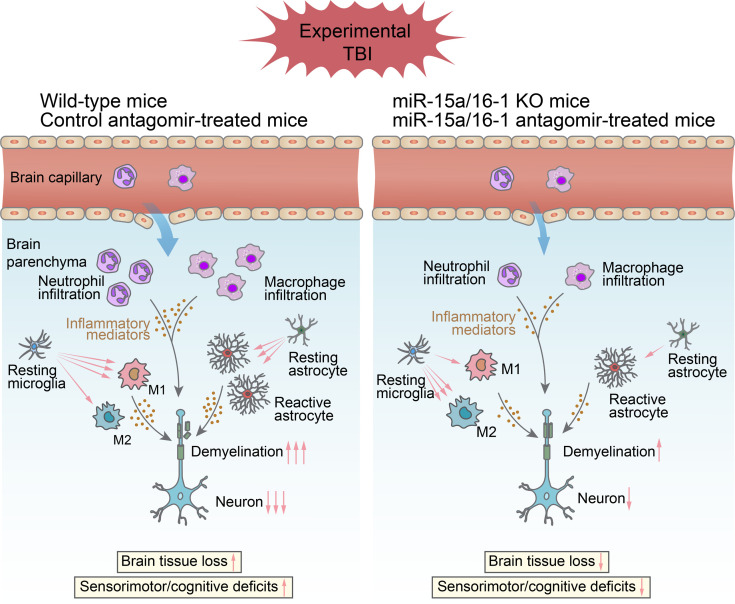

## Introduction

In recent years, traumatic brain injury (TBI) is gaining more attention as a cause of increased morbidity and mortality among young adults (1). Brain trauma is characterized by mechanical disruption of focal brain tissue (primary damage) and delayed diffuse brain damage (secondary damage) (2). Previous studies have demonstrated that TBI elicits gray matter injury (neuronal death) and white matter injury as well as severe inflammatory responses (3–5). The severity of primary and secondary damage in post-traumatic brains determines the progression of long-term neurological recovery (2). After brain trauma, the blood-brain barrier is immediately disrupted and peripheral blood immune cells, such as neutrophils and macrophages, infiltrate into brain parenchyma. Meanwhile, astrocytic activation and microglia polarization in pericontusional brains are also boosted. These peripheral and cerebral inflammatory cells trigger a severe inflammatory response that accelerates white/gray matter injuries after TBI. Therefore, it is imperative to identify mechanisms and develop effective therapeutic approaches that alleviate permanent brain damage and neurobehavioral dysfunctions after TBI.

MicroRNAs (miRs) are single-stranded noncoding RNAs that inhibit or induce degradation of targeted mRNAs by paring to 3′-untranslated regions (3′-UTR) of the regulated genes (6). Each miR can negatively regulate the expression of multiple target genes, and each gene is also regulated by numerous miRs. Studies have shown that several miRs were significantly elevated or suppressed in the brains and plasma of patients with TBI, as these altered miRs are potential biomarkers for the diagnosis and treatment of TBI (7). Dysfunctional cerebral miRs and their regulatory networks are critically involved in the pathogenesis of TBI. For example, miR-22 and miR-23-3p agomirs attenuated neuronal apoptosis, alleviated neuroinflammation, and improved neurobehavioral outcomes after TBI (8, 9), whereas the suppression of miR-193a and miR-155 reduced neuroinflammation and improved neurological functional recovery after TBI (10, 11). Additionally, miRs participated in the maintenance of white matter integrity after intracerebral hemorrhage, ischemic stroke, and other neurological diseases (12–14). However, their roles and underlying mechanisms in the regulation of long-term white matter injury and recovery after brain trauma are poorly understood.

We have previously demonstrated that pharmacological inhibition or genetic silencing of miR-15a/16-1 activity in mice significantly alleviated brain infarction and neuronal apoptosis and also improved short-term sensorimotor function after ischemic stroke (15). Moreover, endothelium-targeted deletion of the miR-15a/16-1 cluster mitigated the disruption and dysfunction of the blood-brain barrier, promoted postischemic angiogenesis, and improved long-term recovery of sensorimotor and cognitive functions in ischemic stroke by targeting claudin 5, vascular endothelial growth factor A (VEGFA), fibroblast growth factor 2 (FGF2), vascular endothelial growth factor receptor 2 (VEGFR2), and fibroblast growth factor receptor 1 (FGFR1) genes (16, 17). Most recently, we also found that genetic deletion of the miR-15a/16-1 cluster conferred resistance to white matter/gray matter injuries and neurobehavioral dysfunction in mice after vascular dementia by increasing cerebral expression of AKT3 and IL-10RA (18). However, whether suppression of miR-15a/16-1 activity can affect posttrauma white/gray matter damage, brain inflammatory responses, and neurologic deficits in TBI is unclear and warrants further investigation.

In this study, we presented a critical brain protective effect of the miR-15a/16-1 cluster on brain trauma by using experimental approaches including both genetic deletion and pharmacological inhibition. We expect that our findings will facilitate the development of the miR-15a/16-1 cluster as a molecular therapeutic target for the treatment of TBI.

## Results

### Genetic deficiency of the miR-15a/16-1 cluster promotes long-term recovery of sensorimotor and cognitive functions in mice after TBI.

**Figure 1:**
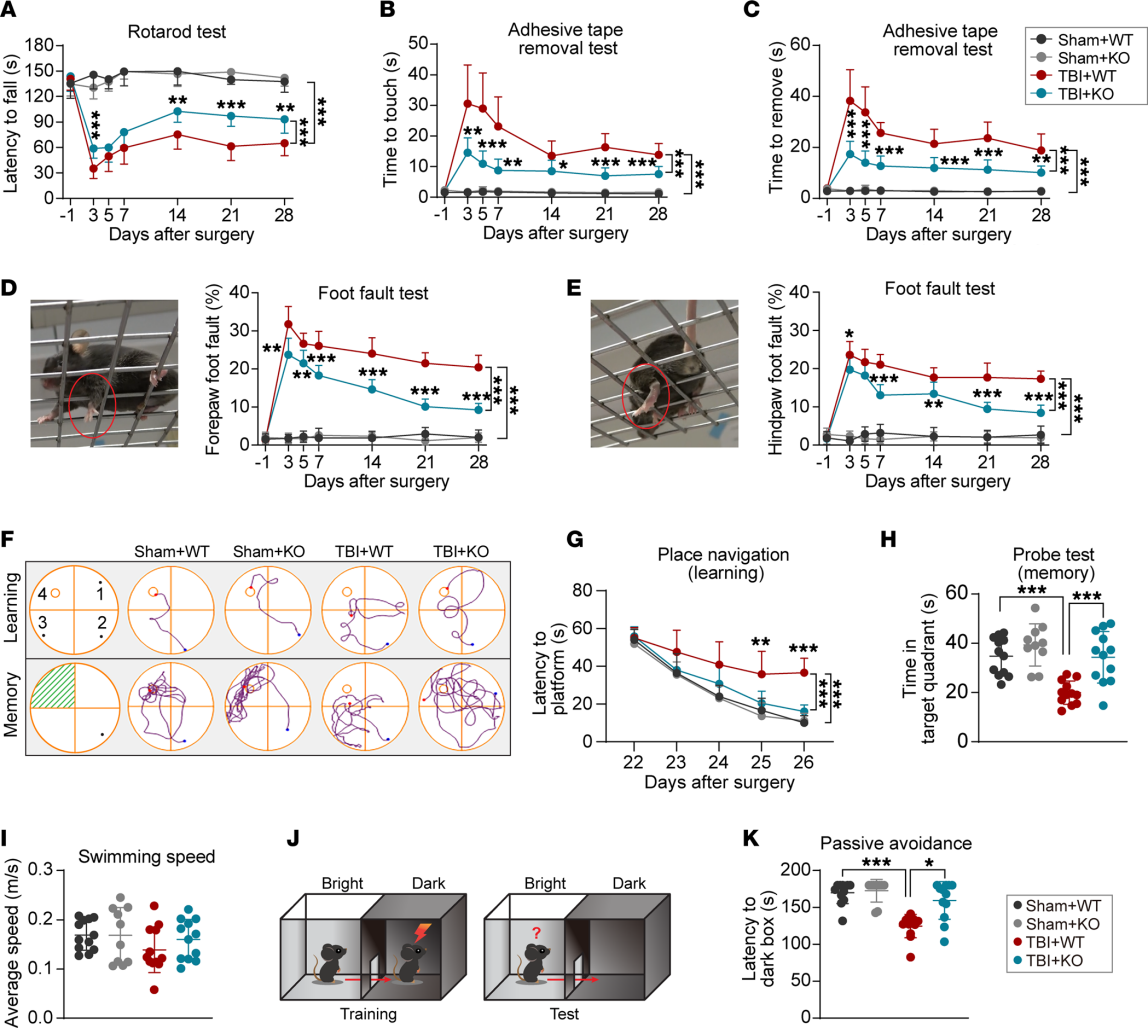
Genetic deletion of the miR-15a/16-1 cluster partially preserves sensorimotor and cognitive functions in mice after TBI. Experimental TBI was induced in miR-15a/16-1–KO and WT mice by unilateral controlled cortical impact, followed by a 30 days survival period. Long-term sensorimotor function was evaluated in TBI mice and sham controls at the indicated time points (–1, 3, 5, 7, 14, 21, and 28 days after operation). (**A**) Time to fall in the rotarod test. (**B** and **C**) The time to touch and time to remove the tape in the adhesive tape–removal test. (**D** and **E**) Representative forepaw/hindpaw foot-fault images and the forepaw/hindpaw foot-fault rate in the foot-fault test (red circle: foot fault). Long-term cognitive function was examined in TBI mice and sham controls by the Morris water maze (MWM) test (22–27 days) and the passive avoidance test (29–30 days). (**F**) Representative swimming track plots. (**G**) Latency to find platform in the learning phase. (**H**) Time spent in the target quadrant in the memory phase. (**I**) Average swimming speed in MWM test. (**J**) Graphic schedule of the passive avoidance test. (**K**) Latency to enter the dark box. Data are presented as mean ± SD, *n* = 10–12/group. Statistical analyses were performed by 1-way/2-way ANOVA and Tukey’s post hoc test. *P < 0.05, **P < 0.01, and ***P < 0.001 versus TBI + WT group.

The overall experimental design of this study is shown in Supplemental Figure 1 (supplemental material available online with this article; https://doi.org/10.1172/jci.insight.178650DS1). To explore the effect of miR-15a/16-1 genetic deletion on neurobehavioral function after TBI, miR-15a/16-1–KO and WT mice were subjected to a controlled cortical impact (CCI) (4.0 mm diameter; centered 0.5 mm anterior and 2.0 mm lateral to Bregma) or sham operation; then, rotarod, adhesive tape removal, and foot-fault tests were performed to evaluate the long-term sensorimotor function before and 3, 5, 7, 14, 21, and 28 days after the CCI operation. Compared with their corresponding sham controls, miR-15a/16-1–KO and WT mice exhibited severe sensorimotor deficits in the rotarod test (Figure 1A), adhesive tape–removal test (Figure 1, B and C), and foot-fault test (Figure 1, D and E) 3–28 days after TBI. In contrast, genetic deletion of the miR-15a/16-1 cluster significantly increased the staying time on the rod in the rotarod test (Figure 1A), decreased the time to touch or remove the tape in the adhesive tape–removal test (Figure 1, B and C), and reduced the foot-fault rate of the forepaw/hindpaw in the foot-fault test (Figure 1, D and E) 3–28 days after TBI when compared with WT mice, suggesting that miR-15a/16-1 genetic deficiency significantly improved posttrauma recovery of sensorimotor function. Of note, no significant differences were detected in sensorimotor function between miR-15a/16-1–KO and WT mice under sham conditions, as shown by similar performances in the rotarod test (Figure 1A), adhesive tape–removal test (Figure 1, B and C), and foot-fault test (Figure 1, D and E).

To investigate whether genetic deletion of the miR-15a/16-1 cluster alters cognitive function after TBI, we performed the Morris water maze (MWM) test and the passive avoidance test in miR-15a/16-1–KO and WT mice after TBI. In the MWM test, TBI mice (miR-15a/16-1 KO and WT) exhibited longer time to find the platform (Figure 1, F and G; learning) and a shorter time spent in the target quadrant (Figure 1, F and H; memory) than their corresponding sham controls, indicating declined posttrauma learning and memory capabilities. In comparison with WT controls, miR-15a/16-1–KO mice were able to spend less time to find the platform (Figure 1, F and G) and spend more time in the target quadrant (Figure 1, F and H) after TBI, suggesting significantly improved learning and memory abilities. Interestingly, there was no significant difference in swimming speed among all experimental groups (Figure 1I), indicating that posttrauma learning and memory outcomes in the MWM test were not affected by swimming speed. The passive avoidance test was performed as shown in Figure 1J. Sham-operated mice seldom crossed over the door to the dark environment after aversive footshock stimuli, whereas the TBI mice in both miR-15a/16-1–KO and WT groups presented a shorter waiting time before entering the dark environment versus their corresponding sham controls, indicating impaired memory ability after brain trauma (Figure 1K). Compared with WT controls, miR-15a/16-1–KO mice had a longer waiting time in a bright environment after TBI, indicating better memory ability (Figure 1K). No significant difference was detected in cognitive function between miR-15a/16-1–KO and WT mice under sham conditions, as evidenced by similar performance patterns in the MWM test (Figure 1, F–I) and passive avoidance test (Figure 1, J and K). Taken together, these results suggest that genetic deletion of the miR-15a/16-1 cluster significantly promotes the recovery of sensorimotor and cognitive functions in mice after CCI-induced brain trauma.

### Genetic deletion of the miR-15a/16-1 cluster reduces brain tissue loss and neuronal death in mice after TBI.

**Figure 2:**
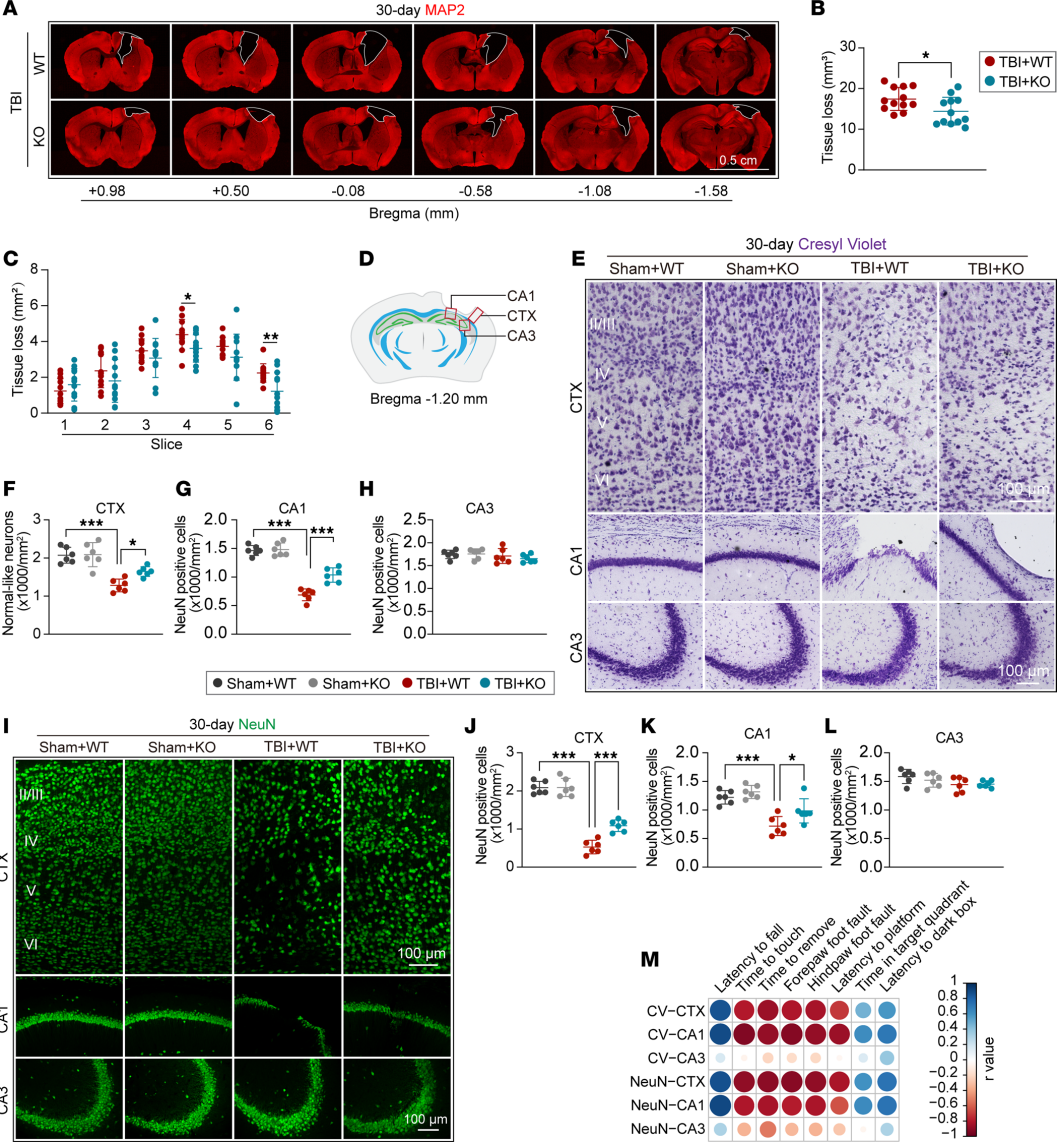
Genetic deficiency of the miR-15a/16–1 cluster reduces brain tissue loss and neuronal death in mice after TBI. MAP2 immunostaining was conducted in miR-15a/16-1–KO and WT mice 30 days after TBI to measure brain tissue loss. (**A**) Representative images of MAP2 immunostaining. Scale bar: 0.5 cm. (**B** and **C**) Quantitative analysis of volume or cross-sectional areas in brain tissue loss. Neuronal loss was examined by CV histological staining and NeuN immunofluorescence staining 30 days after TBI. (**D**) Coordinates and perilesional brain regions for CV and NeuN staining. (**E**) Representative images of CV staining in the pericontusional CTX, CA1, and CA3 regions. Scale bars: 100 μm. (**F**–**H**) Quantitative analysis of CV-stained cells in the pericontusional CTX, CA1, and CA3 regions. (**I**) Representative images of NeuN immunostaining in the pericontusional CTX, CA1, and CA3 regions. (**J**–**L**) Quantitative analysis of NeuN-immunopositive cells in the pericontusional CTX, CA1, and CA3 regions. Data are presented as mean ± SD, *n* = 6/group. Statistical analyses were performed by 1-way ANOVA and Tukey’s post hoc test. *P < 0.05, **P < 0.01, and ***P < 0.001 versus TBI + WT group. (**M**) Correlation analysis between sensorimotor or cognitive outcomes and CV-stained/NeuN^+^ neurons in the pericontusional CTX, CA1, and CA3 regions (*n* = 6/group, Pearson correlation analysis).

Next, we examined the volume of brain tissue loss in miR-15a/16-1–KO and WT mice 30 days after TBI by using MAP2 IF staining (Figure 2A). As demonstrated in Figure 2, A–C, genetic deletion of the miR-15a/16-1 cluster in mice protects against trauma-induced brain damage, showing smaller volume or cross-sectional areas of brain tissue loss (slice 4, 6) than WT controls 30 days after TBI. Neuronal death in cortical and hippocampal regions was further examined by Cresyl violet (CV) histological staining and NeuN (a neuronal nuclear marker) immunostaining in miR-15a/16-1–KO and WT mice 30 days after TBI. CV/NeuN staining images were taken from the cerebral cortex (CTX), hippocampal CA1 (CA1), and CA3 regions as shown in Figure 2D. Compared with sham controls, fewer CV-stained (Figure 2, E–G) and NeuN^+^ (Figure 2, I–K) neurons were detected in different layers of the perilesional CTX and CA1 areas of TBI mice. However, genetic deletion of the miR-15a/16-1 cluster in mice dramatically reduced posttrauma neuronal death, showing more CV-stained (Figure 2, E–G) and NeuN^+^ (Figure 2, I–K) neurons in the perilesional CTX and CA1 regions when compared with WT controls. Of note, there are no significant differences in neuronal density in the perilesional CTX, CA1, and CA3 regions in miR-15a/16-1–KO and WT mice under sham conditions. Also, no obvious neuronal loss in the CA3 region was detected in the experimental groups (Figure 2, H and L). Moreover, we conducted Pearson correlation analyses and showed that neuronal densities in the perilesional CTX and CA1 regions, but not in the CA3 region, were positively correlated with sensorimotor and cognitive functions in all experimental mice (Figure 2M and Supplemental Tables 2 and 3). These results suggest that the neuronal numbers in the perilesional CTX and CA1 regions were highly associated with posttrauma sensorimotor and cognitive dysfunctions and improvements. A higher neuronal density in the perilesional CTX and CA1 regions of miR-15a/16-1–KO mice may contribute to better sensorimotor and cognitive recoveries after TBI compared with WT controls.

### Genetic deletion of the miR-15a/16-1 cluster alleviates long-term white matter damage in mice after TBI.

**Figure 3:**
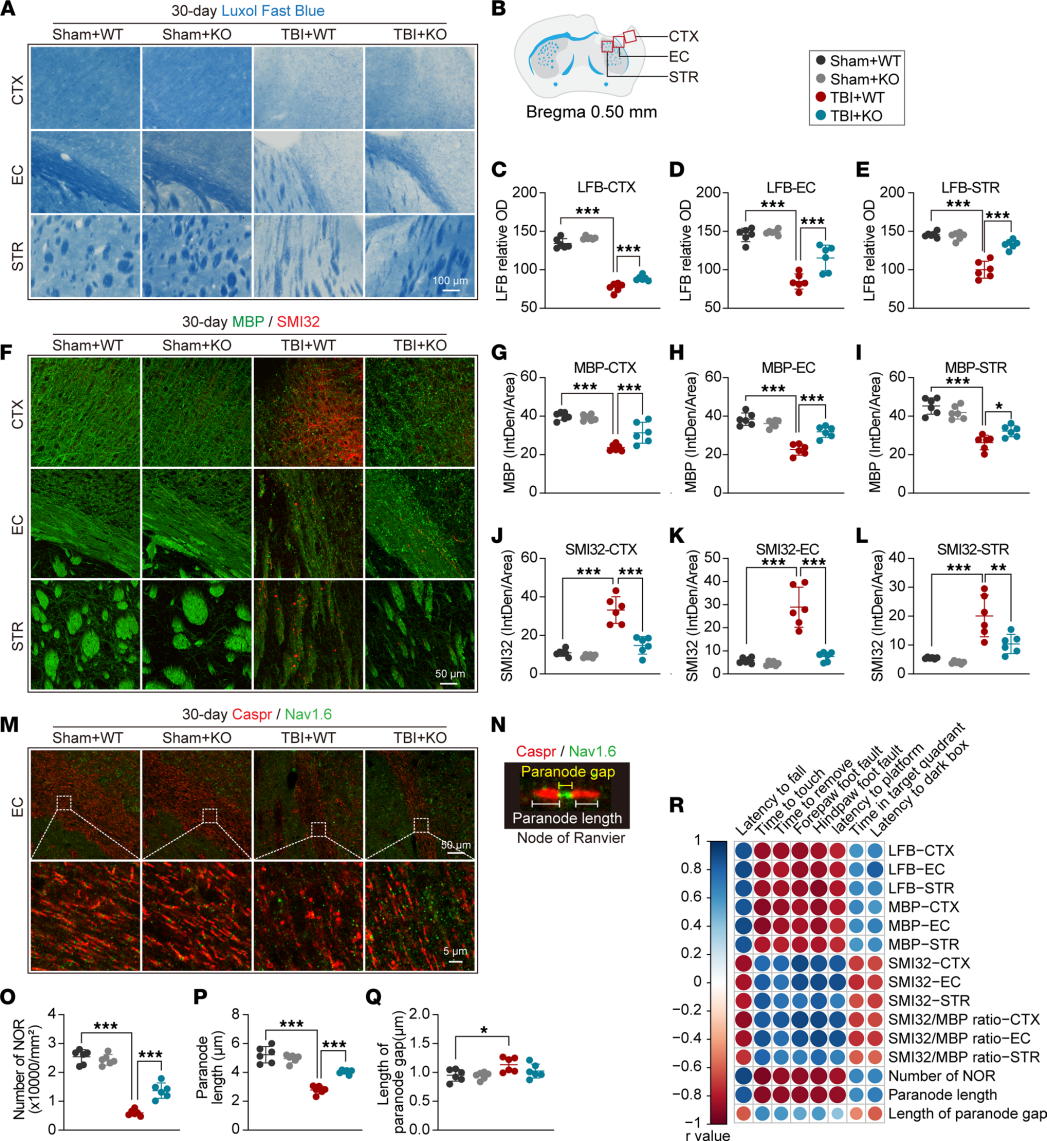
miR-15a/16–1 genetic deletion partially preserves long-term white matter integrity in mice after TBI. Luxol fast blue (LFB) histological staining and MBP (green)/SMI32 (red) double-immunofluorescence staining were used to evaluate white matter integrity in miR-15a/16-1–KO and WT mice 30 days after TBI. (**A**) Representative images of LFB staining in the pericontusional CTX, EC, and STR regions. Scale bars: 100 μm. (**B**) Coordinates and brain regions for LFB, MBP/SMI32, and Caspr/Nav1.6 staining. (**C**–**E**) Quantitative analysis of relative OD values from LFB staining in the pericontusional CTX, EC, and STR regions. (**F**) Representative images of MBP/SMI32 immunostaining in the pericontusional CTX, EC, and STR regions. Scale bar: 50 μm. (**G**–**I**) Quantitative analysis of MBP fluorescence intensity in the pericontusional CTX, EC, and STR regions. (**J**–**L**) Quantitative analysis of SMI32 fluorescence intensity in the pericontusional CTX, EC, and STR regions. (**M**) The node of Ranvier (NOR) was examined by Caspr (red)/Nav1.6 (green) double-immunofluorescence staining. Representative images of Caspr/Nav1.6 immunostaining in the pericontusional EC area. Scale bars: 50 μm (top), 5 μm (bottom). (**N**) A representative image showing the composition and normal structure of the NOR. (**O**–**P**) Quantitative analysis of the number of NOR (**O**), paranode length (**P**), and the length of the paranode gap (**Q**). Data are presented as mean ± SD, *n* = 6/group. Statistical analyses were performed by 1-way ANOVA and Tukey’s post hoc test. *P < 0.05, **P < 0.01, and ***P < 0.001 versus TBI + WT group. (**R**) Correlation analysis of sensorimotor or cognitive outcome and white matter integrity (*n* = 6/group, Pearson correlation analysis).

White matter lesions are considered a critical reason for cognitive impairment after TBI (4). Therefore, we examined white matter integrity by using Luxol fast blue (LFB) histological staining and myelin basic protein (MBP, myelin marker)/neurofilament heavy polypeptide (clone SMI32, injured axon marker) double-IF staining in the perilesional CTX, external capsule (EC), and striatum (STR) 30 days after TBI. As shown in Figure 3, the relative OD value of LFB (Figure 3, A–E) and the fluorescence intensity of MBP (Figure 3, F–I, and Supplemental Figure 2A) were significantly decreased in the perilesional CTX, EC, and STR 30 days after TBI in both miR-15a/16-1–KO and WT mice, indicating a significant loss of myelin in posttrauma brains compared with their corresponding controls. Additionally, the fluorescence intensity of SMI32 (Figure 3, J–L, and Supplemental Figure 2A) and the SMI32/MBP ratio (Supplemental Figure 2, B–D) were significantly increased in the perilesional CTX, EC, and STR 30 days after TBI, suggesting increased axonal damage in the posttrauma brains. Compared with WT controls, miR-15a/16-1–KO mice exhibited an increased OD value of LFB (Figure 3, A–E), increased MBP fluorescence intensity (Figure 3, F–I, and Supplemental Figure 2A), and decreased SMI32 fluorescence intensity and SMI32/MBP ratio (Figure 3, J–L, and Supplemental Figure 2, A–D) in the pericontusional CTX, EC, and STR, indicating that genetic deletion of the miR-15a/16-1 cluster effectively reduced white matter damage by alleviating the demyelination process and axonal damage in posttrauma brains. There were no significant differences in MBP, SMI32 fluorescence intensity, or SMI32/MBP ratio in the pericontusional CTX, EC, or STR in miR-15a/16-1–KO or WT mice under sham conditions. 

The normal structure of the node of Ranvier (NOR) is essential to maintain signaling conduction along myelinated axons (19). Therefore, contactin-associated protein (Caspr, a paranodal cell adhesion protein)/Nav1.6 (a nodal sodium channel protein) dual immunostaining was performed to examine the structure of the NOR (Figure 3N) in the perilesional EC 30 days after TBI. As shown in Figure 3, M–Q, the number of NORs (Figure 3, M and O) and paranode length (Figure 3P) were decreased, and the length of the paranode gap was increased in posttrauma brains compared with sham controls. Of importance, genetic deletion of the miR-15a/16-1 cluster in mice caused a significantly higher amount of NORs and longer paranode length in the perilesional EC region after TBI than WT controls, implying improved function of the NOR (Figure 3, O and P). However, genetic deletion of the miR-15a/16-1 cluster failed to alter the paranode gap in posttrauma brains (Figure 3Q).

As demonstrated in Figure 3R and Supplemental Tables 4 and 5, further correlation analyses showed a positive/negative correlation between the structural parameters of white matter (LFB, MBP, SMI32, SMI32/MBP ratio, and Caspr/Nav1.6) and neurobehavioral outcomes (sensorimotor and cognitive functions) in all experimental mice; this indicated that posttrauma white matter structural injury or loss in mouse brains was highly associated with sensorimotor and cognitive impairments. Improved white matter integrity may causatively contribute to better neurobehavioral function in miR-15a/16-1–KO mice after TBI compared with WT controls.

### Genetic deletion of the miR-15a/16-1 cluster decreases activation of astrocytes and microglia cells and decreases infiltration of blood-borne immune cells in mice after TBI.

**Figure 4:**
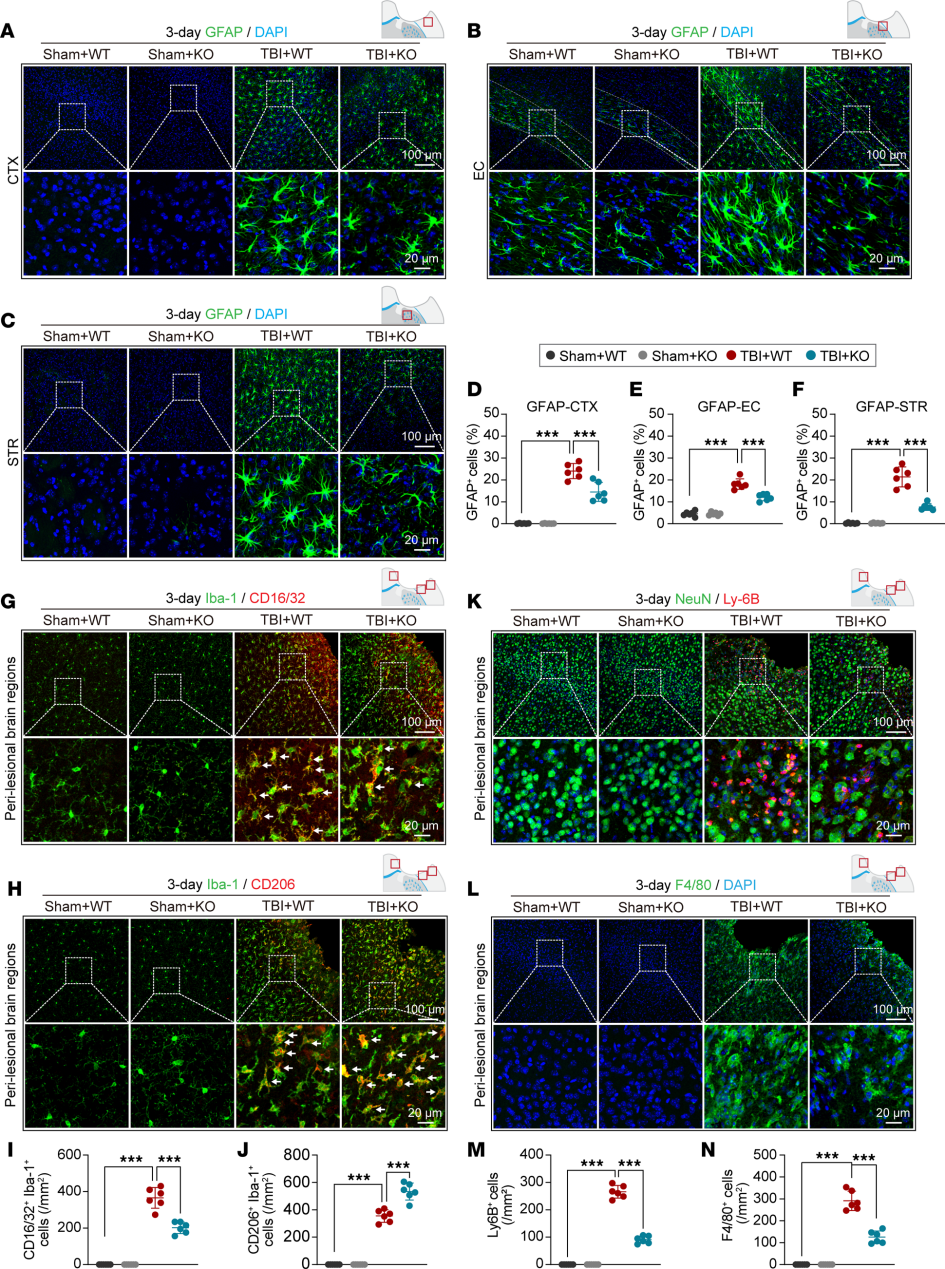
miR-15a/16–1 genetic deletion alleviates TBI-induced glial activation and infiltration of peripheral immune cells in mouse brains. Experimental TBI was induced in miR-15a/16-1–KO and WT mice, and then astrocytic activation and microglial polarization were examined in brain sections at 3 days after surgery. (**A**–**C**) Representative images of GFAP (green, an astrocyte marker)/DAPI (blue) immunofluorescence staining in the perilesional CTX, EC, and STR regions. (**D**–**F**) Quantitative analysis of GFAP^+^ cells in the perilesional CTX, EC, and STR areas. (**G** and **H**) Representative images of Iba-1 (green)/CD16/32 (red) and Iba-1 (green)/CD206 (red) double-immunofluorescence staining in the perilesional brain regions. (**I** and **J**) Quantitative analysis of Iba-1^+^CD16/32^+^ and Iba-1^+^CD206^+^ cells. The infiltration of peripheral neutrophils and macrophages was examined in perilesional brain regions. (**K** and **L**) Representative images of NeuN (green)/Ly-6B (red) and F4/80 (green)/DAPI (blue) immunostaining in the perilesional brain regions. (**M** and **N**) Quantitative analysis of Ly-6B^+^ neutrophils and F4/80^+^ macrophages in perilesional brain regions. Data are presented as mean ± SD, *n* = 6/group. Statistical analyses were performed by 1-way ANOVA and Tukey’s post hoc test. ***P < 0.001 versus TBI + WT group. Scale bars: 100 μm (top), 20 μm (bottom).

Since astrocytic activation is a symbolic event in posttrauma brains (2), we examined astrocytic activation in white/gray matter 3 days after TBI by using glial fibrillary acidic protein (GFAP) IF staining (Figure 4, A–C). We did not observe any significant differences in astrocytic activation in miR-15a/16-1–KO or WT mice under sham conditions. Compared with sham controls of 2 mouse strains, CCI induced considerable reactive astrocytes in the perilesional CTX, EC, and STR regions in miR-15a/16-1–KO and WT mice (Figure 4, A–F). However, significantly reduced GFAP^+^ astrocytes were detected in these brain regions of miR-15a/16-1–KO mice 3 days after TBI in comparison with WT controls, indicating that genetic deletion of the miR-15a/16-1 cluster dramatically reduces reactive astrocytes in posttrauma brains. Additionally, we examined the effect of miR-15a/16-1 on the long-term astrocytic activation after TBI. The results show that genetic deletion of miR-15a/16-1 reduced GFAP^+^ cells in the perilesional CTX, EC, and STR regions when compared with WT controls 30 days after TBI (Supplemental Figure 3, A–D).

Next, we examined the effect of miR-15a/16-1 genetic deletion on microglial/macrophage polarization by coimmunostaining Iba-1 with either CD16/32 (a proinflammatory microglial/macrophage marker) or CD206 (an inflammatory-resolving microglial/macrophage marker) in mouse brain sections at 3 days after TBI (Figure 4, G and H). Increased numbers of Iba-1^+^CD16/32^+^ and Iba-1^+^CD206^+^ microglia/macrophages were observed in the perilesional brain regions of miR-15a/16-1–KO and WT mice 3 days after TBI compared with their corresponding sham controls (Figure 4, H–J). Intriguingly, miR-15a/16-1–KO mice exhibited significantly decreased proinflammatory microglia/macrophages and increased inflammatory-resolving microglia/macrophages 3 days after TBI in comparison with WT controls (Figure 4, H–J). These data suggest that miR-15a/16-1 genetic deletion in mice promotes cerebral microglia/macrophage polarizing from a proinflammatory phenotype to an inflammatory-resolving phenotype in acute stage of brain trauma. The long-term effects of miR-15a/16-1 on microglia/macrophage polarization were also examined. We showed that genetic deletion of miR-15a/16-1 reduced the Iba-1^+^CD16/32^+^ cells in perilesional brain regions when compared with WT controls (Supplemental Figure 3, E and G). However, no significant Iba-1^+^CD206^+^ cells were detected between miR-15a/16-1–KO mice and WT controls either before or 30 days after TBI (Supplemental Figure 3, F and H).

Blood-brain barrier disruption and resultant infiltration of peripheral immune cells into the parenchyma occur in the acute phase of TBI and exacerbate neuroinflammatory reactions in posttrauma brains (20). To examine whether genetic deletion of the miR-15a/16-1 cluster affects peripheral infiltration of neutrophils and macrophages to posttrauma brains, we conducted immunostaining of NeuN/Ly-6B (a neutrophil marker) and F4/80 (a marker relatively specific to monocyte-derived macrophages) in mouse brain sections at 3 days after TBI. As shown in Figure 4, K–N, CCI induced abundant Ly-6B^+^ neutrophils and F4/80^+^ macrophages in the pericontusional brain regions of miR-15a/16-1–KO and WT mice in comparison with their corresponding sham controls. In contrast, genetic deletion of the miR-15a/16-1 cluster significantly reduced infiltration of peripheral neutrophils and macrophages after TBI compared with WT controls (Figure 4, K–N). Taken together, these results indicate that genetic deletion of the miR-15a/16-1 cluster in mice alleviates the inflammatory cell infiltrate in posttrauma brains.

### Intranasal delivery of the miR-15a/16-1 antagomir reduces TBI-induced sensorimotor and cognitive decline in mice.

The deficiency of miR15a/16-1 does not completely prevent most of the endpoints assessed as compared with uninjured miR-15a/16-1–KO shams. However, based on the above-described findings on genetic deletion of miR-15a/16-1–mediated intermediate brain protection in mice after TBI, we further explored the potential therapeutic role of pharmacological inhibition of cerebral miR-15a/16-1 in TBI mice by intranasal administration of the miR-15a/16-1 antagomir. As shown in Supplemental Figure 4, we examined the nose-to-brain delivery efficiency of the miR-15a/16-1 antagomir. A total of 1 nmol miR-15a/16-1 antagomir was intranasally delivered to C57BL/6J mice 2 hours after TBI. The relative expression levels of miR-15a and miR-16-1 were measured in mouse brains before TBI and 1, 2, 3, 5, and 7 days after TBI. Compared with nontreated mice, we found that a single 1 nmol dose of the miR-15a/16-1 antagomir was able to significantly reduce cerebral expression of miR-15a and miR-16-1 up to 7 days after administration under sham conditions (Supplemental Figure 4, A–C). Under TBI conditions, the expression of miR-15a in mouse brains was also significantly decreased up to 7 days after intranasal delivery (Supplemental Figure 4D), whereas cerebral expression of miR-16-1 was significantly reduced at 1–3 days and was modestly inhibited at 5–7 days after intranasal delivery (Supplemental Figure 4E). Afterward, C57BL/6J mice were intranasally delivered with 1 nmol of miR-15a/16-1 antagomir or its control antagomir at 2 hours, 5 days, 10 days, 15 days, 20 days, and 25 days after TBI to evaluate the therapeutic effect of the miR-15a/16-1 antagomir on TBI (Supplemental Figure 5).

**Figure 5:**
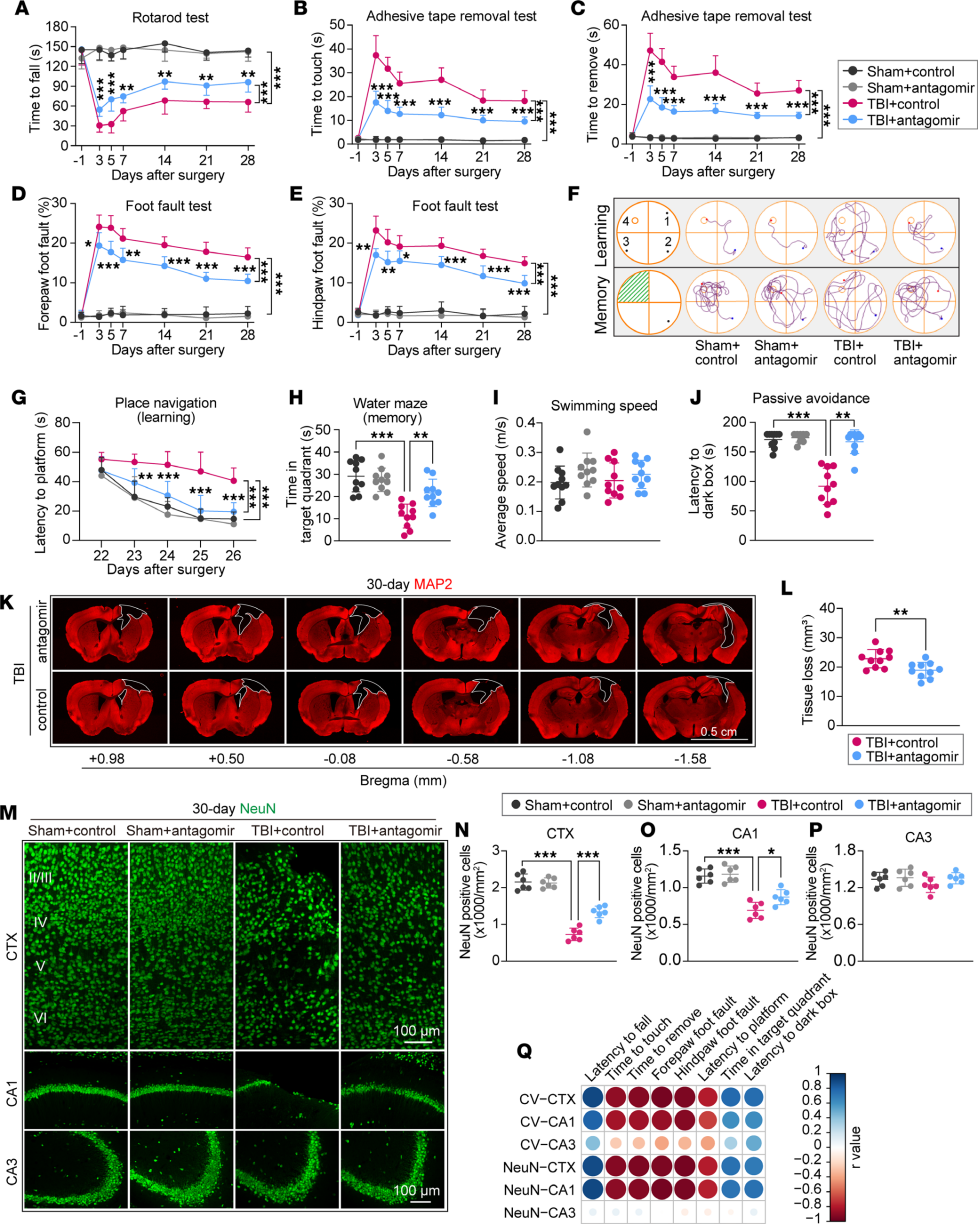
Long-term intranasal delivery of the miR-15a/16–1 antagomir promotes neurobehavioral recovery and reduces brain tissue loss and neuronal death in mice after TBI. C57BL/6J mice were subjected to experimental TBI and intranasal treatment of the miR-15a/16-1 antagomir or control antagomir at 2 hours, 5 days, 10 days, 15 days, 20 days, and 25 days after CCI surgery. Sensorimotor function was examined in experimental mice at the indicated time points (–1, 3, 5, 7, 14, 21, and 28 days after surgery). (**A**) Time to fall in the rotarod test. (**B** and **C**) The time to touch and time to remove the tape in the adhesive tape–removal test. (**D** and **E**) The forepaw foot-fault rate and hindpaw foot-fault rate in the foot-fault test. Cognitive function was evaluated by the MWM test (22–27 days) and the passive avoidance test (29–30 days). (**F**) Representative swimming track plots. (**G**) Latency to find platform in the learning phase. (**H**) Time spent in the target quadrant in the memory phase. (**I**) Average swimming speed in the MWM test. (**J**) Latency to enter the dark box. Data are presented as mean ± SD, *n* = 10/group. Statistical analyses were performed by 1-way/2-way ANOVA and Tukey’s post hoc test. MAP2 and NeuN immunostaining were performed to examine brain tissue/neuronal loss. (**K** and **L**) Representative images of MAP2 immunostaining and quantitative analysis of brain tissue loss volume (*n* = 10/group, unpaired *t* test). (**M**) Representative images of NeuN immunostaining in the pericontusional CTX, CA1, and CA3 regions. Scale bar: 100 μm. (**N**–**P**) Quantitative analysis of NeuN^+^ cells in the pericontusional CTX, CA1, and CA3 regions (*n* = 6/group, 1-way ANOVA & Tukey’s test). *P < 0.05 and ***P < 0.001 versus TBI + control antagomir group. (**Q**) Correlation analysis between sensorimotor or cognitive outcome and CV-stained or NeuN^+^ neurons in the pericontusional CTX, CA1, and CA3 regions (*n* = 6/group, Pearson correlation analysis).

A panel of sensorimotor neurobehavioral tests (rotarod test, adhesive tape–removal test, and foot-fault test) were conducted in miR-15a/16-1 antagomir–treated and control antagomir–treated mice before TBI and 3, 5, 7, 14, 21, and 28 days after TBI. Both control- or antagomir-treated mice exhibited remarkable sensorimotor deficits 3–28 days after TBI in comparison with their corresponding sham controls (Figure 5, A–E). However, miR-15a/16-1 antagomir–treated mice had a prolonged staying time to fall in the rotarod test (Figure 5A), reduced time to touch (Figure 5B) or remove the tape (Figure 5C) in the adhesive tape–removal test, and declined forepaw/hindpaw foot-fault rates in the foot-fault test (Figure 5, D and E) when compared with control-treated mice, indicating that nose-to-brain delivery of the miR-15a/16-1 antagomir can effectively rescue TBI-caused sensorimotor deficits in mice. We also examined long-term cognitive function in the miR-15a/16-1 antagomir–treated and its control-treated mice by using the MWM test and passive avoidance test. In the MWM test (Figure 5, F–I), control antagomir–treated mice had declined spatial learning and memory functions after TBI when compared with sham controls. However, mice treated with the miR-15a/16-1 antagomir showed significantly improved spatial learning and memory functions in comparison with the control antagomir–treated mice, as evidenced by showing a shorter time to find the platform in the learning phase and a longer time to explore in the target quadrant in the memory phase. There were no significant differences in swimming speed among all experimental groups (Figure 5I). In addition, miR-15a/16-1 antagomir–treated mice also showed an increased latency to the dark box after TBI compared with control antagomir–treated mice in the passive avoidance test (Figure 5J). Taken together, these results suggest that intranasal delivery of the miR-15a/16-1 antagomir promotes recovery of sensorimotor and cognitive functions in mice after TBI. Interestingly, under sham conditions, both miR-15a/16-1 antagomir–treated and control antagomir–treated mice exhibited similar performances in all neurobehavioral tests.

### Intranasal delivery of miR-15a/16-1 antagomir reduces brain tissue loss and neuronal death in mice after TBI.

We also examined brain tissue loss and neuronal death in miR-15a/16-1 antagomir or control antagomir–treated mice 30 days after TBI. MAP2 immunostaining (Figure 5K) showed that miR-15a/16-1 antagomir–treated mice exhibited smaller volume (Figure 5L) and cross-sectional areas (Supplemental Figure 6A; slice 1, 6) of brain tissue loss 30 days after TBI in comparison with control antagomir–treated mice, indicating that intranasal administration of the miR-15a/16-1 antagomir effectively reduced brain tissue loss in mice after TBI.

Neuronal density was further examined by CV staining (Supplemental Figure 6C) and NeuN immunostaining (Figure 5M) in miR-15a/16-1 antagomir and control antagomir–treated mice 30 days after TBI. Two groups of mice exhibited reduced CV-stained (Supplemental Figure 6, D and E) and NeuN^+^ (Figure 5, N and O) neurons in the perilesional CTX and CA1 compared with their corresponding sham controls. In contrast, miR-15a/16-1 antagomir treatment increased CV-stained and NeuN^+^ neurons in the pericontusional CTX and CA1 (Figure 5, N and O, and Supplemental Figure 6, D and E) and reduced neuronal death in mouse brains after TBI in comparison with control antagomir–treated mice. Mice with treatment of the miR-15a/16-1 antagomir showed no obvious changes in neuronal density of the CA3 region after TBI (Figure 5P and Supplemental Figure 6F). Both miR-15a/16-1 antagomir– and control antagomir–treated mice showed no changes in the neuronal density of CTX, CA1, and CA3 regions under sham conditions.

As demonstrated in Figure 5Q and Supplemental Tables 6 and 7, correlation analyses further show that neuronal densities in the pericontusional CTX and CA1 regions, but not in the CA3, were positively correlated with sensorimotor and cognitive functions, suggesting that neuronal numbers in the perilesional CTX and CA1 regions were highly associated with posttrauma sensorimotor and cognitive dysfunctions and improvements. Intranasal delivery of the miR-15a/16-1 antagomir reduced neuronal death in the pericontusional CTX and CA1 that may contribute to better sensorimotor and cognitive recovery after TBI.

### Intranasal delivery of the miR-15a/16-1 antagomir alleviates long-term white matter injury in mice after TBI.

**Figure 6:**
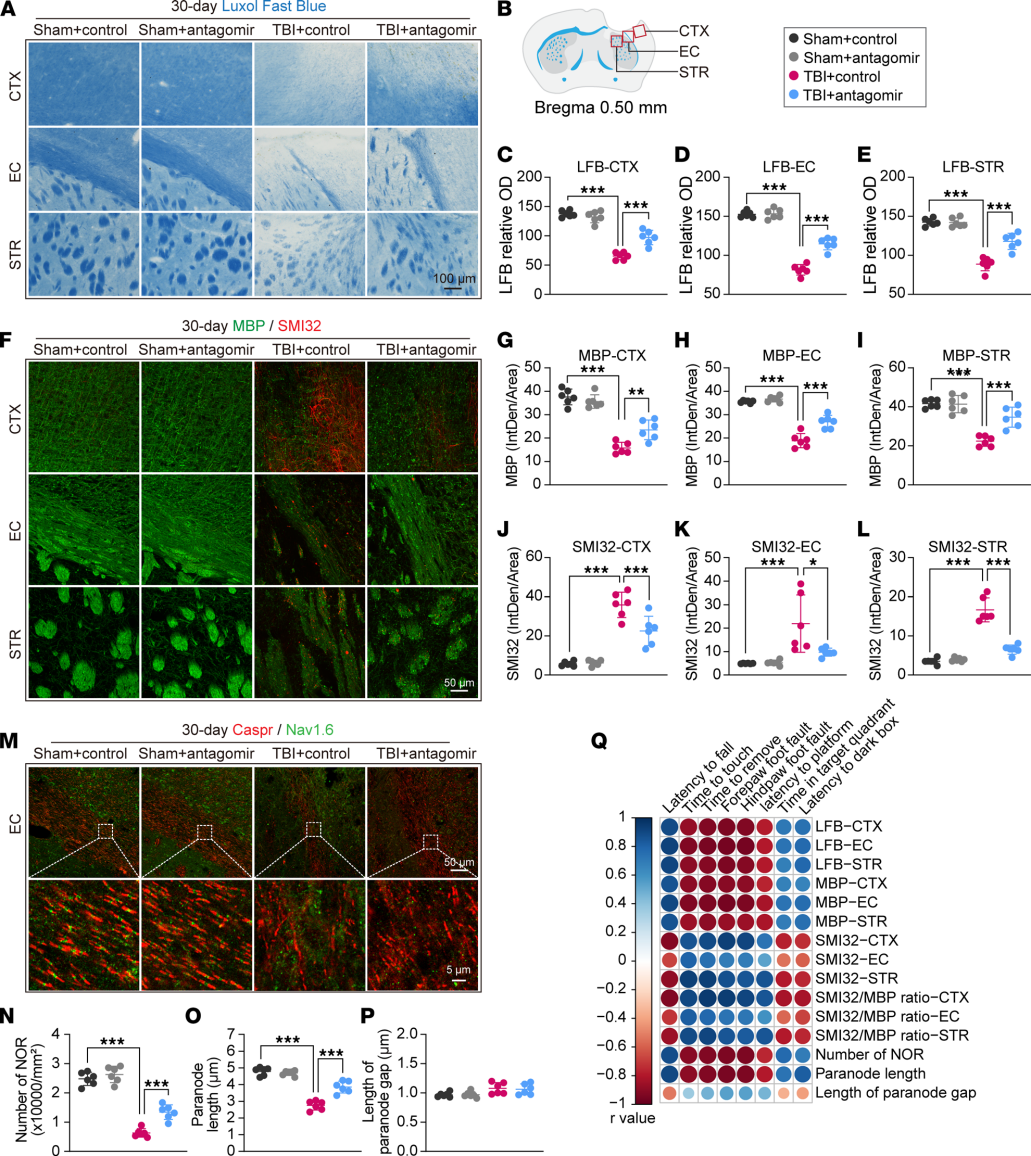
Long-term intranasal delivery of the miR-15a/16–1 antagomir partially preserves white matter integrity in mice after TBI. C57BL/6J mice were subjected to experimental TBI and intranasally treated with the miR-15a/16-1 antagomir or control antagomir at 2 hours, 5 days, 10 days, 15 days, 20 days, and 25 days after CCI surgery. White matter integrity was examined by LFB staining and MBP/SMI32 immunostaining 30 days after surgery. (**A**) Representative images of LFB staining in the perilesional CTX, EC, and STR regions. Scale bar: 100 μm. (**B**) Coordinates and brain regions for LFB, MBP/SMI32, and Caspr/Nav1.6 staining. (**C**–**E**) Quantitative analysis of relative OD values from LFB staining in the pericontusional CTX, EC, and STR regions. (**F**) Representative images of MBP/SMI32 immunostaining in the pericontusional CTX, EC, and STR regions. Scale bar: 50 μm. (**G**–**I**) Quantitative analysis of MBP fluorescence intensity in the pericontusional CTX, EC, and STR regions. (**J**–**L**) Quantitative analysis of SMI32 fluorescence intensity in the pericontusional CTX, EC, and STR regions. (**M**) The node of Ranvier (NOR) was examined by Caspr (red)/Nav1.6 (green) double-immunofluorescence staining. Representative images of Caspr/Nav1.6 immunostaining in the pericontusional EC area. Scale bars: 50 μm (top), 5 μm (bottom). (**N**–**P**) Quantitative analysis of the number of NOR (**N**), paranode length (**O**), and the length of the paranode gap (**P**). Data are presented as mean ± SD, *n* = 6/group. Statistical analyses were performed by 1-way ANOVA and Tukey’s post hoc test. *P < 0.05, **P < 0.01, and ***P < 0.001 versus TBI + control antagomir group. (**Q**) Correlation analysis of sensorimotor or cognitive outcome and white matter integrity (*n* = 6/group, Pearson correlation analysis).

Moreover, we examined white matter integrity in miR-15a/16-1 antagomir–treated mice 30 days after TBI. LFB histological staining and MBP/SMI32 dual immunostaining were conducted to examine white matter integrity in the pericontusional CTX, EC, and STR regions (Figure 6B). After TBI, both control- and antagomir-treated mice exhibited severe myelin loss when compared with their corresponding sham controls, showing a reduced LFB OD value and MBP fluorescence intensity in the pericontusional CTX, EC, and STR regions (Figure 6, A and C–I). However, intranasal delivery of the miR-15a/16-1 antagomir significantly reduced myelin loss after TBI in comparison with control antagomir–treated mice, showing both an enhanced LFB OD value and MBP fluorescence intensity in these brain regions (Figure 6, A and C–I). Additionally, control antagomir–treated mice exhibited an increased fluorescence intensity of SMI32 (Figure 6, F–L, and Supplemental Figure 7A) and the SMI32/MBP ratio (Supplemental Figure 7, B–D) in pericontusional CTX, EC, and STR 30 days after TBI, suggesting increased axonal damage in the posttrauma brains. In contrast, miR-15a/16-1 antagomir–treated mice showed decreased SMI32 fluorescence intensity and an SMI32/MBP ratio (Figure 6, J–L, and Supplemental Figure 7, A–D) in the pericontusional CTX, EC, and STR regions, suggesting that treatment with the miR-15a/16-1 antagomir alleviates axonal damage in mouse brains after TBI.

We further examined the structure of the NOR by coimmunostaining Caspr with Nav1.6 in miR-15a/16-1 antagomir–treated mice 30 days after TBI. Our results document that the number of NORs (Figure 6, M and N) and paranode length (Figure 6O) in the pericontusional EC region decreased in both control- and antagomir-treated TBI brains compared with their corresponding sham controls. However, miR-15a/16-1 antagomir–treated mice showed a significantly higher number of NORs and longer paranode length after TBI than control antagomir–treated mice, implying improved function for the NOR (Figure 3, N and O) in posttrauma brains after miR-15a/16-1 inhibition. No significant differences in the length of the paranode gap were found among all experimental groups (Figure 6P). 

As demonstrated in Figure 6Q and Supplemental Tables 8 and 9, further correlation analyses showed a positive/negative correlation between the structural parameters of white matter (LFB, MBP, SMI32, SMI32/MBP ratio, and Caspr/Nav1.6) and neurobehavioral outcomes (sensorimotor and cognitive functions) in all experimental mice. This indicates that greater white matter integrity in miR-15a/16-1 antagomir–treated mice may causatively contribute to the improved neurobehavioral function after TBI.

### Intranasal delivery of the miR-15a/16-1 antagomir reduces the activation of astrocytes and microglia cells and reduces infiltration of blood-borne immune cells in mice after TBI.

**Figure 7:**
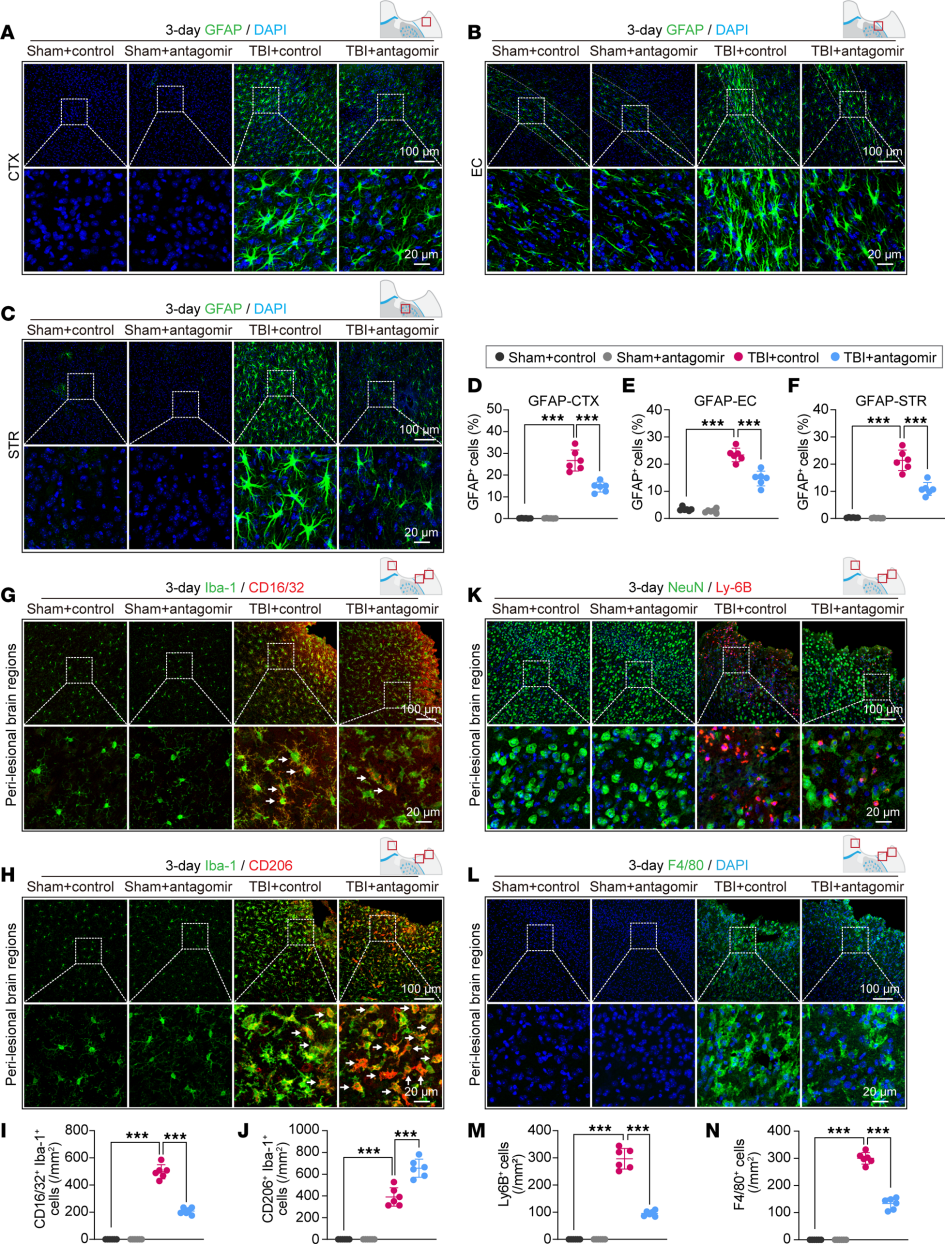
miR-15a/16–1 antagomir alleviates TBI-induced glial activation and infiltration of peripheral immune cells in mouse brains. C57BL/6J mice were subjected to experimental TBI and intranasally treated with the miR-15a/16-1 antagomir or control antagomir at 2 hours after surgery. Astrocytic activation and microglial polarization were examined in brain sections at 3 days after CCI surgery. (**A**–**C**) Representative images of GFAP (green, an astrocyte marker)/DAPI (blue) immunofluorescence staining in the perilesional CTX, EC, and STR regions. (**D**–**F**) Quantitative analysis of GFAP^+^ cells in the perilesional CTX, EC, and STR areas. (**G** and **H**) Representative images of Iba-1 (green)/CD16/32 (red) and Iba-1 (green)/CD206 (red) double-immunofluorescence staining in the perilesional brain regions. (**I** and **J**) Quantitative analysis of Iba-1^+^CD16/32^+^ and Iba-1^+^CD206^+^ cells. (**K** and **L**) The infiltration of peripheral neutrophils and macrophages was examined in perilesional brain regions. Representative images of NeuN (green)/Ly-6B (red) and F4/80 (green)/DAPI (blue) immunostaining in perilesional brain regions. (**M** and **N**) Quantitative analysis of Ly-6B^+^ neutrophils and F4/80^+^ macrophages in perilesional brain regions. Data are presented as mean ± SD, *n* = 6/group. Statistical analyses were performed by 1-way ANOVA and Tukey’s post hoc test. ***P < 0.001 versus TBI + control antagomir group. Scale bars: 100 μm (top), 20 μm (bottom).

We further tested whether the miR-15a/16-1 antagomir affects activation of astrocytes in mouse brains after TBI. As demonstrated in Figure 7, A–C, both control- and antagomir-treated mice showed a higher percentage of GFAP^+^ reactive astrocytes in the perilesional CTX, EC, and STR regions 3 days after TBI compared with their corresponding sham controls. However, treatment with the miR-15a/16-1 antagomir significantly reduced the percentage of reactive astrocytes in the perilesional CTX, EC, and STR regions following TBI (Figure 7, D–F).

Next, we examined the effect of the miR-15a/16-1 antagomir on microglia/macrophage polarization by coimmunostaining Iba-1 with either CD16/32 or CD206 in mouse brain sections at 3 days after TBI (Figure 7, G and H). Both control- and antagomir-treated mice showed increased numbers of Iba-1^+^CD16/32^+^ and Iba-1^+^CD206^+^ microglia/macrophages in the perilesional brain regions 3 days after TBI compared with sham controls of 2 treatment groups (Figure 7, I and J). However, miR-15a/16-1 antagomir–treated mice exhibited significantly decreased proinflammatory microglia/macrophages and increased inflammatory-resolving microglia/macrophages (Figure 7, I and J) after TBI. These data indicate that the miR-15a/16-1 antagomir promotes posttrauma cerebral microglia/macrophage polarization from a proinflammatory phenotype to an inflammatory-resolving phenotype.

In addition, we also conducted immunostaining of NeuN/Ly-6B and F4/80 in mouse brain sections from miR-15a/16-1 antagomir–treated mice 3 days after TBI. As demonstrated in Figure 7, K–N, both control- and antagomir-treated TBI mice showed an abundance of Ly-6B^+^ neutrophils and F4/80^+^ macrophages in the perilesional brain regions in comparison with sham controls of 2 treatment groups. In contrast, treatment with the miR-15a/16-1 antagomir in mice significantly reduced the infiltration of peripheral neutrophils and macrophages after TBI (Figure 7, M and N). Taken together, these results indicate that intranasal delivery of the miR-15a/16-1 antagomir alleviates inflammation-mediating cells in posttrauma brains.

### Genetic deletion and pharmacological inhibition of miR-15a/16-1 function reduce brain inflammatory mediators in mice after TBI.

**Figure 8:**
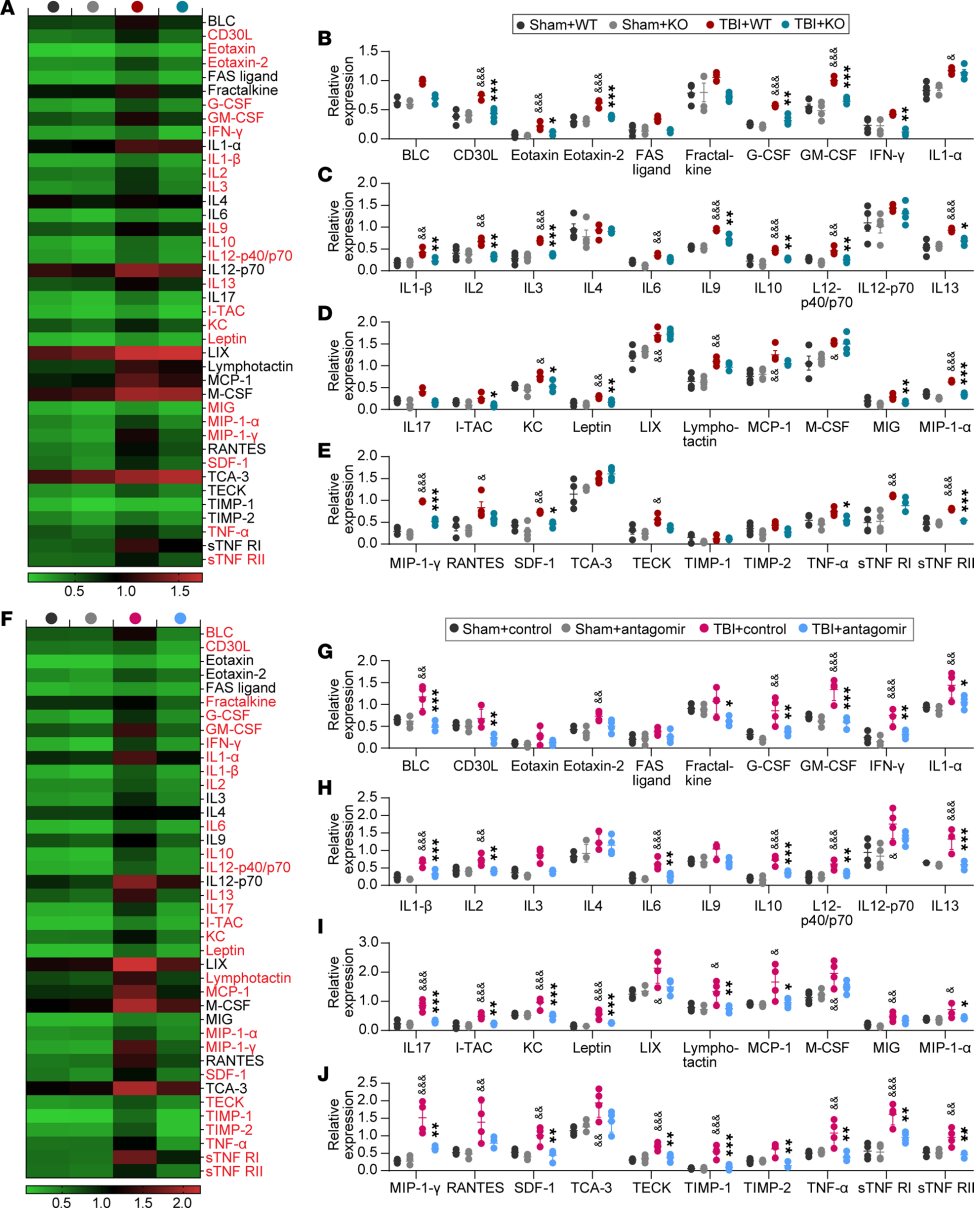
Genetic deletion and pharmacological inhibition of miR-15a/16–1 function reduces the inflammatory burden in posttrauma brains. (**A**–**E**) miR-15a/16-1–KO and WT mice were subjected to experimental TBI or sham operation. A panel of 40 inflammatory mediators was examined in mouse brains at 3 days after CCI surgery. (**A**) A heatmap showing the mean expression levels of 40 inflammatory mediators in posttrauma brains from experimental groups. (**B**–**E**) Quantitative analysis of 40 brain inflammatory mediators in experimental groups. Data are presented as mean ± SD, *n* = 4/group. Statistical analyses were performed by 1-way ANOVA and Tukey’s test or Kruskal-Wallis test & Dunn’s test. ^&,^*P < 0.05, ^&&,^**P < 0.01, and ^&&&,^***P < 0.001, where & represents TBI + WT versus Sham + WT group and * represents TBI + KO versus TBI + WT group. (**F**–**J**)C57BL/6J mice were subjected to experimental TBI and intranasally treated with the miR-15a/16–1 antagomir or control antagomir at 2 hours after surgery. A panel of 40 inflammatory mediators was measured in mouse brains at 3 days after CCI surgery. (**F**) A heatmap showing the mean expression levels of 40 inflammatory mediators in posttrauma brains from experimental groups. (**G**–**J**) Quantitative analysis of 40 brain inflammatory mediators in experimental groups. Data are presented as mean ± SD, *n* = 4/group. Statistical analyses were performed by 1-way ANOVA and Tukey’s test or Kruskal-Wallis test and Dunn’s test. ^&,^*P < 0.05, ^&&,^**P < 0.01, and ^&&&,^***P < 0.001 where & represents TBI + control antagomir versus Sham + control antagomir group and * represents TBI + miR-15a/16-1 antagomir versus TBI + control antagomir group.

To further investigate the underlying mechanism of brain protective effects conferred by either genetic deletion or pharmacological inhibition of miR-15a/16-1 function after TBI, a panel of 40 inflammatory mediators was examined by using a commercial inflammatory antibody array kit (Supplemental Figure 8). Compared with sham controls, we found that 27 inflammatory mediators were significantly elevated in the perilesional brain regions 3 days after TBI (Figure 8, A–E). Genetic deletion of the miR-15a/16-1 cluster significantly reduced the protein levels of 22 of these cerebral inflammatory mediators in mice 3 days after TBI compared with WT controls (Figure 8, B–E). On the other hand, altered cerebral inflammatory mediators were also found in miR-15a/16-1 antagomir–treated mice 3 days after TBI. We uncovered 32 significantly elevated cerebral inflammatory mediators in mice 3 days after TBI compared with sham controls, and treatment with the miR-15a/16-1 antagomir dramatically reduced 28 of the inflammatory mediators (Figure 8, F–J). Among these cerebral inflammatory mediators, CD30L, granulocyte CSF (G-CSF), granulocyte/macrophage CSF (GM-CSF), IFN-γ, IL-1β, IL-2, IL-10, IL-12-p40/p70, IL-13, I-TAC, KC, Leptin, MIP-1α, MIP-1γ, Stromal cell–derived factor-1 (SDF-1), TNF-α, and soluble tumor necrosis factor receptor (sTNF RII) are suppressed by both genetic deletion and antagomir inhibition of miR-15a/16-1 function in mice after TBI.

Taken together, although TBI-induced injuries persist in both miR-15a/16-1–KO and antagomir-treated mice, the in vivo loss of miR-15a/16-1 function partially reduced the inflammatory burden in TBI brains via suppressing various cerebral inflammatory mediators. These downregulated cerebral inflammatory mediators might be the downstream targets of the miR-15a/16-1 cluster and may contribute to the improved histological and functional outcomes found in either miR-15a/16-1–KO mice or miR-15a/16-1 antagomir–treated mice after TBI.

## Discussion

In this study, we revealed that genetic deletion or pharmacological inhibition of miR-15a/16-1 activity effectively reduces brain white matter and gray matter damage and improves long-term functional recovery in mice after TBI.

Patients with TBI suffer from severe posttrauma neurobehavioral impairment such as sensory/motor deficits, irritability, and amnesia (1, 21, 22). For patients with over 3 months of posttraumatic amnesia, functional recovery is highly improbable (23). Thus, early rehabilitative treatments to promote sensorimotor and cognitive recovery are critical for patients with TBI (21). Regulatory miRs play a critical role in the functional recovery of TBI (7). For example, intracerebroventricular (ICV) administration of miR-124-3p and miR193a antagomirs, or delivery of the miR-203 antagomir by oral gavage, can significantly improve sensorimotor and cognitive functions in rodents after TBI (10, 24, 25). We have previously demonstrated the brain-protective effects of miR-15a/16-1 gene deficiency in experimental vascular cognitive impairment and dementia (VCID) or ischemia stroke (15, 16, 18). In the current study, we reported that both nose-to-brain inhibition and miR-15a/16-1 genetic deletion promoted long-term sensorimotor and cognitive recovery in mice after TBI.

Intranasal delivery is a noninvasive delivery method and may bypass the blood-brain barrier, which is considered more effective and specific to the brain than other delivery approaches (26). The present study utilized the intranasal delivery of the miR-15a/16-1 antagomir. It showed promising translational value for future development of miR-based functional rehabilitative treatments in brain trauma.

Cerebral cortical and hippocampal tissue loss and atrophy are common pathological characteristics in patients with TBI that contribute to posttrauma learning and memory deficits (3, 27–29). Previous studies have shown conspicuous brain tissue loss and perilesional neuronal death in mouse brains after CCI-induced TBI (30–32), which can be regulated by miRs (33). For instance, it was reported that inhibition of miR-203 and miR-193a protected against TBI-induced neuronal apoptosis, brain tissue loss, and neuronal degeneration in mice (10). Upregulation of cerebral Let-7i, miRNA-9-5p, and miR-23b were able to reduce neuronal apoptosis, increase cortical neuronal density, or lessen neuronal autophagy in TBI brains (25, 34–36). Consistent with these previous studies showing neuroprotective effects by various manipulations of cerebral miRs in posttrauma brains, our current study elucidated that either genetic deletion of the miR-15a/16-1 cluster or antagomir inhibition of miR-15a/16-1 activity mitigated brain tissue loss and restored neuronal density in the pericontusional CTX and CA1 regions in mice after TBI. Furthermore, miR-15a/16-1 genetic deficiency or antagomir knockdown–derived neuroprotection in the pericontusional CTX and CA1 regions were positively correlated with improvement of posttrauma sensorimotor and cognitive functions in mice.

White matter injury is one of the major characteristic features in posttrauma brains and mainly contributes to cognitive impairment and decline in TBI (4, 37). Recently, several animal studies were conducted to examine posttrauma white matter injury and recovery in rodent experimental TBI models (30–32, 38, 39). miRs are also involved in the regulation of white matter integrity and oligodendrocyte development in many neurological diseases (12, 40–42). Although miRNAs are abundantly expressed in brain white matter, the essential regulatory role of miRs in brain trauma-induced white matter injury and recovery are poorly investigated (43). We previously demonstrated that miR-15a/16-1 gene deficiency in mice improved long-term myelin integrity and alleviated axonal damage in mice after VCID (18). Extending these findings to a different animal disease model, we report here that both miR-15a/16-1 genetic deletion and antagomir inhibition significantly promoted myelin integrity and reduced diffuse axonal damage in mice after TBI. On the other hand, it was reported that the normal structure of Ranvier was disrupted in experimental TBI and presented as asymmetrical paranode pairs and abnormal heminodes (44). In this study, we found that genetic deletion or intranasal delivery of the miR-15a/16-1 antagomir increased the number of NORs and maintained the paranode length in the pericontusional EC after TBI when compared with controls. Further analysis demonstrated that white matter structural improvements described above were positively correlated or contributed to the recovery of posttrauma sensorimotor and cognitive functions in miR-15a/16-1–KO or antagomir-treated mice.

The prevalent neuroinflammatory events in posttrauma brains are composed of complex, multifactorial processes that contribute to widespread neuronal death and chronic cell degeneration (45–48). Previous studies demonstrated a persistently high GFAP level in the serum of patients with TBI that was considered as a TBI outcome predictor (49, 50). A continuous existence of reactive astrocytes was also detected in mouse brains after CCI-induced TBI (47). On the other hand, it is becoming clear that activated microglia/macrophages may exhibit dual regulatory roles depending on the polarization toward different phenotypes in the pathogenesis of TBI. Recently, miRs were shown to mediate the cerebral inflammatory response in mice after TBI (33). For instance, miR-27a, miR-203, miR-146a, miR-125b, and miR-199b were shown to suppress microglial NF-κB signaling pathways after TBI (51). Treatment of the miR-Let-7i agomir reduced astrocytic and microglial scar formation after TBI (36). Up to now, the role of the miR-15a/16-1 cluster in regulating microglia/macrophage polarization in posttrauma brains has been unclear. The present study shows that genetic deletion of miR-15a/16-1 or intranasal administration of the miR-15a/16-1 antagomir suppressed reactive astrocytes and proinflammatory microglia in the pericontusional white matter and gray matter in mice 3 days and 30 days after TBI. Loss of function of miR-15a/16-1 also promoted the polarization of inflammatory-resolving microglia in mice 3 days after TBI.

In addition to microglial and astrocytic activation, the infiltration of peripheral blood immune cells, such as neutrophils and macrophages, into the brain parenchyma and subsequent release of proinflammatory cytokines are critical pathological events that exacerbate inflammatory responses in posttrauma brains (47, 52, 53). Several studies have demonstrated that miR-21 and miR-9-5p agomirs partially preserved the expression of tight junction proteins, reduced BBB permeability, and alleviated neuroinflammation in mice after TBI. Until now, less attention has been paid to investigate the role of miRs, including miR-15a/16-1, in regulating peripheral immune cell infiltration in posttrauma brains. The present study showed that genetic deletion of the miR-15a/16-1 cluster and intranasal delivery of miR-15a/16-1 antagomir partially prevented infiltration of peripheral neutrophils and macrophages to the cerebral parenchyma in TBI mice.

Glial cell activation and the infiltration of peripheral neutrophils and macrophages trigger the release of various inflammatory cytokines into posttrauma brains (20). These released inflammatory cytokines interact with each other and aggravate neuroinflammation in TBI brains (5). Previous studies show that IL-12 is required for the induction of IFN-γ production (54), and the expression of I-TAC (CXCL11) is strongly induced by IFN-γ (55). IL-1β, TNF-α/TNFRI, and CD30L signals are considered intense stimulators of the NF-κB proinflammatory pathway (56, 57). In addition, leptin-induced neutrophil migration is dependent on CXCL1/KC signaling cascades (58). SDF-1, G-CSF, and GM-CSF are also considered as triggers of leukocytes recruitment (59, 60). Moreover, IL-1β strongly stimulated the expression of macrophage inflammatory proteins (MIP), including MIP-1α and MIP-1β (61). In the present study, we documented that genetic deletion of miR-15a/16-1 and the miR-15a/16-1 antagomir significantly suppressed many critical inflammatory mediators mentioned above in posttrauma brains. Among these inflammatory mediators, IL-10 and IL-13 are antiinflammatory cytokines that protect against TBI-induced neuroinflammation (62, 63). After TBI, we observed a decline of IL-10 and IL-13 in the brains of miR-15a/16-1–KO mice or miR-15a/16-1 antagomir–treated mice, which might be an indicator of a reduced parenchymal neuroinflammatory response after TBI. However, the most suppressed inflammatory mediators are proinflammatory cytokines and, thus, loss of miR-15a/16-1 function or delivery of the miR-15a/16-1 antagomir significantly reduced the inflammatory burden in TBI brains.

In conclusion, we reported that genetic deletion of the miR-15a/16-1 cluster or intranasal treatment of the miR-15a/16-1 antagomir alleviated innate/infiltrated inflammatory cell responses, partially preserved white matter integrity, reduced neuronal loss and brain atrophy, and promoted the recovery of sensorimotor and cognitive functions in mice after TBI. The findings from our study will advance our knowledge regarding the regulatory role of miRs in TBI and may help in developing miR-based drugs for the treatment of brain trauma.

## Methods

### Sex as a biological variable.

In the current study, only male mice were used because male mice exhibited less variability in phenotype.

### Animals and experimental design.

Male homozygous miR-15a/16-1–KO mice were provided by Riccardo Dalla-Favera (University of Columbia, New York, New York, USA) (64). Male littermate WT mice were used for controls (8–12 weeks, 23–30 g). Both miR-15a/16-1–KO and WT mice were randomly assigned to either the sham or TBI group by a lottery box. All experimental mice survived, and no mice were excluded from this study. All outcomes were performed by independent investigators blinded to procedures and mouse genotypes.

### Murine model of TBI.

TBI was induced in miR-15a/16-1–KO and WT mice by using a CCI device (Precision Systems and Instrumentation) as described previously (31). Briefly, mice were anesthetized with 1.5% isoflurane in a mixture of 25% O_2_ and 74% N_2_O during surgical procedures. A midline scalp incision was made to expose the skull. Then, craniotomy was conducted over the right parietotemporal cortex (4.0 mm diameter; centered 0.5 mm anterior and 2.0 mm lateral to Bregma) using a motorized drill. CCI was performed using the CCI device with a 3 mm (diameter) flat-tipped impactor to compress the exposed dura mater and underlying brain to a depth of 1.5 mm at a peak velocity of 3.5–3.7 m/s for a dwell time of 150 ms. After CCI, the skull gap was sealed with Koldmount cement (Vernon Benshoff), and the scalp incision was sealed with a 6–0 suture. Rectal temperature was maintained at 37.0°C ± 0.5°C, and heart rate was monitored during surgery.

### Intranasal delivery of antagomir.

The miR-15a/16-1 antagomir (mmu-miR-15a-5p and mmu-miR-16-1-5p, 250 nmol, IDT) and control antagomir (NC-5 control, 250 nmol, IDT) were diluted with RNase-free water. Mice were placed in a supine position on an electric water heating pad (37°C) and anesthetized with 1.5% isoflurane in a mixture of 25% O_2_ and 74% N_2_O during administration processes. To determine the nose-to-brain inhibition efficiency of the antagomir, experimental TBI or sham operations were performed in C57BL/6J mice, and a total of 1 nmol (8.1 μg)/24 μL of miR-15a/16-1 antagomir was intranasally delivered to the mice 2 hours after operation. The expression levels of miR-15a and miR-16-1 were detected by miR assays at the indicated time points (1, 2, 3, 5, and 7 days after operation).

C57BL/6J mice were subjected to TBI or sham operation and intranasally treated with the miR-15a/16-1 antagomir or control antagomir at the indicated time points (2 hours, 5 days, 10 days, 15 days, 20 days, and 25 days after surgery). A total of 1 nmol (8.1 μg)/24 μL of miR-15a/16-1 or control antagomir were alternatively dripped into both nasal cavities of each mouse with a pipette (2 μL/mouse × 12 times, 2-minute interval) as described previously (18, 65). Respiration and body temperature were monitored during drug administration.

### Rotarod test.

Long-term sensorimotor function was evaluated by the rotarod test before and at 3, 5, 7, 14, 21, and 28 days after TBI as described previously (18). Briefly, experimental mice were placed into a rotating drum (IITC Life Science) accelerating from 5 rpm to 40 rpm within 5 minutes. Three trials per day were performed for 3 consecutive days before and up to 28 days after operation with a 5-minute interval between each trial. The mean time on the rod (latency to fall) of 3 trials 1 day before surgery (baseline) and selected time points after operation were recorded.

### Adhesive tape–removal test.

Forepaw sensorimotor function was evaluated by the adhesive tape–removal test as described previously (18). Experimental mice were placed into a transparent Plexiglas cylinder (30 cm tall by 20 cm diameter) for a 60-second habituation period before testing. Then, a piece of adhesive tape (0.3 × 0.4 cm) was placed on the left hairless part of the forepaws. The time when the adhesive tape was touched (time to touch) and completely removed (time to remove) from the forepaw was recorded. Three trials per day were conducted for 3 days before surgery and selected time points after surgery. The maximum time between touch and removal was recorded as 120 seconds if the mouse did not touch or remove the adhesive tape.

### Foot-fault test.

Forepaw and hindpaw sensorimotor functions were examined by the foot-fault test as described previously (30, 66). Mice were allowed to walk freely on a metal grid surface for 3 minutes, and a foot fault was counted when the left forepaw/hindpaw fell or slipped between the wires. One trial per day was conducted for 3 days before surgery and selected time points after the operation. Data were expressed as the percentage of error steps to the total moving steps of the contralateral forepaw/hindpaw.

### MWM test.

Long-term spatial learning and reference memory were evaluated by the MWM test at 22–27 days after TBI as described previously (18, 67, 68). In the place navigation (learning) phase, a circular platform was immersed below the water surface and placed in the middle of the fourth quadrant (target quadrant). Mice were released to the pool from the first, second, and third quadrants in a random order each day, and the times to find the platform (latency to platform) in each trial were recorded. The probe test (memory) on the sixth day was conducted by removing the hidden platform. Mice were released to the pool from the first quadrant and allowed to explore for 60 seconds; the swimming speed and time expended in the target quadrant were recorded. ANYMAZE software and a video recording system were used for recording MWM tests.

### Passive avoidance test.

Long-term cognitive function was also examined by the passive avoidance test as described previously (69, 70). Briefly, in the training phase (29 days after surgery), each mouse was placed into the lightbox and acclimatized for 60 seconds. Then, the door was raised open between the light and dark boxes. After the mouse crossed over the door to the dark side, the door was dropped, and the mouse received a foot shock (0.4–1.6 mA). The mouse was allowed to stay in the dark box for 30 seconds to strengthen the connections between the aversive stimulus and the dark environment. On the testing day (30 days after surgery), the mouse was placed into the light box, and the door was raised open again. Three trials on the testing day were conducted, and the mean time to enter the dark box within 180 seconds in 3 trials was recorded.

### Brain slice preparation.

Experimental mice were deeply anesthetized and transcardially perfused with saline and 4% paraformaldehyde (PFA); then, brains were harvested and postfixed in 4% PFA overnight at 4°C and immersed into 30% sucrose in 0.1M phosphate buffer for 2 days. By using a microtome (Thermo Fisher Scientific, HM450), brains were cut into 25 μm coronal sections. Sections were stored in the cryoprotectant at –20°C until use.

### CV staining.

CV staining was performed to examine TBI-induced neuronal loss (18). Briefly, brain sections were deionized and immersed in CV solution followed by distilled water, 70% alcohol, 95% alcohol, 100% alcohol, and xylene. Three consecutive sections were selected for CV staining, and images of the perilesional CTX, CA1, and CA3 regions from posttrauma brains were taken by using an EVOS M7000 microscope (Thermo Fisher Scientific). CV-stained neurons were calculated using ImageJ software (NIH).

### LFB staining.

LFB staining was conducted to evaluate white matter injury after TBI (18). Briefly, brain sections were immersed in LFB solution, decolored with 70% alcohol and 0.05% Li_2_CO_3_, and dehydrated with graded ethanol. Three consecutive sections were selected for LFB staining, and images of the perilesional CTX, EC, and STR regions from posttrauma brains were taken by using an EVOS M7000 microscope. The LFB relative OD value of each brain region was measured with ImageJ software (NIH).

### IF staining.

IF staining was performed as previously described (18, 71). Briefly, brain sections were permeabilized, blocked and incubated with primary antibodies, and followed by incubation with secondary antibodies accordingly. The primary antibodies used in this study and the corresponding dilution factor and vendor information are listed in Supplemental Table 1. Images were captured by using a confocal microscope (A1R, Nikon). The mean fluorescence intensities of SMI32/MBP, the immunopositive area of GFAP, and the immunopositive cell numbers of NeuN, Iba-1/CD16/32, Iba-1/CD206, F4/80, or Ly-6B were processed with ImageJ software. Three randomly selected microscopic fields on 3 consecutive sections in the pericontusional CTX, CC, EC, STR, and hippocampus were analyzed for each brain. Images for MAP2 immunostaining in 6 coronal brain sections (Bregma: +0.98 mm to –1.58 mm) were taken by using an EVOS M7000 microscope and assessed for evaluation of TBI-induced tissue loss. The NORs were examined by coimmunostaining Caspr with Nav1.6. The number of nodes, paranode length, and length of the paranode gap in the external capsule were counted from 2 microscopic fields as described previously (72).

### Protein extraction and inflammatory antibody array.

Total tissue protein was extracted from the impacted mouse hemisphere at 3 days after TBI or sham operation as described previously (72). A total of 250 μg protein per sample was used to examine the expression of 40 inflammatory mediators by using the RayBiotech mouse inflammatory antibody array kit (AAM-INF-1-8). The relative expression levels of 40 inflammatory mediators were normalized to positive controls in each membrane.

### TaqMan miR assay.

Total RNA was isolated from perilesional or sham-operated brain regions by using a miRNeasy Mini Kit (Qiagen) (73). Reverse transcription was performed using the specific TaqMan MicroRNA Reverse Transcription Kit (Applied Biosystems). PCR reactions were then conducted using the TaqMan MicroRNA Assay Kit (Applied Biosystems). The relative miR levels were normalized to endogenous SnoRNA 202 expression for each sample. PCR experiments were repeated at least 3 times, with each using a separate brain sample.

### Statistics.

The normality of all data in this study was evaluated with the Shapiro-Wilk test. All data in this study are expressed as mean ± SD with dots and analyzed with GraphPad Prism 9 (GraphPad Software). For data that meet with the Shapiro-Wilk normality test and Brown-Forsythe homogeneity of variance test of multiple-group comparisons, 1-way or 2-way ANOVA was used followed by Tukey’s post hoc test. One-way Welch ANOVA and Dunnett T3 post-hoc tests were used when the variances were heterogeneous. The Kruskal-Wallis test was used when data distribution did not meet normal Gaussian distribution. A 2-tailed *t* test was used for the 2-group comparison. Two-tailed Pearson correlation analyses were employed for correlation analysis by “corrplot” and “Hmisc” packages in R project (version 4.1.1). *P* ≤ 0.05 was considered statistically significant. Details of the analysis can be found in the Statistic Reporting section of the Supplemental Materials. 

### Study approval.

All procedures using laboratory animals were approved by both the Department of Veterans Affairs Pittsburgh Healthcare System (VAPHS) and the University of Pittsburgh IACUCs and were conducted consistently with the *Guide for the Care and Use of Laboratory Animals* (National Academies Press, 2011). Male mice were housed in groups of 4 per cage in a temperature- and humidity-controlled animal facility with a 12-hour light-dark cycle. Food and water were available ad libitum. All efforts were made to minimize animal suffering and the number of animals euthanized.

### Data availability.

Supporting information is available from the Wiley Online Library or from the author. All data are available in the main text, supplemental materials, or from the corresponding author upon request. Supporting data for figures can be found in the file of Supporting Data Values.

## Author contributions

KJY and CZ designed this study. CZ, SL, and NQ conducted the experiments. CZ wrote the manuscript. PS acquired and analyzed the data. KJY, MHH, CED, and JC revised this manuscript.

## Supplementary Material

Supplemental data

Unedited blot and gel images

Supporting data values

## Version 1 06/24/2024

Electronic publication
