# Supplementary material for: Loss of microRNA-15a/16-1 function promotes neuropathological and functional recovery in experimental traumatic brain injury
Source: JCI Insight. 2024 Jun 24;9(12):e178650. doi: 10.1172/jci.insight.178650 (PMC11383186; doi:10.1172/jci.insight.178650)
Supplement: Supplemental data [file jciinsight-9-178650-s212.pdf]

Figure	n	Data structure	Test used	Statistic	P value
1A	Sham+WT=12<br>Sham+KO=10<br>TBI+WT=12<br>TBI+KO=12	Normal distribution, homogeneity variances	Two-way repeated ANOVA, Tukey multiple comparisons test	F (3, 42) = 203.4	Two-way repeated ANOVA, p<0.0001<br><br>Tukey's multiple comparisons test:<br><br>Pre:<br>Sham+WT vs. Sham+KO: p=0.9997,<br>Sham+WT vs. TBI+WT: p=0.8449,<br>Sham+WT vs. TBI+KO: p=0.663,<br>Sham+KO vs. TBI+WT: p=0.754,<br>Sham+KO vs. TBI+KO: p=0.5619,<br>TBI+WT vs. TBI+KO: p=0.963<br><br>3d:<br>Sham+WT vs. Sham+KO: p=0.1165,<br>Sham+WT vs. TBI+WT: p<0.0001,<br>Sham+WT vs. TBI+KO: p<0.0001,<br>Sham+KO vs. TBI+WT: p<0.0001,<br>Sham+KO vs. TBI+KO: p<0.0001,<br>TBI+WT vs. TBI+KO: p=0.0003<br><br>5d:<br>Sham+WT vs. Sham+KO: p=0.9401,<br>Sham+WT vs. TBI+WT: p<0.0001,<br>Sham+WT vs. TBI+KO: p<0.0001,<br>Sham+KO vs. TBI+WT: p<0.0001,<br>Sham+KO vs. TBI+KO: p<0.0001,<br>TBI+WT vs. TBI+KO: p=0.4788<br><br>7d:<br>Sham+WT vs. Sham+KO: p>0.9999,<br>Sham+WT vs. TBI+WT: p<0.0001,<br>Sham+WT vs. TBI+KO: p<0.0001,<br>Sham+KO vs. TBI+WT: p<0.0001,<br>Sham+KO vs. TBI+KO: p<0.0001,<br>TBI+WT vs. TBI+KO: p=0.0751<br><br>14d:<br>Sham+WT vs. Sham+KO: p0.9633,<br>Sham+WT vs. TBI+WT: p=<0.0001,<br>Sham+WT vs. TBI+KO: p=<0.0001,<br>Sham+KO vs. TBI+WT: p=<0.0001,<br>Sham+KO vs. TBI+KO: p=<0.0001,<br>TBI+WT vs. TBI+KO: p0.0015<br><br>21d:<br>Sham+WT vs. Sham+KO: p0.175,<br>Sham+WT vs. TBI+WT: p=<0.0001,<br>Sham+WT vs. TBI+KO: p=<0.0001,<br>Sham+KO vs. TBI+WT: p=<0.0001,<br>Sham+KO vs. TBI+KO: p=<0.0001,<br>TBI+WT vs. TBI+KO: p=<0.0001<br><br>28d:<br>Sham+WT vs. Sham+KO: p0.8003,<br>Sham+WT vs. TBI+WT: p=<0.0001,<br>Sham+WT vs. TBI+KO: p=<0.0001,<br>Sham+KO vs. TBI+WT: p=<0.0001,<br>Sham+KO vs. TBI+KO: p=<0.0001,<br>TBI+WT vs. TBI+KO: p0.001
1B	Sham+WT=12<br>Sham+KO=10<br>TBI+WT=12<br>TBI+KO=12	Normal distribution, homogeneity variances	Two-way repeated ANOVA, Tukey multiple comparisons test	F (3, 42) = 94.31	Two-way repeated ANOVA, p<0.0001<br><br>Tukey's multiple comparisons test:<br><br>Pre:<br>Sham+WT vs. Sham+KO: p=0.4384<br>Sham+WT vs. TBI+WT: p=0.4121<br>Sham+WT vs. TBI+KO: p=0.5984<br>Sham+KO vs. TBI+WT: p=0.9996<br>Sham+KO vs. TBI+KO: p=0.9177<br>TBI+WT vs. TBI+KO: p=0.932<br><br>3d:<br>Sham+WT vs. Sham+KO: p=0.9413<br>Sham+WT vs. TBI+WT: p<0.0001<br>Sham+WT vs. TBI+KO: p<0.0001<br>Sham+KO vs. TBI+WT: p<0.0001<br>Sham+KO vs. TBI+KO: p<0.0001<br>TBI+WT vs. TBI+KO: p=0.0053

Figure	n	Data structure	Test used	Statistic	P value
					5d:<br>Sham+WT vs. Sham+KO: p=0.6383<br>Sham+WT vs. TBI+WT: p<0.0001<br>Sham+WT vs. TBI+KO: p<0.0001<br>Sham+KO vs. TBI+WT: p<0.0001<br>Sham+KO vs. TBI+KO: p<0.0001<br>TBI+WT vs. TBI+KO: p=0.0009<br><br>7d:<br>Sham+WT vs. Sham+KO: p=0.8685<br>Sham+WT vs. TBI+WT: p<0.0001<br>Sham+WT vs. TBI+KO: p=0.0001<br>Sham+KO vs. TBI+WT: p<0.0001<br>Sham+KO vs. TBI+KO: p=0.0002<br>TBI+WT vs. TBI+KO: p=0.0014<br><br>14d:<br>Sham+WT vs. Sham+KO: p=0.8231<br>Sham+WT vs. TBI+WT: p<0.0001<br>Sham+WT vs. TBI+KO: p<0.0001<br>Sham+KO vs. TBI+WT: p<0.0001<br>Sham+KO vs. TBI+KO: p=0.0002<br>TBI+WT vs. TBI+KO: p=0.0402<br><br>21d:<br>Sham+WT vs. Sham+KO: p=0.789<br>Sham+WT vs. TBI+WT: p<0.0001<br>Sham+WT vs. TBI+KO: p<0.0001<br>Sham+KO vs. TBI+WT: p<0.0001<br>Sham+KO vs. TBI+KO: p<0.0001<br>TBI+WT vs. TBI+KO: p<0.0001<br><br>28d:<br>Sham+WT vs. Sham+KO: p=0.4226<br>Sham+WT vs. TBI+WT: p<0.0001<br>Sham+WT vs. TBI+KO: p<0.0001<br>Sham+KO vs. TBI+WT: p<0.0001<br>Sham+KO vs. TBI+KO: p<0.0001<br>TBI+WT vs. TBI+KO: p=0.0005
1C	Sham+WT=12<br>Sham+KO=10<br>TBI+WT=12<br>TBI+KO=12	Normal distribution, homogeneity variances	Two-way repeated ANOVA, Tukey multiple comparisons test	F (3, 42) = 195.5	Two-way repeated ANOVA, p<0.0001<br><br>Tukey's multiple comparisons test:<br><br>Pre:<br>Sham+WT vs. Sham+KO: p=0.3453<br>Sham+WT vs. TBI+WT: p=0.1904<br>Sham+WT vs. TBI+KO: p=0.5174<br>Sham+KO vs. TBI+WT: p=0.9935<br>Sham+KO vs. TBI+KO: p=0.8629<br>TBI+WT vs. TBI+KO: p=0.6454<br><br>3d:<br>Sham+WT vs. Sham+KO: p=0.9992<br>Sham+WT vs. TBI+WT: p<0.0001<br>Sham+WT vs. TBI+KO: p<0.0001<br>Sham+KO vs. TBI+WT: p<0.0001<br>Sham+KO vs. TBI+KO: p<0.0001<br>TBI+WT vs. TBI+KO: p=0.0004<br><br>5d:<br>Sham+WT vs. Sham+KO: p=0.6538<br>Sham+WT vs. TBI+WT: p<0.0001<br>Sham+WT vs. TBI+KO: p<0.0001<br>Sham+KO vs. TBI+WT: p<0.0001<br>Sham+KO vs. TBI+KO: p<0.0001<br>TBI+WT vs. TBI+KO: p<0.0001<br><br>7d:<br>Sham+WT vs. Sham+KO: p=0.9961<br>Sham+WT vs. TBI+WT: p<0.0001<br>Sham+WT vs. TBI+KO: p<0.0001<br>Sham+KO vs. TBI+WT: p<0.0001<br>Sham+KO vs. TBI+KO: p<0.0001<br>TBI+WT vs. TBI+KO: p<0.0001<br><br>14d:<br>Sham+WT vs. Sham+KO: p=0.8735

Figure	n	Data structure	Test used	Statistic	P value
					Sham+WT vs. TBI+WT: p<0.0001<br>Sham+WT vs. TBI+KO: p<0.0001<br>Sham+KO vs. TBI+WT: p<0.0001<br>Sham+KO vs. TBI+KO: p<0.0001<br>TBI+WT vs. TBI+KO: p=0.0008<br><br>21d:<br>Sham+WT vs. Sham+KO: p=0.9813<br>Sham+WT vs. TBI+WT: p<0.0001<br>Sham+WT vs. TBI+KO: p<0.0001<br>Sham+KO vs. TBI+WT: p<0.0001<br>Sham+KO vs. TBI+KO: p<0.0001<br>TBI+WT vs. TBI+KO: p=0.0001<br><br>28d:<br>Sham+WT vs. Sham+KO: p=0.9404<br>Sham+WT vs. TBI+WT: p<0.0001<br>Sham+WT vs. TBI+KO: p<0.0001<br>Sham+KO vs. TBI+WT: p<0.0001<br>Sham+KO vs. TBI+KO: p<0.0001<br>TBI+WT vs. TBI+KO: p=0.0033
1D	Sham+WT=12<br>Sham+KO=10<br>TBI+WT=12<br>TBI+KO=12	Normal distribution, homogeneity variances	Two-way repeated ANOVA, Tukey multiple comparisons test	F (3, 42) = 596.3	Two-way repeated ANOVA, p<0.0001<br><br>Tukey's multiple comparisons test:<br><br>Pre:<br>Sham+WT vs. Sham+KO: p=0.9012<br>Sham+WT vs. TBI+WT: p=0.9937<br>Sham+WT vs. TBI+KO: p=0.8869<br>Sham+KO vs. TBI+WT: p=0.763<br>Sham+KO vs. TBI+KO: p=0.9992<br>TBI+WT vs. TBI+KO: p=0.6705<br><br>3d:<br>Sham+WT vs. Sham+KO: p>0.9999<br>Sham+WT vs. TBI+WT: p<0.0001<br>Sham+WT vs. TBI+KO: p<0.0001<br>Sham+KO vs. TBI+WT: p<0.0001<br>Sham+KO vs. TBI+KO: p<0.0001<br>TBI+WT vs. TBI+KO: p=0.0012<br><br>5d:<br>Sham+WT vs. Sham+KO: p=0.744<br>Sham+WT vs. TBI+WT: p<0.0001<br>Sham+WT vs. TBI+KO: p<0.0001<br>Sham+KO vs. TBI+WT: p<0.0001<br>Sham+KO vs. TBI+KO: p<0.0001<br>TBI+WT vs. TBI+KO: p=0.0031<br><br>7d:<br>Sham+WT vs. Sham+KO: p=0.7984<br>Sham+WT vs. TBI+WT: p<0.0001<br>Sham+WT vs. TBI+KO: p<0.0001<br>Sham+KO vs. TBI+WT: p<0.0001<br>Sham+KO vs. TBI+KO: p<0.0001<br>TBI+WT vs. TBI+KO: p<0.0001<br><br>14d:<br>Sham+WT vs. Sham+KO: p=0.8801<br>Sham+WT vs. TBI+WT: p<0.0001<br>Sham+WT vs. TBI+KO: p<0.0001<br>Sham+KO vs. TBI+WT: p<0.0001<br>Sham+KO vs. TBI+KO: p<0.0001<br>TBI+WT vs. TBI+KO: p<0.0001<br><br>21d:<br>Sham+WT vs. Sham+KO: p=0.0373<br>Sham+WT vs. TBI+WT: p<0.0001<br>Sham+WT vs. TBI+KO: p<0.0001<br>Sham+KO vs. TBI+WT: p<0.0001<br>Sham+KO vs. TBI+KO: p<0.0001<br>TBI+WT vs. TBI+KO: p<0.0001<br><br>28d:<br>Sham+WT vs. Sham+KO: p>0.9999<br>Sham+WT vs. TBI+WT: p<0.0001<br>Sham+WT vs. TBI+KO: p<0.0001<br>Sham+KO vs. TBI+WT: p<0.0001

Figure	n	Data structure	Test used	Statistic	P value
					Sham+KO vs. TBI+KO: p<0.0001<br>TBI+WT vs. TBI+KO: p<0.0001
1E	Sham+WT=12<br>Sham+KO=10<br>TBI+WT=12<br>TBI+KO=12	Normal distribution, homogeneity variances	Two-way repeated ANOVA, Tukey multiple comparisons test	F (3, 42) = 686.1	Two-way repeated ANOVA, p<0.0001<br><br>Tukey's multiple comparisons test:<br>Pre:<br>Sham+WT vs. Sham+KO: p=0.5639<br>Sham+WT vs. TBI+WT: p=0.8745<br>Sham+WT vs. TBI+KO: p=0.9975<br>Sham+KO vs. TBI+WT: p=0.8665<br>Sham+KO vs. TBI+KO: p=0.405<br>TBI+WT vs. TBI+KO: p=0.7142<br><br>3d:<br>Sham+WT vs. Sham+KO: p=0.0964<br>Sham+WT vs. TBI+WT: p<0.0001<br>Sham+WT vs. TBI+KO: p<0.0001<br>Sham+KO vs. TBI+WT: p<0.0001<br>Sham+KO vs. TBI+KO: p<0.0001<br>TBI+WT vs. TBI+KO: p=0.0442<br><br>5d:<br>Sham+WT vs. Sham+KO: p=0.3068<br>Sham+WT vs. TBI+WT: p<0.0001<br>Sham+WT vs. TBI+KO: p<0.0001<br>Sham+KO vs. TBI+WT: p<0.0001<br>Sham+KO vs. TBI+KO: p<0.0001<br>TBI+WT vs. TBI+KO: p=0.0749<br><br>7d:<br>Sham+WT vs. Sham+KO: p=0.1316<br>Sham+WT vs. TBI+WT: p<0.0001<br>Sham+WT vs. TBI+KO: p<0.0001<br>Sham+KO vs. TBI+WT: p<0.0001<br>Sham+KO vs. TBI+KO: p<0.0001<br>TBI+WT vs. TBI+KO: p<0.0001<br><br>14d:<br>Sham+WT vs. Sham+KO: p>0.9999<br>Sham+WT vs. TBI+WT: p<0.0001<br>Sham+WT vs. TBI+KO: p<0.0001<br>Sham+KO vs. TBI+WT: p<0.0001<br>Sham+KO vs. TBI+KO: p<0.0001<br>TBI+WT vs. TBI+KO: p=0.006<br><br>21d:<br>Sham+WT vs. Sham+KO: p>0.9999<br>Sham+WT vs. TBI+WT: p<0.0001<br>Sham+WT vs. TBI+KO: p<0.0001<br>Sham+KO vs. TBI+WT: p<0.0001<br>Sham+KO vs. TBI+KO: p<0.0001<br>TBI+WT vs. TBI+KO: p<0.0001<br><br>28d:<br>Sham+WT vs. Sham+KO: p=0.7855<br>Sham+WT vs. TBI+WT: p<0.0001<br>Sham+WT vs. TBI+KO: p<0.0001<br>Sham+KO vs. TBI+WT: p<0.0001<br>Sham+KO vs. TBI+KO: p<0.0001<br>TBI+WT vs. TBI+KO: p<0.0001
1G	Sham+WT=12<br>Sham+KO=10<br>TBI+WT=12<br>TBI+KO=12	Normal distribution, homogeneity variances	Two-way repeated ANOVA, Tukey multiple comparisons test	F (3, 42) = 30.13	Two-way repeated ANOVA, p<0.0001<br><br>Tukey's multiple comparisons test:<br><br>23d:<br>Sham+WT vs. Sham+KO: p=0.8881<br>Sham+WT vs. TBI+WT: p=0.9635<br>Sham+WT vs. TBI+KO: p=0.8362<br>Sham+KO vs. TBI+WT: p=0.706<br>Sham+KO vs. TBI+KO: p=0.5437<br>TBI+WT vs. TBI+KO: p=0.9852<br><br>24d:<br>Sham+WT vs. Sham+KO: p=0.9962<br>Sham+WT vs. TBI+WT: p=0.0832<br>Sham+WT vs. TBI+KO: p=0.976<br>Sham+KO vs. TBI+WT: p=0.031<br>Sham+KO vs. TBI+KO: p=0.8912

Figure	n	Data structure	Test used	Statistic	P value
					TBI+WT vs. TBI+KO: p=0.1429<br><br>25d:<br>Sham+WT vs. Sham+KO: p=0.9854<br>Sham+WT vs. TBI+WT: p=0.0027<br>Sham+WT vs. TBI+KO: p=0.2485<br>Sham+KO vs. TBI+WT: p=0.002<br>Sham+KO vs. TBI+KO: p=0.187<br>TBI+WT vs. TBI+KO: p=0.1582<br><br>26d:<br>Sham+WT vs. Sham+KO: p=0.7178<br>Sham+WT vs. TBI+WT: p=0.0009<br>Sham+WT vs. TBI+KO: p=0.4755<br>Sham+KO vs. TBI+WT: p=0.0002<br>Sham+KO vs. TBI+KO: p=0.1144<br>TBI+WT vs. TBI+KO: p=0.0063<br><br>27d:<br>Sham+WT vs. Sham+KO: p=0.8918<br>Sham+WT vs. TBI+WT: p<0.0001<br>Sham+WT vs. TBI+KO: p=0.0028<br>Sham+KO vs. TBI+WT: p<0.0001<br>Sham+KO vs. TBI+KO: p=0.0308<br>TBI+WT vs. TBI+KO: p=<0.0001
1H	Sham+WT=12<br>Sham+KO=10<br>TBI+WT=12<br>TBI+KO=12	Normal distribution, homogeneity variances	One-way ANOVA, Tukey multiple comparisons test	F (3, 42) = 12.54	One-way ANOVA, p<0.0001<br><br>Tukey's multiple comparisons test:<br><br>Sham+WT vs. Sham+KO: p=0.5536<br>Sham+WT vs. TBI+WT: p=0.0003<br>Sham+WT vs. TBI+KO: p=0.9989<br>Sham+KO vs. TBI+WT: p<0.0001<br>Sham+KO vs. TBI+KO: p=0.4683<br>TBI+WT vs. TBI+KO: p=0.0005
1I	Sham+WT=12<br>Sham+KO=10<br>TBI+WT=12<br>TBI+KO=12	Normal distribution, homogeneity variances	One-way ANOVA, Tukey multiple comparisons test	F (3, 42) = 1.200	One-way ANOVA, p=0.3213<br><br>Tukey's multiple comparisons test:<br><br>Sham+WT vs. Sham+KO: p>0.9999<br>Sham+WT vs. TBI+WT: p=0.3528<br>Sham+WT vs. TBI+KO: p=0.961<br>Sham+KO vs. TBI+WT: p=0.3992<br>Sham+KO vs. TBI+KO: p=0.9676<br>TBI+WT vs. TBI+KO: p=0.6432
1K	Sham+WT=12<br>Sham+KO=10<br>TBI+WT=12<br>TBI+KO=12	Normal distribution, homogeneity variances	Kruskal-Wallis test. Dunn's multiple comparisons test.	Kruskal-Wallis statistic = 23.80	Kruskal-Wallis test, p<0.0001<br><br>Dunn's multiple comparisons test:<br><br>Sham+WT vs. Sham+KO: p>0.9999<br>Sham+WT vs. TBI+WT: p=0.001<br>Sham+WT vs. TBI+KO: p>0.9999<br>Sham+KO vs. TBI+WT: p<0.0001<br>Sham+KO vs. TBI+KO: p=0.5257<br>TBI+WT vs. TBI+KO: p=0.0201
2B	TBI+WT=12<br>TBI+KO=12	Normal distribution, homogeneity variances	Unpaired t test	t=2.348, df=22	TBI+WT vs. TBI+KO: p=0.0283
2C	TBI+WT=12<br>TBI+KO=12	Normal distribution, homogeneity variances	Unpaired t test	t=1.055, df=22<br>t=1.274, df=22<br>t=1.087, df=22<br>t=2.330, df=22<br>t=1.529, df=22<br>t=2.983, df=22	TBI+WT vs. TBI+KO Slice 1: p=0.3031<br>TBI+WT vs. TBI+KO Slice 2: p=0.2160<br>TBI+WT vs. TBI+KO Slice 3: p=0.2888<br>TBI+WT vs. TBI+KO Slice 4: p=0.0294<br>TBI+WT vs. TBI+KO Slice 5: p=0.1404<br>TBI+WT vs. TBI+KO Slice 6: p=0.0069
2F	Sham+WT=6<br>Sham+KO=6<br>TBI+WT=6<br>TBI+KO=6	Normal distribution, homogeneity variances	One-way ANOVA, Tukey multiple comparisons test	F (3, 20) = 102.9	One-way ANOVA, p<0.0001<br><br>Tukey's multiple comparisons test:<br><br>Sham+WT vs. Sham+KO: p=0.9998<br>Sham+WT vs. TBI+WT: p<0.0001<br>Sham+WT vs. TBI+KO: p<0.0001<br>Sham+KO vs. TBI+WT: p<0.0001<br>Sham+KO vs. TBI+KO: p<0.0001<br>TBI+WT vs. TBI+KO: p=0.0002
2G	Sham+WT=6<br>Sham+KO=6<br>TBI+WT=6	Normal distribution, homogeneity	One-way ANOVA, Tukey	F (3, 20) = 17.20	One-way ANOVA, p<0.0001<br><br>Tukey's multiple comparisons test:

Figure	n	Data structure	Test used	Statistic	P value
	TBI+KO=6	variances	multiple comparisons test		Sham+WT vs. Sham+KO: p=0.7326<br>Sham+WT vs. TBI+WT: p=0.0001<br>Sham+WT vs. TBI+KO: p=0.0713<br>Sham+KO vs. TBI+WT: p<0.0001<br>Sham+KO vs. TBI+KO: p=0.0079<br>TBI+WT vs. TBI+KO: p=0.0405
2H	Sham+WT=6<br>Sham+KO=6<br>TBI+WT=6<br>TBI+KO=6	Normal distribution, homogeneity variances	One-way ANOVA, Tukey multiple comparisons test	F (3, 20) = 2.082	One-way ANOVA, p= 0.1347<br><br>Tukey's multiple comparisons test:<br><br>Sham+WT vs. Sham+KO: p=0.7447<br>Sham+WT vs. TBI+WT: p=0.1574<br>Sham+WT vs. TBI+KO: p=0.2086<br>Sham+KO vs. TBI+WT: p=0.6408<br>Sham+KO vs. TBI+KO: p=0.7375<br>TBI+WT vs. TBI+KO: p=0.9984
2J	Sham+WT=6<br>Sham+KO=6<br>TBI+WT=6<br>TBI+KO=6	Normal distribution, homogeneity variances	One-way ANOVA, Tukey multiple comparisons test	F (3, 20) = 19.23	One-way ANOVA, p<0.0001<br><br>Tukey's multiple comparisons test:<br><br>Sham+WT vs. Sham+KO: p=0.9994<br>Sham+WT vs. TBI+WT: p<0.0001<br>Sham+WT vs. TBI+KO: p=0.0129<br>Sham+KO vs. TBI+WT: p<0.0001<br>Sham+KO vs. TBI+KO: p=0.0099<br>TBI+WT vs. TBI+KO: p=0.0385
2K	Sham+WT=6<br>Sham+KO=6<br>TBI+WT=6<br>TBI+KO=6	Normal distribution, homogeneity variances	One-way ANOVA, Tukey multiple comparisons test	F (3, 20) = 73.23	One-way ANOVA, p<0.0001<br><br>Tukey's multiple comparisons test:<br><br>Sham+WT vs. Sham+KO: p=0.9942<br>Sham+WT vs. TBI+WT: p<0.0001<br>Sham+WT vs. TBI+KO: p<0.0001<br>Sham+KO vs. TBI+WT: p<0.0001<br>Sham+KO vs. TBI+KO: p<0.0001<br>TBI+WT vs. TBI+KO: p=0.0001
2L	Sham+WT=6<br>Sham+KO=6<br>TBI+WT=6<br>TBI+KO=6	Normal distribution, homogeneity variances	One-way ANOVA, Tukey multiple comparisons test	F (3, 20) = 1.510	One-way ANOVA, p=0.2425<br><br>Tukey's multiple comparisons test:<br><br>Sham+WT vs. Sham+KO: p=0.6022<br>Sham+WT vs. TBI+WT: p=0.9905<br>Sham+WT vs. TBI+KO: p=0.8608<br>Sham+KO vs. TBI+WT: p=0.4278<br>Sham+KO vs. TBI+KO: p=0.2083<br>TBI+WT vs. TBI+KO: p=0.9613
3C	Sham+WT=6<br>Sham+KO=6<br>TBI+WT=6<br>TBI+KO=6	Normal distribution, homogeneity variances	One-way ANOVA, Tukey	F (3, 20) = 269.4	One-way ANOVA, p<0.0001<br><br>Tukey's multiple comparisons test:<br><br>Sham+WT vs. Sham+KO: p=0.0729<br>Sham+WT vs. TBI+WT: p<0.0001<br>Sham+WT vs. TBI+KO: p<0.0001<br>Sham+KO vs. TBI+WT: p<0.0001<br>Sham+KO vs. TBI+KO: p<0.0001<br>TBI+WT vs. TBI+KO: p=0.0018
3D	Sham+WT=6<br>Sham+KO=6<br>TBI+WT=6<br>TBI+KO=6	Normal distribution, homogeneity variances	One-way ANOVA, Tukey multiple comparisons test	F (3, 20) = 45.91	One-way ANOVA, p<0.0001<br><br>Tukey's multiple comparisons test:<br><br>Sham+WT vs. Sham+KO: p=0.9421<br>Sham+WT vs. TBI+WT: p<0.0001<br>Sham+WT vs. TBI+KO: p=0.0006<br>Sham+KO vs. TBI+WT: p<0.0001<br>Sham+KO vs. TBI+KO: p=0.0002<br>TBI+WT vs. TBI+KO: p=0.0005
3E	Sham+WT=6<br>Sham+KO=6<br>TBI+WT=6<br>TBI+KO=6	Normal distribution, homogeneity variances	One-way ANOVA, Tukey multiple comparisons test	F (3, 20) = 55.27	One-way ANOVA, p<0.0001<br><br>Tukey's multiple comparisons test:<br><br>Sham+WT vs. Sham+KO: p=0.9572<br>Sham+WT vs. TBI+WT: p<0.0001<br>Sham+WT vs. TBI+KO: p=0.0231<br>Sham+KO vs. TBI+WT: p<0.0001<br>Sham+KO vs. TBI+KO: p=0.0659<br>TBI+WT vs. TBI+KO: p<0.0001

Figure	n	Data structure	Test used	Statistic	P value
3G	Sham+WT=6<br>Sham+KO=6<br>TBI+WT=6<br>TBI+KO=6	Normal distribution, homogeneity variances	One-way ANOVA, Tukey multiple comparisons test	F (3, 20) = 35.74	One-way ANOVA, p<0.0001<br><br>Tukey's multiple comparisons test:<br><br>Sham+WT vs. Sham+KO: p=0.9628<br>Sham+WT vs. TBI+WT: p<0.0001<br>Sham+WT vs. TBI+KO: p=0.0006<br>Sham+KO vs. TBI+WT: p<0.0001<br>Sham+KO vs. TBI+KO: p=0.0019<br>TBI+WT vs. TBI+KO: p=0.002
3H	Sham+WT=6<br>Sham+KO=6<br>TBI+WT=6<br>TBI+KO=6	Normal distribution, homogeneity variances	One-way ANOVA, Tukey multiple comparisons test	F (3, 20) = 37.51	One-way ANOVA, p<0.0001<br><br>Tukey's multiple comparisons test:<br><br>Sham+WT vs. Sham+KO: p=0.498<br>Sham+WT vs. TBI+WT: p<0.0001<br>Sham+WT vs. TBI+KO: p=0.003<br>Sham+KO vs. TBI+WT: p<0.0001<br>Sham+KO vs. TBI+KO: p=0.0671<br>TBI+WT vs. TBI+KO: p=<0.0001
3I	Sham+WT=6<br>Sham+KO=6<br>TBI+WT=6<br>TBI+KO=6	Normal distribution, homogeneity variances	One-way ANOVA, Tukey multiple comparisons test	F (3, 20) = 35.16	One-way ANOVA, p<0.0001<br><br>Tukey's multiple comparisons test:<br><br>Sham+WT vs. Sham+KO: p=0.3926<br>Sham+WT vs. TBI+WT: p<0.0001<br>Sham+WT vs. TBI+KO: p<0.0001<br>Sham+KO vs. TBI+WT: p<0.0001<br>Sham+KO vs. TBI+KO: p=0.0008<br>TBI+WT vs. TBI+KO: p=0.0459
3J	Sham+WT=6<br>Sham+KO=6<br>TBI+WT=6<br>TBI+KO=6	Normal distribution, homogeneity variances	One-way ANOVA, Tukey multiple comparisons test	F (3, 20) = 39.82	One-way ANOVA, p<0.0001<br><br>Tukey's multiple comparisons test:<br><br>Sham+WT vs. Sham+KO: p=0.8951<br>Sham+WT vs. TBI+WT: p<0.0001<br>Sham+WT vs. TBI+KO: p=0.4235<br>Sham+KO vs. TBI+WT: p<0.0001<br>Sham+KO vs. TBI+KO: p=0.1414<br>TBI+WT vs. TBI+KO: p=<0.0001
3K	Sham+WT=6<br>Sham+KO=6<br>TBI+WT=6<br>TBI+KO=6	Normal distribution, homogeneity variances	One-way ANOVA, Tukey multiple comparisons test	F (3, 20) = 39.51	One-way ANOVA, p<0.0001<br><br>Tukey's multiple comparisons test:<br><br>Sham+WT vs. Sham+KO: p=0.9533<br>Sham+WT vs. TBI+WT: p<0.0001<br>Sham+WT vs. TBI+KO: p=0.9177<br>Sham+KO vs. TBI+WT: p<0.0001<br>Sham+KO vs. TBI+KO: p=0.658<br>TBI+WT vs. TBI+KO: p=<0.0001
3L	Sham+WT=6<br>Sham+KO=6<br>TBI+WT=6<br>TBI+KO=6	Normal distribution, homogeneity variances	One-way ANOVA, Tukey multiple comparisons test	F (3, 20) = 19.82	One-way ANOVA, p<0.0001<br><br>Tukey's multiple comparisons test:<br>Sham+WT vs. Sham+KO: p=0.9134<br>Sham+WT vs. TBI+WT: p<0.0001<br>Sham+WT vs. TBI+KO: p=0.1835<br>Sham+KO vs. TBI+WT: p<0.0001<br>Sham+KO vs. TBI+KO: p=0.0537<br>TBI+WT vs. TBI+KO: p=0.0022
3O	Sham+WT=6<br>Sham+KO=6<br>TBI+WT=6<br>TBI+KO=6	Normal distribution, homogeneity variances	One-way ANOVA, Tukey multiple comparisons test	F (3, 20) = 116.5	One-way ANOVA, p<0.0001<br><br>Tukey's multiple comparisons test:<br><br>Sham+WT vs. Sham+KO: p=0.8034<br>Sham+WT vs. TBI+WT: p<0.0001<br>Sham+WT vs. TBI+KO: p<0.0001<br>Sham+KO vs. TBI+WT: p<0.0001<br>Sham+KO vs. TBI+KO: p<0.0001<br>TBI+WT vs. TBI+KO: p<0.0001
3P	Sham+WT=6<br>Sham+KO=6<br>TBI+WT=6<br>TBI+KO=6	Normal distribution, homogeneity variances	One-way ANOVA, Tukey multiple comparisons test	F (3, 20) = 59.00	One-way ANOVA, p<0.0001<br><br>Tukey's multiple comparisons test:<br><br>Sham+WT vs. Sham+KO: p=0.5602<br>Sham+WT vs. TBI+WT: p<0.0001<br>Sham+WT vs. TBI+KO: p<0.0001<br>Sham+KO vs. TBI+WT: p<0.0001<br>Sham+KO vs. TBI+KO: p0.0011

Figure	n	Data structure	Test used	Statistic	P value
					TBI+WT vs. TBI+KO: p<0.0001
3Q	Sham+WT=6<br>Sham+KO=6<br>TBI+WT=6<br>TBI+KO=6	Normal distribution, homogeneity variances	One-way ANOVA, Tukey multiple comparisons test	F (3, 20) = 4.120	One-way ANOVA, p<0.0001<br><br>Tukey's multiple comparisons test:<br><br>Sham+WT vs. Sham+KO: p=0.9988<br>Sham+WT vs. TBI+WT: p=0.0361<br>Sham+WT vs. TBI+KO: p=0.6427<br>Sham+KO vs. TBI+WT: p=0.0264<br>Sham+KO vs. TBI+KO: p=0.5535<br>TBI+WT vs. TBI+KO: p=0.3156
4D	Sham+WT=6<br>Sham+KO=6<br>TBI+WT=6<br>TBI+KO=6	Normal distribution, homogeneity variances	One-way ANOVA, Tukey multiple comparisons test	F (3, 20) = 107.3	One-way ANOVA, p<0.0001<br><br>Tukey's multiple comparisons test:<br><br>Sham+WT vs. Sham+KO: p>0.9999<br>Sham+WT vs. TBI+WT: p<0.0001<br>Sham+WT vs. TBI+KO: p=<0.0001<br>Sham+KO vs. TBI+WT: p<0.0001<br>Sham+KO vs. TBI+KO: p=<0.0001<br>TBI+WT vs. TBI+KO: p=<0.0001
4E	Sham+WT=6<br>Sham+KO=6<br>TBI+WT=6<br>TBI+KO=6	Normal distribution, homogeneity variances	One-way ANOVA, Tukey multiple comparisons test	F (3, 20) = 102.3	One-way ANOVA, p<0.0001<br><br>Tukey's multiple comparisons test:<br><br>Sham+WT vs. Sham+KO: p>0.9999<br>Sham+WT vs. TBI+WT: p<0.0001<br>Sham+WT vs. TBI+KO: p=0.0002<br>Sham+KO vs. TBI+WT: p<0.0001<br>Sham+KO vs. TBI+KO: p=0.0002<br>TBI+WT vs. TBI+KO: p<0.0001
4F	Sham+WT=6<br>Sham+KO=6<br>TBI+WT=6<br>TBI+KO=6	Normal distribution, homogeneity variances	One-way ANOVA, Tukey multiple comparisons test	F (3, 20) = 102.8	One-way ANOVA, p<0.0001<br><br>Tukey's multiple comparisons test:<br><br>Sham+WT vs. Sham+KO: p>0.9999<br>Sham+WT vs. TBI+WT: p<0.0001<br>Sham+WT vs. TBI+KO: p<0.0001<br>Sham+KO vs. TBI+WT: p<0.0001<br>Sham+KO vs. TBI+KO: p<0.0001<br>TBI+WT vs. TBI+KO: p<0.0001
4I	Sham+WT=6<br>Sham+KO=6<br>TBI+WT=6<br>TBI+KO=6	Normal distribution, homogeneity variances	One-way ANOVA, Tukey multiple comparisons test	F (3, 20) = 176.8	One-way ANOVA, p<0.0001<br><br>Tukey's multiple comparisons test:<br><br>Sham+WT vs. Sham+KO: p>0.9999<br>Sham+WT vs. TBI+WT: p<0.0001<br>Sham+WT vs. TBI+KO: p<0.0001<br>Sham+KO vs. TBI+WT: p<0.0001<br>Sham+KO vs. TBI+KO: p<0.0001<br>TBI+WT vs. TBI+KO: p<0.0001
4J	Sham+WT=6<br>Sham+KO=6<br>TBI+WT=6<br>TBI+KO=6	Normal distribution, homogeneity variances	One-way ANOVA, Tukey multiple comparisons test	F (3, 20) = 286.4	One-way ANOVA, p<0.0001<br><br>Tukey's multiple comparisons test:<br><br>Sham+WT vs. Sham+KO: p>0.9999<br>Sham+WT vs. TBI+WT: p<0.0001<br>Sham+WT vs. TBI+KO: p<0.0001<br>Sham+KO vs. TBI+WT: p<0.0001<br>Sham+KO vs. TBI+KO: p<0.0001<br>TBI+WT vs. TBI+KO: p<0.0001
4M	Sham+WT=6<br>Sham+KO=6<br>TBI+WT=6<br>TBI+KO=6	Normal distribution, homogeneity variances	One-way ANOVA, Tukey multiple comparisons test	F (3, 20) = 523.3	One-way ANOVA, p<0.0001<br><br>Tukey's multiple comparisons test:<br><br>Sham+WT vs. Sham+KO: p>0.9999<br>Sham+WT vs. TBI+WT: p<0.0001<br>Sham+WT vs. TBI+KO: p<0.0001<br>Sham+KO vs. TBI+WT: p<0.0001<br>Sham+KO vs. TBI+KO: p<0.0001<br>TBI+WT vs. TBI+KO: p<0.0001
4N	Sham+WT=6<br>Sham+KO=6<br>TBI+WT=6<br>TBI+KO=6	Normal distribution, homogeneity variances	One-way ANOVA, Tukey multiple comparisons test	F (3, 20) = 166.0	One-way ANOVA, p<0.0001<br><br>Tukey's multiple comparisons test:<br><br>Sham+WT vs. Sham+KO: p>0.9999<br>Sham+WT vs. TBI+WT: p<0.0001

Figure	n	Data structure	Test used	Statistic	P value
					Sham+WT vs. TBI+KO: p<0.0001<br>Sham+KO vs. TBI+WT: p<0.0001<br>Sham+KO vs. TBI+KO: p<0.0001<br>TBI+WT vs. TBI+KO: p<0.0001
5A	Sham+control=10<br>Sham+antagomir=10<br>TBI+control=10<br>TBI+antagomir=10	Normal distribution, homogeneity variances	Two-way repeated ANOVA, Tukey multiple comparisons test	F (3, 36) = 185.0	Two-way repeated ANOVA, p<0.0001<br><br>Tukey's multiple comparisons test:<br><br>Pre:<br>Sham+control vs. Sham+antagomir: p=0.4362<br>Sham+control vs. TBI+control: p>0.9999<br>Sham+control vs. TBI+antagomir: p>0.9999<br>Sham+antagomir vs. TBI+control: p=0.507<br>Sham+antagomir vs. TBI+antagomir: p=0.4791<br>TBI+control vs. TBI+antagomir: p=0.9997<br><br>3d:<br>Sham+control vs. Sham+antagomir: p=0.9831<br>Sham+control vs. TBI+control: p<0.0001<br>Sham+control vs. TBI+antagomir: p<0.0001<br>Sham+antagomir vs. TBI+control: p<0.0001<br>Sham+antagomir vs. TBI+antagomir: p<0.0001<br>TBI+control vs. TBI+antagomir: p=0.0003<br><br>5d:<br>Sham+control vs. Sham+antagomir: p=0.3015<br>Sham+control vs. TBI+control: p<0.0001<br>Sham+control vs. TBI+antagomir: p<0.0001<br>Sham+antagomir vs. TBI+control: p<0.0001<br>Sham+antagomir vs. TBI+antagomir: p<0.0001<br>TBI+control vs. TBI+antagomir: p=0.0003<br><br>7d:<br>Sham+control vs. Sham+antagomir: p=0.9592<br>Sham+control vs. TBI+control: p<0.0001<br>Sham+control vs. TBI+antagomir: p<0.0001<br>Sham+antagomir vs. TBI+control: p<0.0001<br>Sham+antagomir vs. TBI+antagomir: p<0.0001<br>TBI+control vs. TBI+antagomir: p=0.0057<br><br>14d:<br>Sham+control vs. Sham+antagomir: p=0.4445<br>Sham+control vs. TBI+control: p<0.0001<br>Sham+control vs. TBI+antagomir: p<0.0001<br>Sham+antagomir vs. TBI+control: p<0.0001<br>Sham+antagomir vs. TBI+antagomir: p<0.0001<br>TBI+control vs. TBI+antagomir: p=0.0088<br><br>21d:<br>Sham+control vs. Sham+antagomir: p=0.9863<br>Sham+control vs. TBI+control: p<0.0001<br>Sham+control vs. TBI+antagomir: p<0.0001<br>Sham+antagomir vs. TBI+control: p<0.0001<br>Sham+antagomir vs. TBI+antagomir: p<0.0001<br>TBI+control vs. TBI+antagomir: p=0.0031<br><br>28d:<br>Sham+control vs. Sham+antagomir: p=0.9894<br>Sham+control vs. TBI+control: p<0.0001<br>Sham+control vs. TBI+antagomir: p<0.0001<br>Sham+antagomir vs. TBI+control: p<0.0001<br>Sham+antagomir vs. TBI+antagomir: p<0.0001<br>TBI+control vs. TBI+antagomir: p=0.0014
5B	Sham+control=10<br>Sham+antagomir=10<br>TBI+control=10<br>TBI+antagomir=10	Normal distribution, homogeneity variances	Two-way repeated ANOVA, Tukey multiple comparisons test	F (3, 36) = 540.2	Two-way repeated ANOVA, p<0.0001<br><br>Tukey's multiple comparisons test:<br><br>Pre:<br>Sham+control vs. Sham+antagomir: p=0.8123<br>Sham+control vs. TBI+control: p=0.3724<br>Sham+control vs. TBI+antagomir: p=0.667<br>Sham+antagomir vs. TBI+control: p=0.9004<br>Sham+antagomir vs. TBI+antagomir: p=0.9988<br>TBI+control vs. TBI+antagomir: p=0.9277<br><br>3d:<br>Sham+control vs. Sham+antagomir: p=0.9256<br>Sham+control vs. TBI+control: p<0.0001

Figure	n	Data structure	Test used	Statistic	P value
					Sham+control vs. TBI+antagomir: p<0.0001<br>Sham+antagomir vs. TBI+control: p<0.0001<br>Sham+antagomir vs. TBI+antagomir: p<0.0001<br>TBI+control vs. TBI+antagomir: p<0.0001<br><br>5d:<br>Sham+control vs. Sham+antagomir: p=0.9904<br>Sham+control vs. TBI+control: p<0.0001<br>Sham+control vs. TBI+antagomir: p<0.0001<br>Sham+antagomir vs. TBI+control: p<0.0001<br>Sham+antagomir vs. TBI+antagomir: p<0.0001<br>TBI+control vs. TBI+antagomir: p<0.0001<br><br>7d:<br>Sham+control vs. Sham+antagomir: p=0.9993<br>Sham+control vs. TBI+control: p<0.0001<br>Sham+control vs. TBI+antagomir: p<0.0001<br>Sham+antagomir vs. TBI+control: p<0.0001<br>Sham+antagomir vs. TBI+antagomir: p<0.0001<br>TBI+control vs. TBI+antagomir: p<0.0001<br><br>14d:<br>Sham+control vs. Sham+antagomir: p=0.9059<br>Sham+control vs. TBI+control: p<0.0001<br>Sham+control vs. TBI+antagomir: p<0.0001<br>Sham+antagomir vs. TBI+control: p<0.0001<br>Sham+antagomir vs. TBI+antagomir: p<0.0001<br>TBI+control vs. TBI+antagomir: p<0.0001<br><br>21d:<br>Sham+control vs. Sham+antagomir: p=0.9156<br>Sham+control vs. TBI+control: p<0.0001<br>Sham+control vs. TBI+antagomir: p<0.0001<br>Sham+antagomir vs. TBI+control: p<0.0001<br>Sham+antagomir vs. TBI+antagomir: p<0.0001<br>TBI+control vs. TBI+antagomir: p=0.0009<br><br>28d:<br>Sham+control vs. Sham+antagomir: p>0.9999<br>Sham+control vs. TBI+control: p<0.0001<br>Sham+control vs. TBI+antagomir: p<0.0001<br>Sham+antagomir vs. TBI+control: p<0.0001<br>Sham+antagomir vs. TBI+antagomir: p<0.0001<br>TBI+control vs. TBI+antagomir: p=0.0005
5C	Sham+control=10<br>Sham+antagomir=10<br>TBI+control=10<br>TBI+antagomir=10	Normal distribution, homogeneity variances	Two-way repeated ANOVA, Tukey multiple comparisons test	F (3, 36) = 574.6	Two-way repeated ANOVA, p<0.0001<br><br>Tukey's multiple comparisons test:<br><br>Pre:<br>Sham+control vs. Sham+antagomir: p>0.9999<br>Sham+control vs. TBI+control: p=0.9532<br>Sham+control vs. TBI+antagomir: p>0.9999<br>Sham+antagomir vs. TBI+control: p=0.9102<br>Sham+antagomir vs. TBI+antagomir: p>0.9999<br>TBI+control vs. TBI+antagomir: p=0.9103<br><br>3d:<br>Sham+control vs. Sham+antagomir: p=0.828<br>Sham+control vs. TBI+control: p<0.0001<br>Sham+control vs. TBI+antagomir: p<0.0001<br>Sham+antagomir vs. TBI+control: p<0.0001<br>Sham+antagomir vs. TBI+antagomir: p<0.0001<br>TBI+control vs. TBI+antagomir: p<0.0001<br><br>5d:<br>Sham+control vs. Sham+antagomir: p=0.5959<br>Sham+control vs. TBI+control: p<0.0001<br>Sham+control vs. TBI+antagomir: p<0.0001<br>Sham+antagomir vs. TBI+control: p<0.0001<br>Sham+antagomir vs. TBI+antagomir: p<0.0001<br>TBI+control vs. TBI+antagomir: p<0.0001<br><br>7d:<br>Sham+control vs. Sham+antagomir: p=0.6212<br>Sham+control vs. TBI+control: p<0.0001<br>Sham+control vs. TBI+antagomir: p<0.0001<br>Sham+antagomir vs. TBI+control: p<0.0001<br>Sham+antagomir vs. TBI+antagomir: p<0.0001

Figure	n	Data structure	Test used	Statistic	P value
					TBI+control vs. TBI+antagomir: p<0.0001<br><br>14d:<br>Sham+control vs. Sham+antagomir: p=0.5845<br>Sham+control vs. TBI+control: p<0.0001<br>Sham+control vs. TBI+antagomir: p<0.0001<br>Sham+antagomir vs. TBI+control: p<0.0001<br>Sham+antagomir vs. TBI+antagomir: p<0.0001<br>TBI+control vs. TBI+antagomir: p=0.0001<br><br>21d:<br>Sham+control vs. Sham+antagomir: p=0.8475<br>Sham+control vs. TBI+control: p<0.0001<br>Sham+control vs. TBI+antagomir: p<0.0001<br>Sham+antagomir vs. TBI+control: p<0.0001<br>Sham+antagomir vs. TBI+antagomir: p<0.0001<br>TBI+control vs. TBI+antagomir: p=0.0002<br><br>28d:<br>Sham+control vs. Sham+antagomir: p0.946<br>Sham+control vs. TBI+control: p<0.0001<br>Sham+control vs. TBI+antagomir: p<0.0001<br>Sham+antagomir vs. TBI+control: p<0.0001<br>Sham+antagomir vs. TBI+antagomir: p<0.0001<br>TBI+control vs. TBI+antagomir: p<0.0001
5D	Sham+control=10<br>Sham+antagomir=10<br>TBI+control=10<br>TBI+antagomir=10	Normal distribution, homogeneity variances	Two-way repeated ANOVA, Tukey multiple comparisons test	F (3, 36) = 955.2	Two-way repeated ANOVA, p<0.0001<br><br>Tukey's multiple comparisons test:<br><br>Pre:<br>Sham+control vs. Sham+antagomir: p=0.9076<br>Sham+control vs. TBI+control: p>0.9999<br>Sham+control vs. TBI+antagomir: p=0.7505<br>Sham+antagomir vs. TBI+control: p=0.8624<br>Sham+antagomir vs. TBI+antagomir: p=0.9846<br>TBI+control vs. TBI+antagomir: p=0.6602<br><br>3d:<br>Sham+control vs. Sham+antagomir: p>0.9999<br>Sham+control vs. TBI+control: p<0.0001<br>Sham+control vs. TBI+antagomir: p<0.0001<br>Sham+antagomir vs. TBI+control: p<0.0001<br>Sham+antagomir vs. TBI+antagomir: p<0.0001<br>TBI+control vs. TBI+antagomir: p=0.0161<br><br>5d:<br>Sham+control vs. Sham+antagomir: p=0.3139<br>Sham+control vs. TBI+control: p<0.0001<br>Sham+control vs. TBI+antagomir: p<0.0001<br>Sham+antagomir vs. TBI+control: p<0.0001<br>Sham+antagomir vs. TBI+antagomir: p<0.0001<br>TBI+control vs. TBI+antagomir: p=0.0009<br><br>7d:<br>Sham+control vs. Sham+antagomir: p=0.6088<br>Sham+control vs. TBI+control: p<0.0001<br>Sham+control vs. TBI+antagomir: p<0.0001<br>Sham+antagomir vs. TBI+control: p<0.0001<br>Sham+antagomir vs. TBI+antagomir: p<0.0001<br>TBI+control vs. TBI+antagomir: p=0.0019<br><br>14d:<br>Sham+control vs. Sham+antagomir: p=0.7498<br>Sham+control vs. TBI+control: p<0.0001<br>Sham+control vs. TBI+antagomir: p<0.0001<br>Sham+antagomir vs. TBI+control: p<0.0001<br>Sham+antagomir vs. TBI+antagomir: p<0.0001<br>TBI+control vs. TBI+antagomir: p=0.0002<br><br>21d:<br>Sham+control vs. Sham+antagomir: p=0.9937<br>Sham+control vs. TBI+control: p<0.0001<br>Sham+control vs. TBI+antagomir: p<0.0001<br>Sham+antagomir vs. TBI+control: p<0.0001<br>Sham+antagomir vs. TBI+antagomir: p<0.0001<br>TBI+control vs. TBI+antagomir: p=<0.0001<br><br>28d:

Figure	n	Data structure	Test used	Statistic	P value
					Sham+control vs. Sham+antagomir: p=0.7877<br>Sham+control vs. TBI+control: p<0.0001<br>Sham+control vs. TBI+antagomir: p<0.0001<br>Sham+antagomir vs. TBI+control: p<0.0001<br>Sham+antagomir vs. TBI+antagomir: p<0.0001<br>TBI+control vs. TBI+antagomir: p<0.0001
5E	Sham+control=10<br>Sham+antagomir=10<br>TBI+control=10<br>TBI+antagomir=10	Normal distribution, homogeneity variances n	Two-way repeated ANOVA, Tukey multiple comparisons test	F (3, 36) = 813.1	Two-way repeated ANOVA, p<0.0001<br><br>Tukey's multiple comparisons test:<br><br>Pre:<br>Sham+control vs. Sham+antagomir: p>0.9999<br>Sham+control vs. TBI+control: p=0.6688<br>Sham+control vs. TBI+antagomir: p=0.5535<br>Sham+antagomir vs. TBI+control: p=0.8379<br>Sham+antagomir vs. TBI+antagomir: p=0.754<br>TBI+control vs. TBI+antagomir: p=0.9964<br><br>3d:<br>Sham+control vs. Sham+antagomir: p=0.6525<br>Sham+control vs. TBI+control: p<0.0001<br>Sham+control vs. TBI+antagomir: p<0.0001<br>Sham+antagomir vs. TBI+control: p<0.0001<br>Sham+antagomir vs. TBI+antagomir: p<0.0001<br>TBI+control vs. TBI+antagomir: p=0.0013<br><br>5d:<br>Sham+control vs. Sham+antagomir: p=0.6765<br>Sham+control vs. TBI+control: p<0.0001<br>Sham+control vs. TBI+antagomir: p<0.0001<br>Sham+antagomir vs. TBI+control: p<0.0001<br>Sham+antagomir vs. TBI+antagomir: p<0.0001<br>TBI+control vs. TBI+antagomir: p=0.0083<br><br>7d:<br>Sham+control vs. Sham+antagomir: p=0.9421<br>Sham+control vs. TBI+control: p<0.0001<br>Sham+control vs. TBI+antagomir: p<0.0001<br>Sham+antagomir vs. TBI+control: p<0.0001<br>Sham+antagomir vs. TBI+antagomir: p<0.0001<br>TBI+control vs. TBI+antagomir: p=0.0302<br><br>14d:<br>Sham+control vs. Sham+antagomir: p=0.7063<br>Sham+control vs. TBI+control: p<0.0001<br>Sham+control vs. TBI+antagomir: p<0.0001<br>Sham+antagomir vs. TBI+control: p<0.0001<br>Sham+antagomir vs. TBI+antagomir: p<0.0001<br>TBI+control vs. TBI+antagomir: p=0.0004<br><br>21d:<br>Sham+control vs. Sham+antagomir: p=0.9938<br>Sham+control vs. TBI+control: p<0.0001<br>Sham+control vs. TBI+antagomir: p<0.0001<br>Sham+antagomir vs. TBI+control: p<0.0001<br>Sham+antagomir vs. TBI+antagomir: p<0.0001<br>TBI+control vs. TBI+antagomir: p=0.0005<br><br>28d:<br>Sham+control vs. Sham+antagomir: p=0.8951<br>Sham+control vs. TBI+control: p<0.0001<br>Sham+control vs. TBI+antagomir: p<0.0001<br>Sham+antagomir vs. TBI+control: p<0.0001<br>Sham+antagomir vs. TBI+antagomir: p<0.0001<br>TBI+control vs. TBI+antagomir: p<0.0001
5G	Sham+control=10<br>Sham+antagomir=10<br>TBI+control=10<br>TBI+antagomir=10	Normal distribution, homogeneity variances	Two-way repeated ANOVA, Tukey multiple comparisons test	F (3, 36) = 51.17	Two-way repeated ANOVA, p<0.0001<br><br>Tukey's multiple comparisons test:<br><br>22d:<br>Sham+control vs. Sham+antagomir: p=0.8242<br>Sham+control vs. TBI+control: p=0.1248<br>Sham+control vs. TBI+antagomir: p=0.9998<br>Sham+antagomir vs. TBI+control: p=0.0591<br>Sham+antagomir vs. TBI+antagomir: p=0.8739<br>TBI+control vs. TBI+antagomir: p=0.1509<br><br>33d:

Figure	n	Data structure	Test used	Statistic	P value
					Sham+control vs. Sham+antagomir: p=0.9996<br>Sham+control vs. TBI+control: p=0.0001<br>Sham+control vs. TBI+antagomir: p=0.1825<br>Sham+antagomir vs. TBI+control: p=0.0017<br>Sham+antagomir vs. TBI+antagomir: p=0.2739<br>TBI+control vs. TBI+antagomir: p=0.006<br><br>24d:<br>Sham+control vs. Sham+antagomir: p=0.4085<br>Sham+control vs. TBI+control: p<0.0001<br>Sham+control vs. TBI+antagomir: p=0.2019<br>Sham+antagomir vs. TBI+control: p<0.0001<br>Sham+antagomir vs. TBI+antagomir: p=0.0276<br>TBI+control vs. TBI+antagomir: p=0.0005<br><br>25d:<br>Sham+control vs. Sham+antagomir: p=0.9992<br>Sham+control vs. TBI+control: p<0.0001<br>Sham+control vs. TBI+antagomir: p=0.5635<br>Sham+antagomir vs. TBI+control: p<0.0001<br>Sham+antagomir vs. TBI+antagomir: p=0.5211<br>TBI+control vs. TBI+antagomir: p=0.0005<br><br>26d:<br>Sham+control vs. Sham+antagomir: p=0.2193<br>Sham+control vs. TBI+control: p<0.0001<br>Sham+control vs. TBI+antagomir: p=0.2345<br>Sham+antagomir vs. TBI+control: p<0.0001<br>Sham+antagomir vs. TBI+antagomir: p=0.009<br>TBI+control vs. TBI+antagomir: p=<0.0001
5H	Sham+control=10<br>Sham+antagomir=10<br>TBI+control=10<br>TBI+antagomir=10	Normal distribution, homogeneity variances	One-way ANOVA, Tukey multiple comparisons test	F (3, 36) = 18.25	One-way ANOVA, p<0.0001<br><br>Tukey's multiple comparisons test:<br><br>Sham+control vs. Sham+antagomir: p=0.9306<br>Sham+control vs. TBI+control: p=<0.0001<br>Sham+control vs. TBI+antagomir: p=0.0383<br>Sham+antagomir vs. TBI+control: p<0.0001<br>Sham+antagomir vs. TBI+antagomir: p=0.1412<br>TBI+control vs. TBI+antagomir: p=0.0024
5I	Sham+control=10<br>Sham+antagomir=10<br>TBI+control=10<br>TBI+antagomir=10	Normal distribution, homogeneity variances	One-way ANOVA, Tukey multiple comparisons test	F (3, 36) = 1.243	One-way ANOVA, p=0.3086<br><br>Tukey's multiple comparisons test:<br><br>Sham+control vs. Sham+antagomir: p=0.3278<br>Sham+control vs. TBI+control: p=0.992<br>Sham+control vs. TBI+antagomir: p=0.6745<br>Sham+antagomir vs. TBI+control: p=0.484<br>Sham+antagomir vs. TBI+antagomir: p=0.9337<br>TBI+control vs. TBI+antagomir: p=0.8319
5K	Sham+control=10<br>Sham+antagomir=10<br>TBI+control=10<br>TBI+antagomir=10	Non-normal distribution	Kruskal-Wallis test. Dunn's multiple comparisons test.	Kruskal-Wallis statistic = 22.56	Kruskal-Wallis test, p<0.0001<br><br>Dunn's multiple comparisons test:<br><br>Sham+control vs. Sham+antagomir: p>0.9999<br>Sham+control vs. TBI+control: p=0.0004<br>Sham+control vs. TBI+antagomir: p>0.9999<br>Sham+antagomir vs. TBI+control: p=0.0003<br>Sham+antagomir vs. TBI+antagomir: p>0.9999<br>TBI+control vs. TBI+antagomir: p=0.0039
5M	TBI+control=10<br>TBI+antagomir=10	Normal distribution	Unpaired t test	t=3.027, df=18	TBI+control vs. TBI+antagomir: p=0.0073
5O	Sham+control=6<br>Sham+antagomir=6<br>TBI+control=6<br>TBI+antagomir=6	Normal distribution	One-way ANOVA, Tukey multiple comparisons test	F (3, 20) = 94.31	One-way ANOVA, p<0.0001<br><br>Tukey's multiple comparisons test:<br><br>Sham+control vs. Sham+antagomir: p=0.9915<br>Sham+control vs. TBI+control: p=<0.0001<br>Sham+control vs. TBI+antagomir: p<0.0001<br>Sham+antagomir vs. TBI+control: p<0.0001<br>Sham+antagomir vs. TBI+antagomir: p<0.0001<br>TBI+control vs. TBI+antagomir: p<0.0001
5P	Sham+control=6<br>Sham+antagomir=6<br>TBI+control=6<br>TBI+antagomir=6	Normal distribution, homogeneity variances	One-way ANOVA, Tukey multiple comparisons test	F (3, 20) = 30.49	One-way ANOVA, p<0.0001<br><br>Tukey's multiple comparisons test:<br><br>Sham+control vs. Sham+antagomir: p=0.9725<br>Sham+control vs. TBI+control: p<0.0001

Figure	n	Data structure	Test used	Statistic	P value
					Sham+control vs. TBI+antagomir: p=0.0007<br>Sham+antagomir vs. TBI+control: p<0.0001<br>Sham+antagomir vs. TBI+antagomir: p=0.0003<br>TBI+control vs. TBI+antagomir: p=0.0356
5Q	Sham+control=6<br>Sham+antagomir=6<br>TBI+control=6<br>TBI+antagomir=6	Normal distribution, homogeneity variances	One-way ANOVA, Tukey multiple comparisons test	F (3, 20) = 1.209	One-way ANOVA, p= 0.3320<br><br>Tukey's multiple comparisons test:<br><br>Sham+control vs. Sham+antagomir: p=0.9807<br>Sham+control vs. TBI+control: p=0.5685<br>Sham+control vs. TBI+antagomir: p=0.9935<br>Sham+antagomir vs. TBI+control: p=0.3552<br>Sham+antagomir vs. TBI+antagomir: p=0.9994<br>TBI+control vs. TBI+antagomir: p=0.4172
6C	Sham+control=6<br>Sham+antagomir=6<br>TBI+control=6<br>TBI+antagomir=6	Normal distribution, homogeneity variances	One-way ANOVA, Tukey multiple comparisons test	F (3, 20) = 85.56	One-way ANOVA, p<0.0001<br><br>Tukey's multiple comparisons test:<br><br>Sham+control vs. Sham+antagomir: p=0.7345<br>Sham+control vs. TBI+control: p<0.0001<br>Sham+control vs. TBI+antagomir: p<0.0001<br>Sham+antagomir vs. TBI+control: p<0.0001<br>Sham+antagomir vs. TBI+antagomir: p<0.0001<br>TBI+control vs. TBI+antagomir: p<0.0001
6D	Sham+control=6<br>Sham+antagomir=6<br>TBI+control=6<br>TBI+antagomir=6	Normal distribution, homogeneity variances	One-way ANOVA, Tukey multiple comparisons test	F (3, 20) = 174.5	One-way ANOVA, p<0.0001<br><br>Tukey's multiple comparisons test:<br><br>Sham+control vs. Sham+antagomir: p=0.9991<br>Sham+control vs. TBI+control: p<0.0001<br>Sham+control vs. TBI+antagomir: p<0.0001<br>Sham+antagomir vs. TBI+control: p<0.0001<br>Sham+antagomir vs. TBI+antagomir: p<0.0001<br>TBI+control vs. TBI+antagomir: p<0.0001
6E	Sham+control=6<br>Sham+antagomir=6<br>TBI+control=6<br>TBI+antagomir=6	Normal distribution, homogeneity variances	One-way ANOVA, Tukey multiple comparisons test	F (3, 20) = 65.39	One-way ANOVA, p<0.0001<br><br>Tukey's multiple comparisons test:<br><br>Sham+control vs. Sham+antagomir: p=0.9992<br>Sham+control vs. TBI+control: p<0.0001<br>Sham+control vs. TBI+antagomir: p=0.0001<br>Sham+antagomir vs. TBI+control: p<0.0001<br>Sham+antagomir vs. TBI+antagomir: p=0.0001<br>TBI+control vs. TBI+antagomir: p<0.0001
6G	Sham+control=6<br>Sham+antagomir=6<br>TBI+control=6<br>TBI+antagomir=6	Normal distribution, homogeneity variances	One-way ANOVA, Tukey multiple comparisons test	F (3, 20) = 59.54	One-way ANOVA, p<0.0001<br><br>Tukey's multiple comparisons test:<br><br>Sham+control vs. Sham+antagomir: p=0.7487<br>Sham+control vs. TBI+control: p<0.0001<br>Sham+control vs. TBI+antagomir: p<0.0001<br>Sham+antagomir vs. TBI+control: p<0.0001<br>Sham+antagomir vs. TBI+antagomir: p<0.0001<br>TBI+control vs. TBI+antagomir: p=0.0029
6H	Sham+control=6<br>Sham+antagomir=6<br>TBI+control=6<br>TBI+antagomir=6	Normal distribution, homogeneity variances	One-way ANOVA, Tukey multiple comparisons test	F (3, 20) = 87.53	One-way ANOVA, p<0.0001<br><br>Tukey's multiple comparisons test:<br><br>Sham+control vs. Sham+antagomir: p=0.8284<br>Sham+control vs. TBI+control: p<0.0001<br>Sham+control vs. TBI+antagomir: p<0.0001<br>Sham+antagomir vs. TBI+control: p<0.0001<br>Sham+antagomir vs. TBI+antagomir: p<0.0001<br>TBI+control vs. TBI+antagomir: p<0.0001
6I	Sham+control=6<br>Sham+antagomir=6<br>TBI+control=6<br>TBI+antagomir=6	Normal distribution	One-way ANOVA, Tukey multiple comparisons test	F (3, 20) = 33.79	One-way ANOVA, p<0.0001<br><br>Tukey's multiple comparisons test:<br><br>Sham+control vs. Sham+antagomir: p=0.9996<br>Sham+control vs. TBI+control: p<0.0001<br>Sham+control vs. TBI+antagomir: p=0.0235<br>Sham+antagomir vs. TBI+control: p<0.0001<br>Sham+antagomir vs. TBI+antagomir: p=0.0292<br>TBI+control vs. TBI+antagomir: p<0.0001
6J	Sham+control=6<br>Sham+antagomir=6<br>TBI+control=6<br>TBI+antagomir=6	Normal distribution, homogeneity variances	One-way ANOVA, Tukey multiple	F (3, 20) = 49.58	One-way ANOVA, p<0.0001<br><br>Tukey's multiple comparisons test:

Figure	n	Data structure	Test used	Statistic	P value
			comparisons test		Sham+control vs. Sham+antagomir: p=0.9989<br>Sham+control vs. TBI+control: p<0.0001<br>Sham+control vs. TBI+antagomir: p<0.0001<br>Sham+antagomir vs. TBI+control: p<0.0001<br>Sham+antagomir vs. TBI+antagomir: p<0.0001<br>TBI+control vs. TBI+antagomir: p=0.001
6K	Sham+control=6<br>Sham+antagomir=6<br>TBI+control=6<br>TBI+antagomir=6	Normal distribution, homogeneity variances	One-way ANOVA, Tukey multiple comparisons test	F (3, 20) = 9.988	One-way ANOVA, p<0.0001<br><br>Tukey's multiple comparisons test:<br><br>Sham+control vs. Sham+antagomir: p=0.9997<br>Sham+control vs. TBI+control: p=0.0006<br>Sham+control vs. TBI+antagomir: p=0.539<br>Sham+antagomir vs. TBI+control: p=0.0008<br>Sham+antagomir vs. TBI+antagomir: p=0.5942<br>TBI+control vs. TBI+antagomir: p=0.0133
6L	Sham+control=6<br>Sham+antagomir=6<br>TBI+control=6<br>TBI+antagomir=6	Normal distribution, homogeneity variances	One-way ANOVA, Tukey multiple comparisons test	F (3, 20) = 78.28	One-way ANOVA, p<0.0001<br><br>Tukey's multiple comparisons test:<br><br>Sham+control vs. Sham+antagomir: p=0.9701<br>Sham+control vs. TBI+control: p<0.0001<br>Sham+control vs. TBI+antagomir: p=0.0285<br>Sham+antagomir vs. TBI+control: p<0.0001<br>Sham+antagomir vs. TBI+antagomir: p=0.071<br>TBI+control vs. TBI+antagomir: p<0.0001
6N	Sham+control=6<br>Sham+antagomir=6<br>TBI+control=6<br>TBI+antagomir=6	Normal distribution, homogeneity variances	One-way ANOVA, Tukey multiple comparisons test	F (3, 20) = 103.7	One-way ANOVA, p<0.0001<br><br>Tukey's multiple comparisons test:<br><br>Sham+control vs. Sham+antagomir: p=0.6795<br>Sham+control vs. TBI+control: p<0.0001<br>Sham+control vs. TBI+antagomir: p<0.0001<br>Sham+antagomir vs. TBI+control: p<0.0001<br>Sham+antagomir vs. TBI+antagomir: p<0.0001<br>TBI+control vs. TBI+antagomir: p=0.0002
6O	Sham+control=6<br>Sham+antagomir=6<br>TBI+control=6<br>TBI+antagomir=6	Normal distribution, homogeneity variances	One-way ANOVA, Tukey multiple comparisons test	F (3, 20) = 75.36	One-way ANOVA, p<0.0001<br><br>Tukey's multiple comparisons test:<br><br>Sham+control vs. Sham+antagomir: p=0.915<br>Sham+control vs. TBI+control: p<0.0001<br>Sham+control vs. TBI+antagomir: p<0.0001<br>Sham+antagomir vs. TBI+control: p<0.0001<br>Sham+antagomir vs. TBI+antagomir: p0.0001<br>TBI+control vs. TBI+antagomir: p<0.0001
6P	Sham+control=6<br>Sham+antagomir=6<br>TBI+control=6<br>TBI+antagomir=6	Normal distribution, homogeneity variances	One-way ANOVA, Tukey multiple comparisons test	F (3, 20) = 3.595	One-way ANOVA, p=0.0316<br><br>Tukey's multiple comparisons test:<br><br>Sham+control vs. Sham+antagomir: p=0.9999<br>Sham+control vs. TBI+control: p=0.0875<br>Sham+control vs. TBI+antagomir: p=0.1659<br>Sham+antagomir vs. TBI+control: p=0.1001<br>Sham+antagomir vs. TBI+antagomir: p=0.1872<br>TBI+control vs. TBI+antagomir: p=0.9852
7D	Sham+control=6<br>Sham+antagomir=6<br>TBI+control=6<br>TBI+antagomir=6	Normal distribution, homogeneity variances	One-way ANOVA, Tukey multiple comparisons test	F (3, 20) = 137.1	One-way ANOVA, p<0.0001<br><br>Tukey's multiple comparisons test:<br><br>Sham+control vs. Sham+antagomir: p>0.9999<br>Sham+control vs. TBI+control: p<0.0001<br>Sham+control vs. TBI+antagomir: p<0.0001<br>Sham+antagomir vs. TBI+control: p<0.0001<br>Sham+antagomir vs. TBI+antagomir: p<0.0001<br>TBI+control vs. TBI+antagomir: p<0.0001
7E	Sham+control=6<br>Sham+antagomir=6<br>TBI+control=6<br>TBI+antagomir=6	Normal distribution, homogeneity variances	One-way ANOVA, Tukey multiple comparisons test	F (3, 20) = 161.1	One-way ANOVA, p<0.0001<br><br>Tukey's multiple comparisons test:<br><br>Sham+control vs. Sham+antagomir: p=0.9315<br>Sham+control vs. TBI+control: p<0.0001<br>Sham+control vs. TBI+antagomir: p<0.0001<br>Sham+antagomir vs. TBI+control: p<0.0001<br>Sham+antagomir vs. TBI+antagomir: p<0.0001<br>TBI+control vs. TBI+antagomir: p<0.0001
7F	Sham+control=6<br>Sham+antagomir=6	Normal distribution,	One-way ANOVA,	F (3, 20) = 125.3	One-way ANOVA, p<0.0001

Figure	n	Data structure	Test used	Statistic	P value
	TBI+control=6<br>TBI+antagomir=6	homogeneity variances	Tukey multiple comparisons test		Tukey's multiple comparisons test:<br><br>Sham+control vs. Sham+antagomir: p=0.999<br>Sham+control vs. TBI+control: p<0.0001<br>Sham+control vs. TBI+antagomir: p<0.0001<br>Sham+antagomir vs. TBI+control: p<0.0001<br>Sham+antagomir vs. TBI+antagomir: p<0.0001<br>TBI+control vs. TBI+antagomir: p<0.0001
7I	Sham+control=6<br>Sham+antagomir=6<br>TBI+control=6<br>TBI+antagomir=6	Normal distribution, homogeneity variances	One-way ANOVA, Tukey multiple comparisons test	F (3, 20) = 408.1	One-way ANOVA, p<0.0001<br><br>Tukey's multiple comparisons test:<br><br>Sham+control vs. Sham+antagomir: p>0.9999<br>Sham+control vs. TBI+control: p<0.0001<br>Sham+control vs. TBI+antagomir: p<0.0001<br>Sham+antagomir vs. TBI+control: p<0.0001<br>Sham+antagomir vs. TBI+antagomir: p<0.0001<br>TBI+control vs. TBI+antagomir: p<0.0001
7J	Sham+control=6<br>Sham+antagomir=6<br>TBI+control=6<br>TBI+antagomir=6	Normal distribution, homogeneity variances	One-way ANOVA, Tukey multiple comparisons test	F (3, 20) = 173.9	One-way ANOVA, p<0.0001<br><br>Tukey's multiple comparisons test:<br><br>Sham+control vs. Sham+antagomir: p>0.9999<br>Sham+control vs. TBI+control: p<0.0001<br>Sham+control vs. TBI+antagomir: p<0.0001<br>Sham+antagomir vs. TBI+control: p<0.0001<br>Sham+antagomir vs. TBI+antagomir: p<0.0001<br>TBI+control vs. TBI+antagomir: p<0.0001
7M	Sham+control=6<br>Sham+antagomir=6<br>TBI+control=6<br>TBI+antagomir=6	Normal distribution, homogeneity variances	One-way ANOVA, Tukey multiple comparisons test	F (3, 20) = 303.9	One-way ANOVA, p<0.0001<br><br>Tukey's multiple comparisons test:<br><br>Sham+control vs. Sham+antagomir: p>0.9999<br>Sham+control vs. TBI+control: p<0.0001<br>Sham+control vs. TBI+antagomir: p<0.0001<br>Sham+antagomir vs. TBI+control: p<0.0001<br>Sham+antagomir vs. TBI+antagomir: p<0.0001<br>TBI+control vs. TBI+antagomir: p<0.0001
7N	Sham+control=6<br>Sham+antagomir=6<br>TBI+control=6<br>TBI+antagomir=6	Normal distribution, homogeneity variances	One-way ANOVA, Tukey multiple comparisons test	F (3, 20) = 545.1	One-way ANOVA, p<0.0001<br><br>Tukey's multiple comparisons test:<br><br>Sham+control vs. Sham+antagomir: p>0.9999<br>Sham+control vs. TBI+control: p<0.0001<br>Sham+control vs. TBI+antagomir: p<0.0001<br>Sham+antagomir vs. TBI+control: p<0.0001<br>Sham+antagomir vs. TBI+antagomir: p<0.0001<br>TBI+control vs. TBI+antagomir: p<0.0001
8C: BLC	Sham+WT=4<br>Sham+KO=4<br>TBI+WT=4<br>TBI+KO=4	Non-normal distribution	Kruskal-Wallis test, Dunn's multiple comparisons test	Kruskal-Wallis statistic=10.92	Kruskal-Wallis test, p=0.0017<br><br>Dunn's multiple comparisons test:<br><br>Sham+WT vs. Sham+KO: p>0.9999<br>Sham+WT vs. TBI+WT: p=0.0856<br>Sham+WT vs. TBI+KO: p>0.9999<br>Sham+KO vs. TBI+WT: p=0.0109<br>Sham+KO vs. TBI+KO: p=0.7133<br>TBI+WT vs. TBI+KO: p=0.7133
8C: CD30L	Sham+WT=4<br>Sham+KO=4<br>TBI+WT=4<br>TBI+KO=4	Normal distribution, homogeneity variances	One-way ANOVA, Tukey multiple comparisons test	F (3, 12) = 19.71	One-way ANOVA, p<0.0001<br><br>Tukey's multiple comparisons test:<br><br>Sham+WT vs. Sham+KO: p=0.9636<br>Sham+WT vs. TBI+WT: p=0.0001<br>Sham+WT vs. TBI+KO: p=0.6262<br>Sham+KO vs. TBI+WT: p=0.0002<br>Sham+KO vs. TBI+KO: p=0.8759<br>TBI+WT vs. TBI+KO: p=0.0006
8C: Eotaxin	Sham+WT=4<br>Sham+KO=4<br>TBI+WT=4<br>TBI+KO=4	Normal distribution, homogeneity variances	One-way ANOVA, Tukey multiple comparisons test	F (3, 12) = 14.39	One-way ANOVA, p=0.0003<br><br>Tukey's multiple comparisons test:<br><br>Sham+WT vs. Sham+KO: p=0.9104<br>Sham+WT vs. TBI+WT: p=0.0009<br>Sham+WT vs. TBI+KO: p=0.2981<br>Sham+KO vs. TBI+WT: p=0.0003<br>Sham+KO vs. TBI+KO: p=0.1087

Figure	n	Data structure	Test used	Statistic	P value
					TBI+WT vs. TBI+KO: p=0.0208
8C:<br>Eotaxin-2	Sham+WT=4<br>Sham+KO=4<br>TBI+WT=4<br>TBI+KO=4	Normal distribution, homogeneity variances	One-way ANOVA, Tukey multiple comparisons test	F (3, 12) = 38.59	One-way ANOVA, p<0.0001<br><br>Tukey's multiple comparisons test:<br><br>Sham+WT vs. Sham+KO: p=0.9985<br>Sham+WT vs. TBI+WT: p<0.0001<br>Sham+WT vs. TBI+KO: p=0.1185<br>Sham+KO vs. TBI+WT: p<0.0001<br>Sham+KO vs. TBI+KO: p=0.1527<br>TBI+WT vs. TBI+KO: p=<0.0001
8C:<br>Fas ligand	Sham+WT=4<br>Sham+KO=4<br>TBI+WT=4<br>TBI+KO=4	Non-normal distribution	Kruskal-Wallis test, Dunn's multiple comparisons test	Kruskal-Wallis statistic=8.890	Kruskal-Wallis test, p=0.0144<br><br>Dunn's multiple comparisons test:<br><br>Sham+WT vs. Sham+KO: p>0.9999<br>Sham+WT vs. TBI+WT: p=0.1876<br>Sham+WT vs. TBI+KO: p>0.9999<br>Sham+KO vs. TBI+WT: p=0.036<br>Sham+KO vs. TBI+KO: p>0.9999<br>TBI+WT vs. TBI+KO: p=0.1554
8C:<br>Fractalkine	Sham+WT=4<br>Sham+KO=4<br>TBI+WT=4<br>TBI+KO=4	Normal distribution, homogeneity variances	One-way ANOVA, Tukey multiple comparisons test	F (3, 12) = 2.470	One-way ANOVA, p=0.1119<br><br>Tukey's multiple comparisons test:<br><br>Sham+WT vs. Sham+KO: p=0.9998<br>Sham+WT vs. TBI+WT: p=0.2293<br>Sham+WT vs. TBI+KO: p=0.9624<br>Sham+KO vs. TBI+WT: p=0.2575<br>Sham+KO vs. TBI+KO: p=0.9425<br>TBI+WT vs. TBI+KO: p=0.1082
8C:<br>G-CSF	Sham+WT=4<br>Sham+KO=4<br>TBI+WT=4<br>TBI+KO=4	Normal distribution, heterogeneous variances	Welch's ANOVA test, Dunnett's T3 multiple comparisons test	W (3.000, 7.152) = 199.8	Welch's ANOVA test, p<0.0001<br><br>Dunnett's T3 multiple comparisons test:<br><br>Sham+WT vs. Sham+KO: p=0.1647<br>Sham+WT vs. TBI+WT: p<0.0001<br>Sham+WT vs. TBI+KO: p=0.5025<br>Sham+KO vs. TBI+WT: p<0.0001<br>Sham+KO vs. TBI+KO: p=0.1542<br>TBI+WT vs. TBI+KO: p=0.0049
8C:<br>GM-CSF	Sham+WT=4<br>Sham+KO=4<br>TBI+WT=4<br>TBI+KO=4	Normal distribution, homogeneity variances	One-way ANOVA, Tukey multiple comparisons test	F (3, 12) = 23.27	One-way ANOVA, p<0.0001<br><br>Tukey's multiple comparisons test:<br><br>Sham+WT vs. Sham+KO: p=0.7327<br>Sham+WT vs. TBI+WT: p=0.0001<br>Sham+WT vs. TBI+KO: p=0.5364<br>Sham+KO vs. TBI+WT: p<0.0001<br>Sham+KO vs. TBI+KO: p=0.1277<br>TBI+WT vs. TBI+KO: p=0.001
8C:<br>IFN-γ	Sham+WT=4<br>Sham+KO=4<br>TBI+WT=4<br>TBI+KO=4	Normal distribution, homogeneity variances	One-way ANOVA, Tukey multiple comparisons test	F (3, 12) = 7.198	One-way ANOVA, p=0.0051<br><br>Tukey's multiple comparisons test:<br><br>Sham+WT vs. Sham+KO: p=0.9986<br>Sham+WT vs. TBI+WT: p=0.0668<br>Sham+WT vs. TBI+KO: p=0.3341<br>Sham+KO vs. TBI+WT: p=0.0511<br>Sham+KO vs. TBI+KO: p=0.4087<br>TBI+WT vs. TBI+KO: p=0.0031
8C:<br>IL1-α	Sham+WT=4<br>Sham+KO=4<br>TBI+WT=4<br>TBI+KO=4	Non-normal distribution	Kruskal-Wallis test, Dunn's multiple comparisons test	Kruskal-Wallis statistic=11.93	Kruskal-Wallis test, p=0.0003<br><br>Dunn's multiple comparisons test:<br><br>Sham+WT vs. Sham+KO: p>0.9999<br>Sham+WT vs. TBI+WT: p=0.0227<br>Sham+WT vs. TBI+KO: p=0.0561<br>Sham+KO vs. TBI+WT: p=0.1876<br>Sham+KO vs. TBI+KO: p=0.3803<br>TBI+WT vs. TBI+KO: p>0.9999

Figure	n	Data structure	Test used	Statistic	P value
8D:<br>IL1-β	Sham+WT=4<br>Sham+KO=4<br>TBI+WT=4<br>TBI+KO=4	Normal distribution, homogeneity variances	One-way ANOVA, Tukey multiple comparisons test	F (3, 12) = 10.51	One-way ANOVA, p=0.0011<br><br>Tukey's multiple comparisons test:<br><br>Sham+WT vs. Sham+KO: p=0.9972<br>Sham+WT vs. TBI+WT: p=0.002<br>Sham+WT vs. TBI+KO: p=0.9005<br>Sham+KO vs. TBI+WT: p=0.0027<br>Sham+KO vs. TBI+KO: p=0.9599<br>TBI+WT vs. TBI+KO: p=0.0063
8D:<br>IL2	Sham+WT=4<br>Sham+KO=4<br>TBI+WT=4<br>TBI+KO=4	Normal distribution, homogeneity variances	One-way ANOVA, Tukey multiple comparisons test	F (3, 12) = 10.21	One-way ANOVA, p=0.0013<br><br>Tukey's multiple comparisons test:<br><br>Sham+WT vs. Sham+KO: p=0.9814<br>Sham+WT vs. TBI+WT: p=0.0091<br>Sham+WT vs. TBI+KO: p=0.7034<br>Sham+KO vs. TBI+WT: p=0.0048<br>Sham+KO vs. TBI+KO: p=0.8895<br>TBI+WT vs. TBI+KO: p=0.0014
8D:<br>IL3	Sham+WT=4<br>Sham+KO=4<br>TBI+WT=4<br>TBI+KO=4	Normal distribution, homogeneity variances	One-way ANOVA, Tukey multiple comparisons test	F (3, 12) = 24.45	One-way ANOVA, p<0.0001<br><br>Tukey's multiple comparisons test:<br><br>Sham+WT vs. Sham+KO: p=0.9555<br>Sham+WT vs. TBI+WT: p<0.0001<br>Sham+WT vs. TBI+KO: p=0.472<br>Sham+KO vs. TBI+WT: p<0.0001<br>Sham+KO vs. TBI+KO: p=0.7618<br>TBI+WT vs. TBI+KO: p=0.0003
8D:<br>IL4	Sham+WT=4<br>Sham+KO=4<br>TBI+WT=4<br>TBI+KO=4	Normal distribution, homogeneity variances	One-way ANOVA, Tukey multiple comparisons test	F (3, 12) = 0.477	One-way ANOVA, p=.07040<br><br>Tukey's multiple comparisons test:<br><br>Sham+WT vs. Sham+KO: p=0.7105<br>Sham+WT vs. TBI+WT: p=0.9997<br>Sham+WT vs. TBI+KO: p=0.9885<br>Sham+KO vs. TBI+WT: p=0.7598<br>Sham+KO vs. TBI+KO: p=0.8712<br>TBI+WT vs. TBI+KO: p=0.9956
8D:<br>IL6	Sham+WT=4<br>Sham+KO=4<br>TBI+WT=4<br>TBI+KO=4	Normal distribution, homogeneity variances	One-way ANOVA, Tukey multiple comparisons test	F (3, 12) = 16.63	One-way ANOVA, p=0.0001<br><br>Tukey's multiple comparisons test:<br><br>Sham+WT vs. Sham+KO: p=0.1166<br>Sham+WT vs. TBI+WT: p=0.004<br>Sham+WT vs. TBI+KO: p=0.4598<br>Sham+KO vs. TBI+WT: p<0.0001<br>Sham+KO vs. TBI+KO: p=0.0085<br>TBI+WT vs. TBI+KO: p=0.0552
8D:<br>IL9	Sham+WT=4<br>Sham+KO=4<br>TBI+WT=4<br>TBI+KO=4	Normal distribution, homogeneity variances	One-way ANOVA, Tukey multiple comparisons test	F (3, 12) = 41.07	One-way ANOVA, p<0.0001<br><br>Tukey's multiple comparisons test:<br><br>Sham+WT vs. Sham+KO: p=0.816<br>Sham+WT vs. TBI+WT: p<0.0001<br>Sham+WT vs. TBI+KO: p=0.0071<br>Sham+KO vs. TBI+WT: p<0.0001<br>Sham+KO vs. TBI+KO: p=0.0016<br>TBI+WT vs. TBI+KO: p=0.0016
8D:<br>IL10	Sham+WT=4<br>Sham+KO=4<br>TBI+WT=4<br>TBI+KO=4	Normal distribution, homogeneity variances	One-way ANOVA, Tukey multiple comparisons test	F (3, 12) = 19.95	One-way ANOVA, p<0.0001<br><br>Tukey's multiple comparisons test:<br><br>Sham+WT vs. Sham+KO: p=0.2647<br>Sham+WT vs. TBI+WT: p=0.0007<br>Sham+WT vs. TBI+KO: p=0.7422<br>Sham+KO vs. TBI+WT: p<0.0001<br>Sham+KO vs. TBI+KO: p=0.0513<br>TBI+WT vs. TBI+KO: p=0.0035

Figure	n	Data structure	Test used	Statistic	P value
8D:<br>IL12-p40/p70	Sham+WT=4<br>Sham+KO=4<br>TBI+WT=4<br>TBI+KO=4	Normal distribution, homogeneity variances	One-way ANOVA, Tukey multiple comparisons test	F (3, 12) = 16.88	One-way ANOVA, p=0.0001<br><br>Tukey's multiple comparisons test:<br><br>Sham+WT vs. Sham+KO: p=0.2123<br>Sham+WT vs. TBI+WT: p=0.0024<br>Sham+WT vs. TBI+KO: p=0.9917<br>Sham+KO vs. TBI+WT: p<0.0001<br>Sham+KO vs. TBI+KO: p=0.3186<br>TBI+WT vs. TBI+KO: p=0.0015
8D:<br>IL12-p70	Sham+WT=4<br>Sham+KO=4<br>TBI+WT=4<br>TBI+KO=4	Normal distribution, homogeneity variances	One-way ANOVA, Tukey multiple comparisons test	F (3, 12) = 1.923	One-way ANOVA, p=0.1797<br><br>Tukey's multiple comparisons test:<br><br>Sham+WT vs. Sham+KO: p=0.9765<br>Sham+WT vs. TBI+WT: p=0.3465<br>Sham+WT vs. TBI+KO: p=0.7169<br>Sham+KO vs. TBI+WT: p=0.1941<br>Sham+KO vs. TBI+KO: p=0.4843<br>TBI+WT vs. TBI+KO: p=0.904
8D:<br>IL13	Sham+WT=4<br>Sham+KO=4<br>TBI+WT=4<br>TBI+KO=4	Normal distribution, homogeneity variances	One-way ANOVA, Tukey multiple comparisons test	F (3, 12) = 14.29	One-way ANOVA, p=0.0003<br><br>Tukey's multiple comparisons test:<br><br>Sham+WT vs. Sham+KO: p=0.9745<br>Sham+WT vs. TBI+WT: p=0.0004<br>Sham+WT vs. TBI+KO: p=0.1627<br>Sham+KO vs. TBI+WT: p=0.0007<br>Sham+KO vs. TBI+KO: p=0.302<br>TBI+WT vs. TBI+KO: p=0.0164
8E:<br>IL17	Sham+WT=4<br>Sham+KO=4<br>TBI+WT=4<br>TBI+KO=4	Non-normal distribution	Kruskal-Wallis test, Dunn's multiple comparisons test	Kruskal-Wallis statistic=10.68	Kruskal-Wallis test, p=0.0023<br><br>Dunn's multiple comparisons test:<br><br>Sham+WT vs. Sham+KO: p=0.8249<br>Sham+WT vs. TBI+WT: p=0.6139<br>Sham+WT vs. TBI+KO: p>0.9999<br>Sham+KO vs. TBI+WT: p=0.0109<br>Sham+KO vs. TBI+KO: p>0.9999<br>TBI+WT vs. TBI+KO: p=0.1049
8E:<br>I-TAC	Sham+WT=4<br>Sham+KO=4<br>TBI+WT=4<br>TBI+KO=4	Non-normal distribution	Kruskal-Wallis test, Dunn's multiple comparisons test	Kruskal-Wallis statistic=10.15	Kruskal-Wallis test, p=0.0041<br><br>Dunn's multiple comparisons test:<br><br>Sham+WT vs. Sham+KO: p>0.9999<br>Sham+WT vs. TBI+WT: p>0.9999<br>Sham+WT vs. TBI+KO: p0.6139<br>Sham+KO vs. TBI+WT: p0.0694<br>Sham+KO vs. TBI+KO: p>0.9999<br>TBI+WT vs. TBI+KO: p=0.0286
8E:<br>KC	Sham+WT=4<br>Sham+KO=4<br>TBI+WT=4<br>TBI+KO=4	Normal distribution, homogeneity variances	One-way ANOVA, Tukey multiple comparisons test	F (3, 12) = 8.836	One-way ANOVA, p=0.0023<br><br>Tukey's multiple comparisons test:<br><br>Sham+WT vs. Sham+KO: p=0.38<br>Sham+WT vs. TBI+WT: p=0.0284<br>Sham+WT vs. TBI+KO: p=0.9927<br>Sham+KO vs. TBI+WT: p=0.0016<br>Sham+KO vs. TBI+KO: p=0.5235<br>TBI+WT vs. TBI+KO: p=0.0177
8E:<br>Leptin	Sham+WT=4<br>Sham+KO=4<br>TBI+WT=4<br>TBI+KO=4	Normal distribution, homogeneity variances	One-way ANOVA, Tukey multiple comparisons test	F (3, 12) = 13.21	One-way ANOVA, p=0.0004<br><br>Tukey's multiple comparisons test:<br><br>Sham+WT vs. Sham+KO: p=0.3755<br>Sham+WT vs. TBI+WT: p=0.0042<br>Sham+WT vs. TBI+KO: p=0.971<br>Sham+KO vs. TBI+WT: p=0.0003<br>Sham+KO vs. TBI+KO: p=0.2033<br>TBI+WT vs. TBI+KO: p=0.0089

Figure	n	Data structure	Test used	Statistic	P value
8E:<br>LIX	Sham+WT=4<br>Sham+KO=4<br>TBI+WT=4<br>TBI+KO=4	Normal distribution, homogeneity variances	One-way ANOVA, Tukey multiple comparisons test	F (3, 12) = 11.50	One-way ANOVA, p=0.0008<br><br>Tukey's multiple comparisons test:<br><br>Sham+WT vs. Sham+KO: p=0.7412<br>Sham+WT vs. TBI+WT: p=0.004<br>Sham+WT vs. TBI+KO: p=0.0023<br>Sham+KO vs. TBI+WT: p=0.0235<br>Sham+KO vs. TBI+KO: p=0.0129<br>TBI+WT vs. TBI+KO: p=0.9858
8E:<br>Lymphotactin	Sham+WT=4<br>Sham+KO=4<br>TBI+WT=4<br>TBI+KO=4	Normal distribution, homogeneity variances	One-way ANOVA, Tukey multiple comparisons test	F (3, 12) = 7.926	One-way ANOVA, p=0.0035<br><br>Tukey's multiple comparisons test:<br><br>Sham+WT vs. Sham+KO: p=0.4125<br>Sham+WT vs. TBI+WT: p=0.051<br>Sham+WT vs. TBI+KO: p=0.3626<br>Sham+KO vs. TBI+WT: p=0.0032<br>Sham+KO vs. TBI+KO: p=0.0276<br>TBI+WT vs. TBI+KO: p=0.6082
8E:<br>MCP-1	Sham+WT=4<br>Sham+KO=4<br>TBI+WT=4<br>TBI+KO=4	Non-normal distribution	Kruskal-Wallis test, Dunn's multiple comparisons test	Kruskal-Wallis statistic=12.90	Kruskal-Wallis test, p<0.0001<br><br>Dunn's multiple comparisons test:<br><br>Sham+WT vs. Sham+KO: p>0.9999<br>Sham+WT vs. TBI+WT: p=0.0084<br>Sham+WT vs. TBI+KO: p=0.2697<br>Sham+KO vs. TBI+WT: p=0.036<br>Sham+KO vs. TBI+KO: p=0.7133<br>TBI+WT vs. TBI+KO: p>0.9999
8E:<br>M-CSF	Sham+WT=4<br>Sham+KO=4<br>TBI+WT=4<br>TBI+KO=4	Normal distribution, homogeneity variances	One-way ANOVA, Tukey multiple comparisons test	F (3, 12) = 5.607	One-way ANOVA, p=0.0122<br><br>Tukey's multiple comparisons test:<br><br>Sham+WT vs. Sham+KO: p=0.9273<br>Sham+WT vs. TBI+WT: p=0.0301<br>Sham+WT vs. TBI+KO: p=0.0414<br>Sham+KO vs. TBI+WT: p=0.0858<br>Sham+KO vs. TBI+KO: p=0.1163<br>TBI+WT vs. TBI+KO: p=0.9977
8E:<br>MIG	Sham+WT=4<br>Sham+KO=4<br>TBI+WT=4<br>TBI+KO=4	Normal distribution, homogeneity variances	One-way ANOVA, Tukey multiple comparisons test	F (3, 12) = 7.466	One-way ANOVA, p=0.0044<br><br>Tukey's multiple comparisons test:<br><br>Sham+WT vs. Sham+KO: p=0.3492<br>Sham+WT vs. TBI+WT: p=0.1042<br>Sham+WT vs. TBI+KO: p=0.5475<br>Sham+KO vs. TBI+WT: p=0.0052<br>Sham+KO vs. TBI+KO: p=0.9807<br>TBI+WT vs. TBI+KO: p=0.01
8E:<br>MIP-1-α	Sham+WT=4<br>Sham+KO=4<br>TBI+WT=4<br>TBI+KO=4	Normal distribution, homogeneity variances	One-way ANOVA, Tukey multiple comparisons test	F (3, 12) = 79.74	One-way ANOVA, p<0.0001<br><br>Tukey's multiple comparisons test:<br><br>Sham+WT vs. Sham+KO: p=0.0129<br>Sham+WT vs. TBI+WT: p<0.0001<br>Sham+WT vs. TBI+KO: p=0.9375<br>Sham+KO vs. TBI+WT: p<0.0001<br>Sham+KO vs. TBI+KO: p=0.0354<br>TBI+WT vs. TBI+KO: p<0.0001
8F:<br>MIP-1-γ	Sham+WT=4<br>Sham+KO=4<br>TBI+WT=4<br>TBI+KO=4	Normal distribution, homogeneity variances	One-way ANOVA, Tukey multiple comparisons test	F (3, 12) = 112.1	One-way ANOVA, p<0.0001<br><br>Tukey's multiple comparisons test:<br><br>Sham+WT vs. Sham+KO: p=0.3092<br>Sham+WT vs. TBI+WT: p<0.0001<br>Sham+WT vs. TBI+KO: p=0.0017<br>Sham+KO vs. TBI+WT: p<0.0001<br>Sham+KO vs. TBI+KO: p=0.0001<br>TBI+WT vs. TBI+KO: p<0.0001
8F:<br>RANTES	Sham+WT=4<br>Sham+KO=4	Normal distribution,	One-way ANOVA,	F (3, 12) = 6.697	One-way ANOVA, p=0.0066

Figure	n	Data structure	Test used	Statistic	P value
	TBI+WT=4<br>TBI+KO=4	homogeneity variances	Tukey multiple comparisons test		Tukey's multiple comparisons test:<br><br>Sham+WT vs. Sham+KO: p=0.9341<br>Sham+WT vs. TBI+WT: p=0.019<br>Sham+WT vs. TBI+KO: p=0.5016<br>Sham+KO vs. TBI+WT: p=0.0068<br>Sham+KO vs. TBI+KO: p=0.2327<br>TBI+WT vs. TBI+KO: p=0.2121
8F:<br>SDF-1	Sham+WT=4<br>Sham+KO=4<br>TBI+WT=4<br>TBI+KO=4	Normal distribution, homogeneity variances	One-way ANOVA, Tukey multiple comparisons test	F (3, 12) = 11.50	One-way ANOVA, p=0.0008<br><br>Tukey's multiple comparisons test:<br><br>Sham+WT vs. Sham+KO: p=0.324<br>Sham+WT vs. TBI+WT: p=0.0092<br>Sham+WT vs. TBI+KO: p=0.9811<br>Sham+KO vs. TBI+WT: p=0.0005<br>Sham+KO vs. TBI+KO: p=0.188<br>TBI+WT vs. TBI+KO: p=0.0178
8F:<br>TCA-3	Sham+WT=4<br>Sham+KO=4<br>TBI+WT=4<br>TBI+KO=4	Normal distribution, homogeneity variances	One-way ANOVA, Tukey multiple comparisons test	F (3, 12) = 5.317	One-way ANOVA, p=0.0146<br><br>Tukey's multiple comparisons test:<br><br>Sham+WT vs. Sham+KO: p=0.6914<br>Sham+WT vs. TBI+WT: p=0.0708<br>Sham+WT vs. TBI+KO: p=0.0158<br>Sham+KO vs. TBI+WT: p=0.3856<br>Sham+KO vs. TBI+KO: p=0.1067<br>TBI+WT vs. TBI+KO: p=0.8233
8F:<br>TECK	Sham+WT=4<br>Sham+KO=4<br>TBI+WT=4<br>TBI+KO=4	Normal distribution, homogeneity variances	One-way ANOVA, Tukey multiple comparisons test	F (3, 12) = 8.113	One-way ANOVA, p=0.0032<br><br>Tukey's multiple comparisons test:<br><br>Sham+WT vs. Sham+KO: p=0.7941<br>Sham+WT vs. TBI+WT: p=0.0131<br>Sham+WT vs. TBI+KO: p=0.8501<br>Sham+KO vs. TBI+WT: p=0.0027<br>Sham+KO vs. TBI+KO: p=0.3521<br>TBI+WT vs. TBI+KO: p=0.0537
8F:<br>TIMP1	Sham+WT=4<br>Sham+KO=4<br>TBI+WT=4<br>TBI+KO=4	Normal distribution, homogeneity variances	One-way ANOVA, Tukey multiple comparisons test	F (3, 12) = 2.161	One-way ANOVA, p=0.1457<br><br>Tukey's multiple comparisons test:<br><br>Sham+WT vs. Sham+KO: p=0.1119<br>Sham+WT vs. TBI+WT: p=0.8533<br>Sham+WT vs. TBI+KO: p=0.7329<br>Sham+KO vs. TBI+WT: p=0.3687<br>Sham+KO vs. TBI+KO: p=0.4907<br>TBI+WT vs. TBI+KO: p=0.9951
8F:<br>TIMP2	Sham+WT=4<br>Sham+KO=4<br>TBI+WT=4<br>TBI+KO=4	Normal distribution, homogeneity variances	One-way ANOVA, Tukey multiple comparisons test	F (3, 12) = 4.244	One-way ANOVA, p=0.0292<br><br>Tukey's multiple comparisons test:<br><br>Sham+WT vs. Sham+KO: p=0.2421<br>Sham+WT vs. TBI+WT: p=0.5645<br>Sham+WT vs. TBI+KO: p=0.618<br>Sham+KO vs. TBI+WT: p=0.0269<br>Sham+KO vs. TBI+KO: p=0.8697<br>TBI+WT vs. TBI+KO: p=0.0998
8F:<br>TNF-α	Sham+WT=4<br>Sham+KO=4<br>TBI+WT=4<br>TBI+KO=4	Normal distribution, homogeneity variances	One-way ANOVA, Tukey multiple comparisons test	F (3, 12) = 6.464	One-way ANOVA, p=0.0075<br><br>Tukey's multiple comparisons test:<br><br>Sham+WT vs. Sham+KO: p=0.5246<br>Sham+WT vs. TBI+WT: p=0.0614<br>Sham+WT vs. TBI+KO: p=0.9979<br>Sham+KO vs. TBI+WT: p=0.0054<br>Sham+KO vs. TBI+KO: p=0.6277<br>TBI+WT vs. TBI+KO: p=0.0452
8F:<br>sTNF RI	Sham+WT=4<br>Sham+KO=4<br>TBI+WT=4	Normal distribution, homogeneity	One-way ANOVA, Tukey	F (3, 12) = 10.38	One-way ANOVA, p=0.0012<br><br>Tukey's multiple comparisons test:

Figure	n	Data structure	Test used	Statistic	P value
	TBI+KO=4	variances	multiple comparisons test		Sham+WT vs. Sham+KO: p=0.9965<br>Sham+WT vs. TBI+WT: p=0.0024<br>Sham+WT vs. TBI+KO: p=0.0477<br>Sham+KO vs. TBI+WT: p=0.0035<br>Sham+KO vs. TBI+KO: p=0.0685<br>TBI+WT vs. TBI+KO: p=0.3566
8F:<br>sTNF RII	Sham+WT=4<br>Sham+KO=4<br>TBI+WT=4<br>TBI+KO=4	Normal distribution, homogeneity variances	One-way ANOVA, Tukey multiple comparisons test	F (3, 12) = 27.06	One-way ANOVA, p<0.0001<br><br>Tukey's multiple comparisons test:<br><br>Sham+WT vs. Sham+KO: p=0.9968<br>Sham+WT vs. TBI+WT: p<0.0001<br>Sham+WT vs. TBI+KO: p=0.209<br>Sham+KO vs. TBI+WT: p<0.0001<br>Sham+KO vs. TBI+KO: p=0.2815<br>TBI+WT vs. TBI+KO: p=0.0004
8I:<br>BLC	Sham+control=4<br>Sham+antagomir=4<br>TBI+control=4<br>TBI+antagomir=4	Normal distribution, homogeneity variances	One-way ANOVA, Tukey multiple comparisons test	F (3, 12) = 13.93	One-way ANOVA, p=0.0003<br><br>Tukey's multiple comparisons test:<br><br>Sham+control vs. Sham+antagomir: p=0.9928<br>Sham+control vs. TBI+control T: p=0.0033<br>Sham+control vs. TBI+antagomir: p=0.4738<br>Sham+antagomir vs. TBI+control: p=0.0021<br>Sham+antagomir vs. TBI+antagomir: p=0.6279<br>TBI+control vs. TBI+antagomir: p=0.0003
8I:<br>CD30L	Sham+control=4<br>Sham+antagomir=4<br>TBI+control=4<br>TBI+antagomir=4	Normal distribution, homogeneity variances	One-way ANOVA, Tukey multiple comparisons test	F (3, 12) = 7.541	One-way ANOVA, p=0.0043<br><br>Tukey's multiple comparisons test:<br><br>Sham+control vs. Sham+antagomir: p=0.991<br>Sham+control vs. TBI+control: p=0.3725<br>Sham+control vs. TBI+antagomir: p=0.0469<br>Sham+antagomir vs. TBI+control: p=0.2501<br>Sham+antagomir vs. TBI+antagomir: p=0.077<br>TBI+control vs. TBI+antagomir: p=0.0025
8I:<br>Eotaxin	Sham+control=4<br>Sham+antagomir=4<br>TBI+control=4<br>TBI+antagomir=4	Normal distribution, homogeneity variances	One-way ANOVA, Tukey multiple comparisons test	F (3, 12) = 3.360	One-way ANOVA, p=0.0551<br><br>Tukey's multiple comparisons test:<br><br>Sham+control vs. Sham+antagomir: p=0.9896<br>Sham+control vs. TBI+control: p=0.1015<br>Sham+control vs. TBI+antagomir: p=0.9842<br>Sham+antagomir vs. TBI+control: p=0.0606<br>Sham+antagomir vs. TBI+antagomir: p=0.9105<br>TBI+control vs. TBI+antagomir: p=0.1788
8I: Eotaxin-2	Sham+control=4<br>Sham+antagomir=4<br>TBI+control=4<br>TBI+antagomir=4	Normal distribution, homogeneity variances	One-way ANOVA, Tukey multiple comparisons test	F (3, 12) = 9.033	One-way ANOVA, p=0.0021<br><br>Tukey's multiple comparisons test:<br><br>Sham+control vs. Sham+antagomir: p=0.7478<br>Sham+control vs. TBI+control: p=0.0095<br>Sham+control vs. TBI+antagomir: p=0.7307<br>Sham+antagomir vs. TBI+control: p=0.0017<br>Sham+antagomir vs. TBI+antagomir: p=0.2249<br>TBI+control vs. TBI+antagomir: p=0.0584
8I:<br>Fas ligand	Sham+control=4<br>Sham+antagomir=4<br>TBI+control=4<br>TBI+antagomir=4	Normal distribution, homogeneity variances	One-way ANOVA, Tukey multiple comparisons test	F (3, 12) = 2.171	One-way ANOVA, p=0.1445<br><br>Tukey's multiple comparisons test:<br><br>Sham+control vs. Sham+antagomir: p=0.9981<br>Sham+control vs. TBI+control: p=0.1551<br>Sham+control vs. TBI+antagomir: p=0.8095<br>Sham+antagomir vs. TBI+control: p=0.2024<br>Sham+antagomir vs. TBI+antagomir: p=0.8876<br>TBI+control vs. TBI+antagomir: p=0.5236
8I:<br>Fractalkine	Sham+control=4<br>Sham+antagomir=4<br>TBI+control=4<br>TBI+antagomir=4	Normal distribution, homogeneity variances	One-way ANOVA, Tukey multiple	F (3, 12) = 5.097	One-way ANOVA, p=0.0167<br><br>Tukey's multiple comparisons test:

Figure	n	Data structure	Test used	Statistic	P value
			comparisons test		Sham+control vs. Sham+antagomir: p=0.9972<br>Sham+control vs. TBI+control: p=0.5078<br>Sham+control vs. TBI+antagomir: p=0.1225<br>Sham+antagomir vs. TBI+control: p=0.4036<br>Sham+antagomir vs. TBI+antagomir: p=0.1673<br>TBI+control vs. TBI+antagomir: p=0.0105
8I:<br>G-CSF	Sham+control=4<br>Sham+antagomir=4<br>TBI+control=4<br>TBI+antagomir=4	Normal distribution, homogeneity variances	One-way ANOVA, Tukey multiple comparisons test	F (3, 12) = 13.15	One-way ANOVA, p=0.0004<br><br>Tukey's multiple comparisons test:<br><br>Sham+control vs. Sham+antagomir: p=0.7322<br>Sham+control vs. TBI+control: p=0.0021<br>Sham+control vs. TBI+antagomir: p=0.9413<br>Sham+antagomir vs. TBI+control: p=0.0004<br>Sham+antagomir vs. TBI+antagomir: p=0.4151<br>TBI+control vs. TBI+antagomir: p=0.0055
8I:<br>GM-CSF	Sham+control=4<br>Sham+antagomir=4<br>TBI+control=4<br>TBI+antagomir=4	Normal distribution, homogeneity variances	One-way ANOVA, Tukey multiple comparisons test	F (3, 12) = 22.50	One-way ANOVA, p<0.0001<br><br>Tukey's multiple comparisons test:<br><br>Sham+control vs. Sham+antagomir: p=0.7734<br>Sham+control vs. TBI+control: p=0.0004<br>Sham+control vs. TBI+antagomir: p=0.6526<br>Sham+antagomir vs. TBI+control: p<0.0001<br>Sham+antagomir vs. TBI+antagomir: p=0.9963<br>TBI+control vs. TBI+antagomir: p<0.0001
8I:<br>IFN-γ	Sham+control=4<br>Sham+antagomir=4<br>TBI+control=4<br>TBI+antagomir=4	Normal distribution, homogeneity variances	One-way ANOVA, Tukey multiple comparisons test	F (3, 12) = 15.99	One-way ANOVA, p=0.0002<br><br>Tukey's multiple comparisons test:<br><br>Sham+control vs. Sham+antagomir: p=0.7499<br>Sham+control vs. TBI+control: p=0.0008<br>Sham+control vs. TBI+antagomir: p=0.8415<br>Sham+antagomir vs. TBI+control: p=0.0002<br>Sham+antagomir vs. TBI+antagomir: p=0.3071<br>TBI+control vs. TBI+antagomir: p=0.0031
8I:<br>IL1-α	Sham+control=4<br>Sham+antagomir=4<br>TBI+control=4<br>TBI+antagomir=4	Normal distribution, homogeneity variances	One-way ANOVA, Tukey multiple comparisons test	F (3, 12) = 9.266	One-way ANOVA, p=0.0019<br><br>Tukey's multiple comparisons test:<br><br>Sham+control vs. Sham+antagomir: p=0.9394<br>Sham+control vs. TBI+control: p=0.0054<br>Sham+control vs. TBI+antagomir: p=0.7395<br>Sham+antagomir vs. TBI+control: p=0.002<br>Sham+antagomir vs. TBI+antagomir: p=0.4184<br>TBI+control vs. TBI+antagomir: p=0.032
8J:<br>IL1-β	Sham+control=4<br>Sham+antagomir=4<br>TBI+control=4<br>TBI+antagomir=4	Normal distribution, homogeneity variances	One-way ANOVA, Tukey multiple comparisons test	F (3, 12) = 28.52	One-way ANOVA, p<0.0001<br><br>Tukey's multiple comparisons test:<br><br>Sham+control vs. Sham+antagomir: p=0.5874<br>Sham+control vs. TBI+control: p<0.0001<br>Sham+control vs. TBI+antagomir: p=0.412<br>Sham+antagomir vs. TBI+control: p<0.0001<br>Sham+antagomir vs. TBI+antagomir: p=0.057<br>TBI+control vs. TBI+antagomir: p=0.0005
8J:<br>IL2	Sham+control=4<br>Sham+antagomir=4<br>TBI+control=4<br>TBI+antagomir=4	Normal distribution, homogeneity variances	One-way ANOVA, Tukey multiple comparisons test	F (3, 12) = 11.59	One-way ANOVA, p=0.0007<br><br>Tukey's multiple comparisons test:<br><br>Sham+control vs. Sham+antagomir: p=0.9928<br>Sham+control vs. TBI+control: p=0.0027<br>Sham+control vs. TBI+antagomir: p=0.9983<br>Sham+antagomir vs. TBI+control: p=0.0017<br>Sham+antagomir vs. TBI+antagomir: p=0.9996<br>TBI+control vs. TBI+antagomir: p=0.002
8J:<br>IL3	Sham+control=4<br>Sham+antagomir=4<br>TBI+control=4<br>TBI+antagomir=4	Non-normal distribution	Kruskal-Wallis test, Dunn's multiple comparisons	Kruskal-Wallis statistic=10.68	Kruskal-Wallis test, p=0.0023<br><br>Dunn's multiple comparisons test:<br><br>Sham+control vs. Sham+antagomir: p>0.9999

Figure	n	Data structure	Test used	Statistic	P value
			test		Sham+control vs. TBI+control: p=0.1049<br>Sham+control vs. TBI+antagomir: p>0.9999<br>Sham+antagomir vs. TBI+control: p=0.0109<br>Sham+antagomir vs. TBI+antagomir: p=0.8249<br>TBI+control vs. TBI+antagomir: p=0.6139
8J:<br>IL4	Sham+control=4<br>Sham+antagomir=4<br>TBI+control=4<br>TBI+antagomir=4	Normal distribution, homogeneity variances	One-way ANOVA, Tukey multiple comparisons test	F (3, 12) = 2.707	One-way ANOVA, p=0920<br><br>Tukey's multiple comparisons test:<br><br>Sham+control vs. Sham+antagomir: p=0.9326<br>Sham+control vs. TBI+control: p=0.1187<br>Sham+control vs. TBI+antagomir: p=0.23<br>Sham+antagomir vs. TBI+control: p=0.2928<br>Sham+antagomir vs. TBI+antagomir: p=0.5<br>TBI+control vs. TBI+antagomir: p=0.9733
8J:<br>IL6	Sham+control=4<br>Sham+antagomir=4<br>TBI+control=4<br>TBI+antagomir=4	Normal distribution, homogeneity variances	One-way ANOVA, Tukey multiple comparisons test	F (3, 12) = 17.61	One-way ANOVA, p=0.0001<br><br>Tukey's multiple comparisons test:<br><br>Sham+control vs. Sham+antagomir: p=0.9401<br>Sham+control vs. TBI+control: p=0.0004<br>Sham+control vs. TBI+antagomir: p=0.8561<br>Sham+antagomir vs. TBI+control: p=0.0002<br>Sham+antagomir vs. TBI+antagomir: p=0.5453<br>TBI+control vs. TBI+antagomir: p=0.0013
8J:<br>IL9	Sham+control=4<br>Sham+antagomir=4<br>TBI+control=4<br>TBI+antagomir=4	Non-normal distribution	Kruskal-Wallis test, Dunn's multiple comparisons test	Kruskal-Wallis statistic=7.16	Kruskal-Wallis test, p=0.0524<br><br>Dunn's multiple comparisons test:<br><br>Sham+control vs. Sham+antagomir: p>0.9999<br>Sham+control vs. TBI+control: p=0.6139<br>Sham+control vs. TBI+antagomir: p>0.9999<br>Sham+antagomir vs. TBI+control: p=0.1876<br>Sham+antagomir vs. TBI+antagomir: p>0.9999<br>TBI+control vs. TBI+antagomir: p=0.0856
8J:<br>IL10	Sham+control=4<br>Sham+antagomir=4<br>TBI+control=4<br>TBI+antagomir=4	Normal distribution, homogeneity variances	One-way ANOVA, Tukey multiple comparisons test	F (3, 12) = 35.79	One-way ANOVA, p<0.0001<br><br>Tukey's multiple comparisons test:<br><br>Sham+control vs. Sham+antagomir: p=0.9211<br>Sham+control vs. TBI+control: p<0.0001<br>Sham+control vs. TBI+antagomir: p=0.0901<br>Sham+antagomir vs. TBI+control: p<0.0001<br>Sham+antagomir vs. TBI+antagomir: p=0.0306<br>TBI+control vs. TBI+antagomir: p=0.0003
8J:<br>IL12-p40/p70	Sham+control=4<br>Sham+antagomir=4<br>TBI+control=4<br>TBI+antagomir=4	Normal distribution, homogeneity variances	One-way ANOVA, Tukey multiple comparisons test	F (3, 12) = 14.27	One-way ANOVA, p=0.0003<br><br>Tukey's multiple comparisons test:<br><br>Sham+control vs. Sham+antagomir: p=0.9993<br>Sham+control vs. TBI+control: p=0.0006<br>Sham+control vs. TBI+antagomir: p=0.3692<br>Sham+antagomir vs. TBI+control: p=0.0005<br>Sham+antagomir vs. TBI+antagomir: p=0.314<br>TBI+control vs. TBI+antagomir: p=0.0096
8J:<br>IL12-p70	Sham+control=4<br>Sham+antagomir=4<br>TBI+control=4<br>TBI+antagomir=4	Normal distribution, homogeneity variances	One-way ANOVA, Tukey multiple comparisons test	F (3, 12) = 6.351	One-way ANOVA, p=0.0080<br><br>Tukey's multiple comparisons test:<br><br>Sham+control vs. Sham+antagomir: p=0.9666<br>Sham+control vs. TBI+control: p=0.0213<br>Sham+control vs. TBI+antagomir: p=0.3682<br>Sham+antagomir vs. TBI+control: p=0.0096<br>Sham+antagomir vs. TBI+antagomir: p=0.192<br>TBI+control vs. TBI+antagomir: p=0.336
8J:<br>IL13	Sham+control=4<br>Sham+antagomir=4<br>TBI+control=4<br>TBI+antagomir=4	Normal distribution, homogeneity variances	One-way ANOVA, Tukey multiple comparisons test	F (3, 12) = 20.07	One-way ANOVA, p<0.0001<br><br>Tukey's multiple comparisons test:<br><br>Sham+control vs. Sham+antagomir: p=0.836<br>Sham+control vs. TBI+control: p=0.0004

Figure	n	Data structure	Test used	Statistic	P value
					Sham+control vs. TBI+antagomir: p=0.9441<br>Sham+antagomir vs. TBI+control: p=0.0001<br>Sham+antagomir vs. TBI+antagomir: p=0.9915<br>TBI+control vs. TBI+antagomir: p=0.0002
8K:<br>IL17	Sham+control=4<br>Sham+antagomir=4<br>TBI+control=4<br>TBI+antagomir=4	Normal distribution, homogeneity variances	One-way ANOVA, Tukey multiple comparisons test	F (3, 12) = 28.66	One-way ANOVA, p<0.0001<br><br>Tukey's multiple comparisons test:<br><br>Sham+control vs. Sham+antagomir: p=0.9913<br>Sham+control vs. TBI+control: p<0.0001<br>Sham+control vs. TBI+antagomir: p=0.841<br>Sham+antagomir vs. TBI+control: p<0.0001<br>Sham+antagomir vs. TBI+antagomir: p=0.6884<br>TBI+control vs. TBI+antagomir: p<0.0001
8K:<br>I-TAC	Sham+control=4<br>Sham+antagomir=4<br>TBI+control=4<br>TBI+antagomir=4	Normal distribution, homogeneity variances	One-way ANOVA, Tukey multiple comparisons test	F (3, 12) = 20.76	One-way ANOVA, p<0.0001<br><br>Tukey's multiple comparisons test:<br><br>Sham+control vs. Sham+antagomir: p=0.9995<br>Sham+control vs. TBI+control: p=0.0001<br>Sham+control vs. TBI+antagomir: p=0.4452<br>Sham+antagomir vs. TBI+control: p<0.0001<br>Sham+antagomir vs. TBI+antagomir: p=0.3887<br>TBI+control vs. TBI+antagomir: p=0.0011
8K:<br>KC	Sham+control=4<br>Sham+antagomir=4<br>TBI+control=4<br>TBI+antagomir=4	Normal distribution, homogeneity variances	One-way ANOVA, Tukey multiple comparisons test	F (3, 12) = 16.38	One-way ANOVA, p=0.0002<br><br>Tukey's multiple comparisons test:<br><br>Sham+control vs. Sham+antagomir: p=0.9998<br>Sham+control vs. TBI+control: p=0.0006<br>Sham+control vs. TBI+antagomir: p=0.9698<br>Sham+antagomir vs. TBI+control: p=0.0006<br>Sham+antagomir vs. TBI+antagomir: p=0.9828<br>TBI+control vs. TBI+antagomir: p=0.0003
8K:<br>Leptin	Sham+control=4<br>Sham+antagomir=4<br>TBI+control=4<br>TBI+antagomir=4	Normal distribution, homogeneity variances	One-way ANOVA, Tukey multiple comparisons test	F (3, 12) = 27.79	One-way ANOVA, p<0.0001<br><br>Tukey's multiple comparisons test:<br><br>Sham+control vs. Sham+antagomir: p=0.9976<br>Sham+control vs. TBI+control: p<0.0001<br>Sham+control vs. TBI+antagomir: p=0.2706<br>Sham+antagomir vs. TBI+control: p<0.0001<br>Sham+antagomir vs. TBI+antagomir: p=0.206<br>TBI+control vs. TBI+antagomir: p=0.0004
8K:<br>LIX	Sham+control=4<br>Sham+antagomir=4<br>TBI+control=4<br>TBI+antagomir=4	Non-normal distribution	Kruskal-Wallis test, Dunn's multiple comparisons test	Kruskal-Wallis statistic=8.49	Kruskal-Wallis test, p=0.0208<br><br>Dunn's multiple comparisons test:<br><br>Sham+control vs. Sham+antagomir: p>0.9999<br>Sham+control vs. TBI+control: p=0.036<br>Sham+control vs. TBI+antagomir: p=0.9495<br>Sham+antagomir vs. TBI+control: p=0.1876<br>Sham+antagomir vs. TBI+antagomir: p>0.9999<br>TBI+control vs. TBI+antagomir: p>0.9999
8K:<br>Lymphotactin	Sham+control=4<br>Sham+antagomir=4<br>TBI+control=4<br>TBI+antagomir=4	Normal distribution, homogeneity variances	One-way ANOVA, Tukey multiple comparisons test	F (3, 12) = 7.922	One-way ANOVA, p=0.0035<br><br>Tukey's multiple comparisons test:<br><br>Sham+control vs. Sham+antagomir: p=0.9685<br>Sham+control vs. TBI+control: p=0.0126<br>Sham+control vs. TBI+antagomir: p=0.9985<br>Sham+antagomir vs. TBI+control: p=0.0058<br>Sham+antagomir vs. TBI+antagomir: p=0.9911<br>TBI+control vs. TBI+antagomir: p=0.0095
8K:<br>MCP-1	Sham+control=4<br>Sham+antagomir=4<br>TBI+control=4<br>TBI+antagomir=4	Normal distribution, homogeneity variances	One-way ANOVA, Tukey multiple comparisons test	F (3, 12) = 6.168	One-way ANOVA, p=0.0088<br><br>Tukey's multiple comparisons test:<br><br>Sham+control vs. Sham+antagomir: p=0.9994<br>Sham+control vs. TBI+control: p=0.0184<br>Sham+control vs. TBI+antagomir: p=0.9952

Figure	n	Data structure	Test used	Statistic	P value
					Sham+antagomir vs. TBI+control: p=0.0151<br>Sham+antagomir vs. TBI+antagomir: p=0.9848<br>TBI+control vs. TBI+antagomir: p=0.0278
8K:<br>M-CSF	Sham+control=4<br>Sham+antagomir=4<br>TBI+control=4<br>TBI+antagomir=4	Normal distribution, homogeneity variances	One-way ANOVA, Tukey multiple comparisons test	F (3, 12) = 7.621	One-way ANOVA, p=0.0041<br><br>Tukey's multiple comparisons test:<br><br>Sham+control vs. Sham+antagomir: p=0.9452<br>Sham+control vs. TBI+control: p=0.0043<br>Sham+control vs. TBI+antagomir: p=0.2954<br>Sham+antagomir vs. TBI+control: p=0.011<br>Sham+antagomir vs. TBI+antagomir: p=0.5747<br>TBI+control vs. TBI+antagomir: p=0.1062
8K:<br>MIG	Sham+control=4<br>Sham+antagomir=4<br>TBI+control=4<br>TBI+antagomir=4	Normal distribution, homogeneity variances	One-way ANOVA, Tukey multiple comparisons test	F (3, 12) = 10.10	One-way ANOVA, p=0.0013<br><br>Tukey's multiple comparisons test:<br><br>Sham+control vs. Sham+antagomir: p=0.9668<br>Sham+control vs. TBI+control: p=0.0046<br>Sham+control vs. TBI+antagomir: p=0.106<br>Sham+antagomir vs. TBI+control: p=0.0021<br>Sham+antagomir vs. TBI+antagomir: p=0.0488<br>TBI+control vs. TBI+antagomir: p=0.3145
8K:<br>MIP-1-α	Sham+control=4<br>Sham+antagomir=4<br>TBI+control=4<br>TBI+antagomir=4	Normal distribution, homogeneity variances	One-way ANOVA, Tukey multiple comparisons test	F (3, 12) = 6.304	One-way ANOVA, p=0.0082<br><br>Tukey's multiple comparisons test:<br><br>Sham+control vs. Sham+antagomir: p>0.9999<br>Sham+control vs. TBI+control: p=0.0133<br>Sham+control vs. TBI+antagomir: p=0.9103<br>Sham+antagomir vs. TBI+control: p=0.0139<br>Sham+antagomir vs. TBI+antagomir: p=0.9182<br>TBI+control vs. TBI+antagomir: p=0.0423
8L:<br>MIP-1-γ	Sham+control=4<br>Sham+antagomir=4<br>TBI+control=4<br>TBI+antagomir=4	Normal distribution, homogeneity variances	One-way ANOVA, Tukey multiple comparisons test	F (3, 12) = 24.41	One-way ANOVA, p<0.0001<br><br>Tukey's multiple comparisons test:<br><br>Sham+control vs. Sham+antagomir: p>0.9999<br>Sham+control vs. TBI+control: p<0.0001<br>Sham+control vs. TBI+antagomir: p=0.1868<br>Sham+antagomir vs. TBI+control: p=<0.0001<br>Sham+antagomir vs. TBI+antagomir: p=0.1768<br>TBI+control vs. TBI+antagomir: p=0.0011
8L:<br>RANTES	Sham+control=4<br>Sham+antagomir=4<br>TBI+control=4<br>TBI+antagomir=4	Normal distribution, homogeneity variances	One-way ANOVA, Tukey multiple comparisons test	F (3, 12) = 8.126	One-way ANOVA, p=0.0032<br><br>Tukey's multiple comparisons test:<br><br>Sham+control vs. Sham+antagomir: p=0.9989<br>Sham+control vs. TBI+control: p=0.0058<br>Sham+control vs. TBI+antagomir: p=0.5823<br>Sham+antagomir vs. TBI+control: p=0.0046<br>Sham+antagomir vs. TBI+antagomir: p=0.5011<br>TBI+control vs. TBI+antagomir: p=0.0557
8L:<br>SDF-1	Sham+control=4<br>Sham+antagomir=4<br>TBI+control=4<br>TBI+antagomir=4	Normal distribution, homogeneity variances	One-way ANOVA, Tukey multiple comparisons test	F (3, 12) = 13.50	One-way ANOVA, p=0.0004<br><br>Tukey's multiple comparisons test:<br><br>Sham+control vs. Sham+antagomir: p=0.5955<br>Sham+control vs. TBI+control: p=0.0041<br>Sham+control vs. TBI+antagomir: p=0.8661<br>Sham+antagomir vs. TBI+control: p=0.0005<br>Sham+antagomir vs. TBI+antagomir: p=0.9569<br>TBI+control vs. TBI+antagomir: p=0.0011
8L:<br>TCA-3	Sham+control=4<br>Sham+antagomir=4<br>TBI+control=4<br>TBI+antagomir=4	Normal distribution, homogeneity variances	One-way ANOVA, Tukey multiple comparisons test	F (3, 12) = 6.399	One-way ANOVA, p=0.0078<br><br>Tukey's multiple comparisons test:<br><br>Sham+control vs. Sham+antagomir: p=0.9466<br>Sham+control vs. TBI+control: p=0.0078<br>Sham+control vs. TBI+antagomir: p=0.5369<br>Sham+antagomir vs. TBI+control: p=0.0201

Figure	n	Data structure	Test used	Statistic	P value
					Sham+antagomir vs. TBI+antagomir: p=0.8386<br>TBI+control vs. TBI+antagomir: p=0.0849
8L:<br>TECK	Sham+control=4<br>Sham+antagomir=4<br>TBI+control=4<br>TBI+antagomir=4	Normal distribution, homogeneity variances	One-way ANOVA, Tukey multiple comparisons test	F (3, 12) = 15.43	One-way ANOVA, p=0.0002<br><br>Tukey's multiple comparisons test:<br><br>Sham+control vs. Sham+antagomir: p=0.9534<br>Sham+control vs. TBI+control: p=0.0003<br>Sham+control vs. TBI+antagomir: p=0.7021<br>Sham+antagomir vs. TBI+control: p=0.0007<br>Sham+antagomir vs. TBI+antagomir: p=0.9383<br>TBI+control vs. TBI+antagomir: p=0.0017
8L:<br>TIMP1	Sham+control=4<br>Sham+antagomir=4<br>TBI+control=4<br>TBI+antagomir=4	Normal distribution, homogeneity variances	One-way ANOVA, Tukey multiple comparisons test	F (3, 12) = 23.57	One-way ANOVA, p<0.0001<br><br>Tukey's multiple comparisons test:<br><br>Sham+control vs. Sham+antagomir: p=0.9998<br>Sham+control vs. TBI+control: p<0.0001<br>Sham+control vs. TBI+antagomir: p=0.9155<br>Sham+antagomir vs. TBI+control: p<0.0001<br>Sham+antagomir vs. TBI+antagomir: p=0.8835<br>TBI+control vs. TBI+antagomir: p=0.0002
8L:<br>TIMP2	Sham+control=4<br>Sham+antagomir=4<br>TBI+control=4<br>TBI+antagomir=4	Non-normal distribution	Kruskal-Wallis test, Dunn's multiple comparisons test	Kruskal-Wallis statistic=10.85	Kruskal-Wallis test, p=0.0019<br><br>Dunn's multiple comparisons test:<br><br>Sham+control vs. Sham+antagomir: p>0.9999<br>Sham+control vs. TBI+control: p=0.3211<br>Sham+control vs. TBI+antagomir: p>0.9999<br>Sham+antagomir vs. TBI+control: p=0.3211<br>Sham+antagomir vs. TBI+antagomir: p>0.9999<br>TBI+control vs. TBI+antagomir: p=0.0065
8L:<br>TNF-α	Sham+control=4<br>Sham+antagomir=4<br>TBI+control=4<br>TBI+antagomir=4	Normal distribution, homogeneity variances	One-way ANOVA, Tukey multiple comparisons test	F (3, 12) = 9.789	One-way ANOVA, p=0.0015<br><br>Tukey's multiple comparisons test:<br><br>Sham+control vs. Sham+antagomir: p=0.9976<br>Sham+control vs. TBI+control: p=0.0072<br>Sham+control vs. TBI+antagomir: p=0.8028<br>Sham+antagomir vs. TBI+control: p=0.0099<br>Sham+antagomir vs. TBI+antagomir: p=0.7022<br>TBI+control vs. TBI+antagomir: p=0.0016
8L:<br>sTNF RI	Sham+control=4<br>Sham+antagomir=4<br>TBI+control=4<br>TBI+antagomir=4	Normal distribution, homogeneity variances	One-way ANOVA, Tukey multiple comparisons test	F (3, 12) = 19.83	One-way ANOVA, p<0.0001<br><br>Tukey's multiple comparisons test:<br><br>Sham+control vs. Sham+antagomir: p=0.9985<br>Sham+control vs. TBI+control: p=0.0001<br>Sham+control vs. TBI+antagomir: p=0.1092<br>Sham+antagomir vs. TBI+control: p=0.0001<br>Sham+antagomir vs. TBI+antagomir: p=0.0837<br>TBI+control vs. TBI+antagomir: p=0.0072
8L:<br>sTNF RII	Sham+control=4<br>Sham+antagomir=4<br>TBI+control=4<br>TBI+antagomir=4	Normal distribution, homogeneity variances	One-way ANOVA, Tukey multiple comparisons test	F (3, 12) = 10.70	One-way ANOVA, p=0.0010<br><br>Tukey's multiple comparisons test:<br><br>Sham+control vs. Sham+antagomir: p=0.9982<br>Sham+control vs. TBI+control: p=0.0047<br>Sham+control vs. TBI+antagomir: p=0.9218<br>Sham+antagomir vs. TBI+control: p=0.0035<br>Sham+antagomir vs. TBI+antagomir: p=0.967<br>TBI+control vs. TBI+antagomir: p=0.0016
S2B	Sham+WT=6<br>Sham+KO=6<br>TBI+WT=6<br>TBI+KO=6	Normal distribution, homogeneity variances	One-way ANOVA, Tukey multiple comparisons test	F (3, 20) = 76.42	One-way ANOVA, p<0.0001<br><br>Tukey's multiple comparisons test:<br><br>Sham+WT vs. Sham+KO: p=0.6719<br>Sham+WT vs. TBI+WT: p<0.0001<br>Sham+WT vs. TBI+KO: p=0.0726<br>Sham+KO vs. TBI+WT: p<0.0001<br>Sham+KO vs. TBI+KO: p=0.044

Figure	n	Data structure	Test used	Statistic	P value
					TBI+WT vs. TBI+KO: p<0.0001
S2C	Sham+WT=6<br>Sham+KO=6<br>TBI+WT=6<br>TBI+KO=6	Normal distribution, homogeneity variances	One-way ANOVA, Tukey multiple comparisons test	F (3, 20) = 62.14	One-way ANOVA, p<0.0001<br><br>Tukey's multiple comparisons test:<br><br>Sham+WT vs. Sham+KO: p=0.9921<br>Sham+WT vs. TBI+WT: p<0.0001<br>Sham+WT vs. TBI+KO: p=0.832<br>Sham+KO vs. TBI+WT: p<0.0001<br>Sham+KO vs. TBI+KO: p=0.6787<br>TBI+WT vs. TBI+KO: p<0.0001
S2D	Sham+WT=6<br>Sham+KO=6<br>TBI+WT=6<br>TBI+KO=6	Normal distribution, homogeneity variances	One-way ANOVA, Tukey multiple comparisons test	F (3, 20) = 17.85	One-way ANOVA, p<0.0001<br><br>Tukey's multiple comparisons test:<br><br>Sham+WT vs. Sham+KO: p=0.995<br>Sham+WT vs. TBI+WT: p<0.0001<br>Sham+WT vs. TBI+KO: p=0.9363<br>Sham+KO vs. TBI+WT: p<0.0001<br>Sham+KO vs. TBI+KO: p=0.8434<br>TBI+WT vs. TBI+KO: p=0.0001
S3B	Sham+WT=6<br>Sham+KO=6<br>TBI+WT=6<br>TBI+KO=6	Normal distribution, homogeneity variances	One-way ANOVA, Tukey multiple comparisons test	F (3, 20) = 70.79	One-way ANOVA, p<0.0001<br><br>Tukey's multiple comparisons test:<br><br>Sham+WT vs. Sham+KO: p>0.9999<br>Sham+WT vs. TBI+WT: p<0.0001<br>Sham+WT vs. TBI+KO: p=0.007<br>Sham+KO vs. TBI+WT: p<0.0001<br>Sham+KO vs. TBI+KO: p=0.007<br>TBI+WT vs. TBI+KO: p<0.0001
S3C	Sham+WT=6<br>Sham+KO=6<br>TBI+WT=6<br>TBI+KO=6	Normal distribution, homogeneity variances	One-way ANOVA, Tukey multiple comparisons test	F (3, 20) = 35.98	One-way ANOVA, p<0.0001<br><br>Tukey's multiple comparisons test:<br><br>Sham+WT vs. Sham+KO: p=0.88<br>Sham+WT vs. TBI+WT: p<0.0001<br>Sham+WT vs. TBI+KO: p=0.9633<br>Sham+KO vs. TBI+WT: p<0.0001<br>Sham+KO vs. TBI+KO: p=0.6241<br>TBI+WT vs. TBI+KO: p<0.0001
S3D	Sham+WT=6<br>Sham+KO=6<br>TBI+WT=6<br>TBI+KO=6	Normal distribution, homogeneity variances	One-way ANOVA, Tukey multiple comparisons test	F (3, 20) = 73.53	One-way ANOVA, p<0.0001<br><br>Tukey's multiple comparisons test:<br><br>Sham+WT vs. Sham+KO: p>0.9999<br>Sham+WT vs. TBI+WT: p<0.0001<br>Sham+WT vs. TBI+KO: p=0.0068<br>Sham+KO vs. TBI+WT: p<0.0001<br>Sham+KO vs. TBI+KO: p=0.0059<br>TBI+WT vs. TBI+KO: p<0.0001
S3G	Sham+WT=6<br>Sham+KO=6<br>TBI+WT=6<br>TBI+KO=6	Normal distribution, homogeneity variances	One-way ANOVA, Tukey multiple comparisons test	F (3, 20) = 191.5	One-way ANOVA, p<0.0001<br><br>Tukey's multiple comparisons test:<br><br>Sham+WT vs. Sham+KO: p=0.9997<br>Sham+WT vs. TBI+WT: p<0.0001<br>Sham+WT vs. TBI+KO: p<0.0001<br>Sham+KO vs. TBI+WT: p<0.0001<br>Sham+KO vs. TBI+KO: p<0.0001<br>TBI+WT vs. TBI+KO: p<0.0001
S3H	Sham+WT=6<br>Sham+KO=6<br>TBI+WT=6<br>TBI+KO=6	Normal distribution, homogeneity variances	One-way ANOVA, Tukey multiple comparisons test	Kruskal-Wallis statistic=4.362	Kruskal-Wallis test, p=0.22249<br><br>Dunn's multiple comparisons test:<br><br>Sham+WT vs. Sham+KO: p>0.9999<br>Sham+WT vs. TBI+WT: p=0.2523<br>Sham+WT vs. TBI+KO: p>0.9999<br>Sham+KO vs. TBI+WT: p=0.9166<br>Sham+KO vs. TBI+KO: p>0.9999<br>TBI+WT vs. TBI+KO: p>0.9999
S4B	Pre=3	Normal	One-way	F (5, 12) = 21.18	One-way ANOVA, p<0.0001

Figure	n	Data structure	Test used	Statistic	P value
	1d=3<br>2d=3<br>3d=3<br>5d=3<br>7d=3	distribution,<br>homogeneity variances	ANOVA, Dunnett's multiple comparisons test		Dunnett's multiple comparisons test<br><br>Pre vs. 1d: p<0.0001<br>Pre vs. 2d: p=0.0004<br>Pre vs. 3d: p<0.0001<br>Pre vs. 5d: p<0.0001<br>Pre vs. 7d: p=0.0002
S4C	Pre=3<br>1d=3<br>2d=3<br>3d=3<br>5d=3<br>7d=3	Normal distribution, homogeneity variances	One-way ANOVA, Dunnett's multiple comparisons test	F (5, 12) = 16.39	One-way ANOVA, p<0.0001<br><br>Dunnett's multiple comparisons test<br><br>Pre vs. 1d: p=0.0016<br>Pre vs. 2d: p=0.0111<br>Pre vs. 3d: p<0.0001<br>Pre vs. 5d: p<0.0001<br>Pre vs. 7d: p=0.0003
S4D	Pre=3<br>1d=3<br>2d=3<br>3d=3<br>5d=3<br>7d=3	Normal distribution, homogeneity variances	One-way ANOVA, Dunnett's multiple comparisons test	F (5, 12) = 14.86	One-way ANOVA, p<0.0001<br><br>Dunnett's multiple comparisons test<br><br>Pre vs. 1d: p<0.0001<br>Pre vs. 2d: p<0.0001<br>Pre vs. 3d: p=0.01<br>Pre vs. 5d: p=0.0104<br>Pre vs. 7d: p=0.0008
S4E	Pre=3<br>1d=3<br>2d=3<br>3d=3<br>5d=3<br>7d=3	Normal distribution, homogeneity variances	One-way ANOVA, Dunnett's multiple comparisons test	F (5, 12) = 13.22	One-way ANOVA, p<0.0001<br><br>Dunnett's multiple comparisons test<br><br>Pre vs. 1d: p<0.0001<br>Pre vs. 2d: p=0.0023<br>Pre vs. 3d: p=0.0009<br>Pre vs. 5d: p=0.1475<br>Pre vs. 7d: p=0.1495
S6A	TBI+control=10<br>TBI+antagomir=10	Non-normal/Normal distribution, homogeneity variances	Unpaired t test or Mann Whitney test	t=2.590, df=18<br>U=35<br>t=1.055, df=18<br>t=1.063, df=18<br>t=1.689, df=18<br>t=2.541, df=18	TBI+control vs. TBI+antagomir Slice 1: p=0.0185<br>TBI+control vs. TBI+antagomir Slice 2: p=0.2799<br>TBI+control vs. TBI+antagomir Slice 3: p=0.3054<br>TBI+control vs. TBI+antagomir Slice 4: p=0.3021<br>TBI+control vs. TBI+antagomir Slice 5: p=0.1085<br>TBI+control vs. TBI+antagomir Slice 6: p=0.0205
S6D	Sham+control=6<br>Sham+antagomir=6<br>TBI+control=6<br>TBI+antagomir=6	Normal distribution, homogeneity variances	One-way ANOVA, Tukey multiple comparisons test	F (3, 20) = 133.3	One-way ANOVA, p<0.0001<br><br>Tukey's multiple comparisons test:<br><br>Sham+control vs. Sham+antagomir: p>0.9999<br>Sham+control vs. TBI+control: p<0.0001<br>Sham+control vs. TBI+antagomir: p<0.0001<br>Sham+antagomir vs. TBI+control: p<0.0001<br>Sham+antagomir vs. TBI+antagomir: p<0.0001<br>TBI+control vs. TBI+antagomir: p<0.0001
S6E	Sham+control=6<br>Sham+antagomir=6<br>TBI+control=6<br>TBI+antagomir=6	Normal distribution, homogeneity variances	One-way ANOVA, Tukey multiple comparisons test	F (3, 20) = 129.1	One-way ANOVA, p<0.0001<br><br>Tukey's multiple comparisons test:<br><br>Sham+control vs. Sham+antagomir: p=0.8004<br>Sham+control vs. TBI+control: p<0.0001<br>Sham+control vs. TBI+antagomir: p<0.0001<br>Sham+antagomir vs. TBI+control: p<0.0001<br>Sham+antagomir vs. TBI+antagomir: p<0.0001<br>TBI+control vs. TBI+antagomir: p<0.0001
S6F	Sham+control=6<br>Sham+antagomir=6<br>TBI+control=6<br>TBI+antagomir=6	Normal distribution, homogeneity variances	One-way ANOVA, Tukey multiple comparisons test	F (3, 20) =0.6869	One-way ANOVA, p= 0.5705<br><br>Tukey's multiple comparisons test:<br><br>Sham+control vs. Sham+antagomir: p=0.7562<br>Sham+control vs. TBI+control: p=0.9948<br>Sham+control vs. TBI+antagomir: p=0.6189<br>Sham+antagomir vs. TBI+control: p=0.8762<br>Sham+antagomir vs. TBI+antagomir: p=0.9953<br>TBI+control vs. TBI+antagomir: p=0.7608
S7B	Sham+control=6<br>Sham+antagomir=6<br>TBI+control=6<br>TBI+antagomir=6	Normal distribution, homogeneity variances	One-way ANOVA, Tukey multiple comparisons test	F (3, 20) = 144.1	One-way ANOVA, p<0.0001<br><br>Tukey's multiple comparisons test:<br><br>Sham+control vs. Sham+antagomir: p=0.9979<br>Sham+control vs. TBI+control: p<0.0001

Figure	n	Data structure	Test used	Statistic	P value
					Sham+control vs. TBI+antagomir: p<0.0001<br>Sham+antagomir vs. TBI+control: p<0.0001<br>Sham+antagomir vs. TBI+antagomir: p<0.0001<br>TBI+control vs. TBI+antagomir: p<0.0001
S7C	Sham+control=6<br>Sham+antagomir=6<br>TBI+control=6<br>TBI+antagomir=6	Normal distribution, homogeneity variances	One-way ANOVA, Tukey multiple comparisons test	F (3, 20) = 13.48	One-way ANOVA, p<0.0001<br><br>Tukey's multiple comparisons test:<br><br>Sham+control vs. Sham+antagomir: p>0.9999<br>Sham+control vs. TBI+control: p=0.0001<br>Sham+control vs. TBI+antagomir: p=0.622<br>Sham+antagomir vs. TBI+control: p=0.0001<br>Sham+antagomir vs. TBI+antagomir: p=0.6399<br>TBI+control vs. TBI+antagomir: p=0.002
S7D	Sham+control=6<br>Sham+antagomir=6<br>TBI+control=6<br>TBI+antagomir=6	Normal distribution, homogeneity variances	One-way ANOVA, Tukey multiple comparisons test	F (3, 20) = 67.78	One-way ANOVA, p<0.0001<br><br>Tukey's multiple comparisons test:<br><br>Sham+control vs. Sham+antagomir: p=0.9965<br>Sham+control vs. TBI+control: p<0.0001<br>Sham+control vs. TBI+antagomir: p=0.2106<br>Sham+antagomir vs. TBI+control: p<0.0001<br>Sham+antagomir vs. TBI+antagomir: p=0.2945<br>TBI+control vs. TBI+antagomir: p<0.0001

Supplementary information

Loss-of-microRNA-15a/16-1 function promotes neuropathological and functional recovery in experimental traumatic brain injury

Chao Zhou[1,2]; Shun Li[1,2]; Na Qiu[1,2]; Ping Sun[1,2]; Milton H. Hamblin[3]; C. Edward Dixon[2,4]; Jun Chen[1,2]; Ke-Jie Yin[1,2]*.

1. Department of Neurology, University of Pittsburgh School of Medicine, Pittsburgh, PA 15213, USA

2. Geriatric Research Education and Clinical Center, Veterans Affairs Pittsburgh Healthcare System, Pittsburgh, PA 15240, USA

3. Division of Biomedical Sciences, School of Medicine, University of California Riverside, Riverside, CA 92521, USA

4. Department of Neurosurgery, University of Pittsburgh School of Medicine, Pittsburgh, PA, 15213, USA

***Correspondence addressed to:** Ke-Jie Yin, M.D., Ph.D.

Department of Neurology, University of Pittsburgh School of Medicine, S514 BST, 200 Lothrop Street, Pittsburgh, PA 15213

Email: yink2@upmc.edu

Tel: 412-383-6038

Fax: 412-648-8081

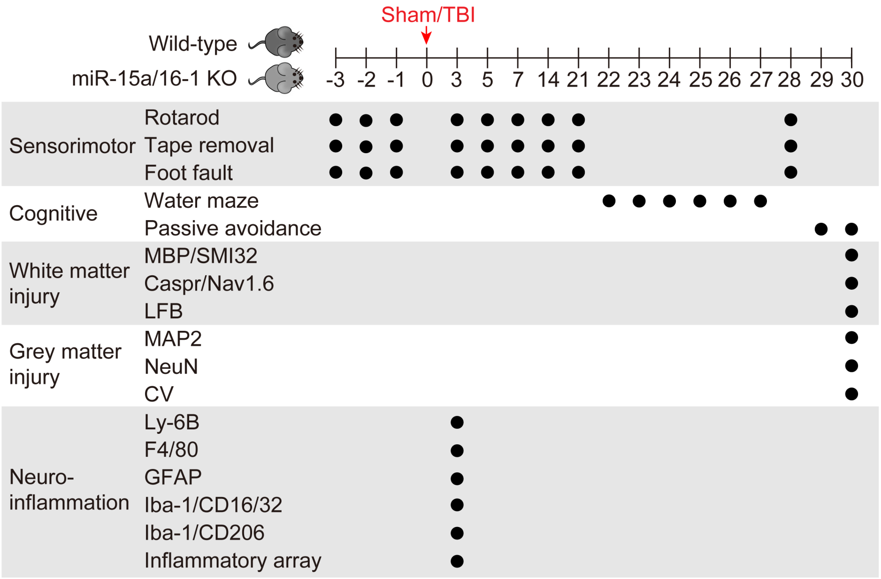

Figure S1 Schematic diagram of experimental design. MiR-15a/16-1 KO and WT mice were subjected to TBI or sham operation. Long-term sensorimotor function was examined by rotarod test, adhesive tape removal test, and foot fault test in experimental mice before and up to 28 d after operation. Cognitive function was examined by Morris water maze test and passive avoidance test in mice 23-30 d after operation. White matter injury was evaluated by histological staining of LFB, double immunostaining of MBP/SMI32, and Caspr/Nav1.6 in mouse brain sections at 30 d after operation. Grey matter injury was evaluated by histological staining of CV, and immunostaining of MAP2 and NeuN. Neuroinflammation was examined by Ly-6B, F4/80, GFAP, Iba-1/CD16/32, Iba-1/CD206 immunostaining, and inflammatory array.

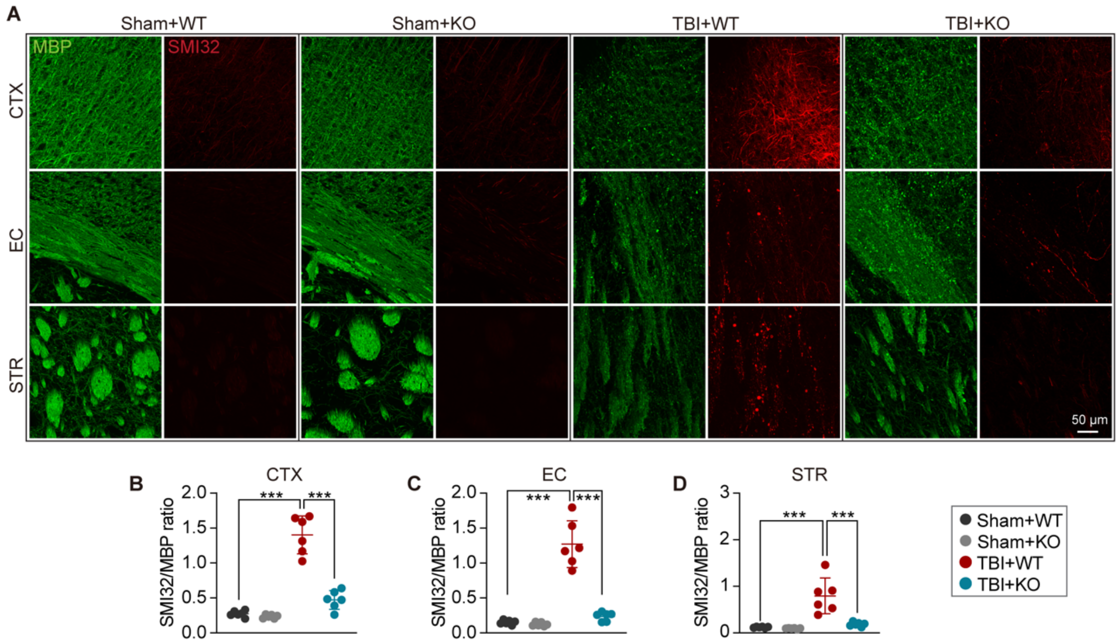

Figure S2 Genetic deletion of the miR-15a/16-1 cluster reduces axonal damage in mouse brains at 30 d after TBI. (A) Representative immunofluorescence staining images of MBP (green) and SMI32 (red) in peri-lesional cerebral cortex (CTX), external capsule (EC), and striatum (STR) areas. (B-D). Quantitative analysis of the SMI32/MBP ratio in peri-lesional CTX, EC, and STR areas showed less axonal damage in miR-15a/16-1 KO mice 30 d after TBI compared with WT controls. Data are represented as mean ± SD, n=6/group. *$p<0.05$, **$p<0.01$, or ***$p<0.001$ versus TBI+WT group. Statistical analyses were performed by one-way ANOVA and Tukey's tests. Related to Figure 3

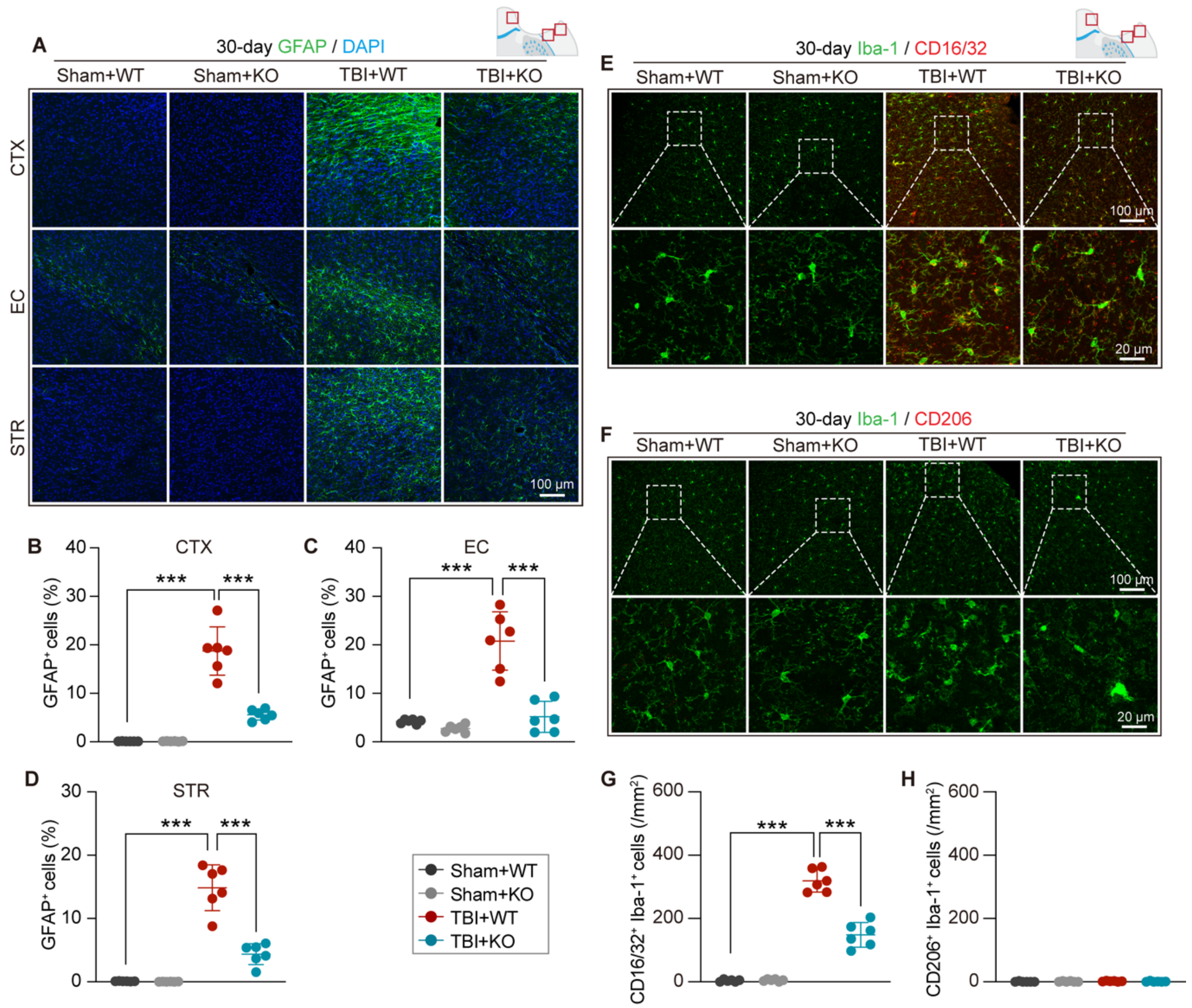

Figure S3 Genetic deletion of miR-15a/16-1 reduced astrocytic activation and the polarization of pro-inflammatory microglia/macrophages 30 d after TBI. (A) Representative immunofluorescence staining images of GFAP (green) / DAPI (blue) in peri-lesional cerebral cortex (CTX), external capsule (EC), and striatum (STR) areas. (B-D) Quantitative analysis of GFAP-positive cells in peri-lesional CTX, EC, and STR areas. (E, F) Representative immunofluorescence staining images of Iba-1 (green) / CD16/32 (red) and Iba-1 (green) / CD206 (red). (G, H) Quantitative analysis of Iba-1&CD16/32-positive cells and Iba-1&CD206-positive cells in peri-lesional regions. Data are represented as mean ± SD, n=6/group. *p<0.05, **p<0.01, or ***p<0.001 versus TBI+WT group. Statistical analyses were performed by one-way ANOVA and Tukey's tests.

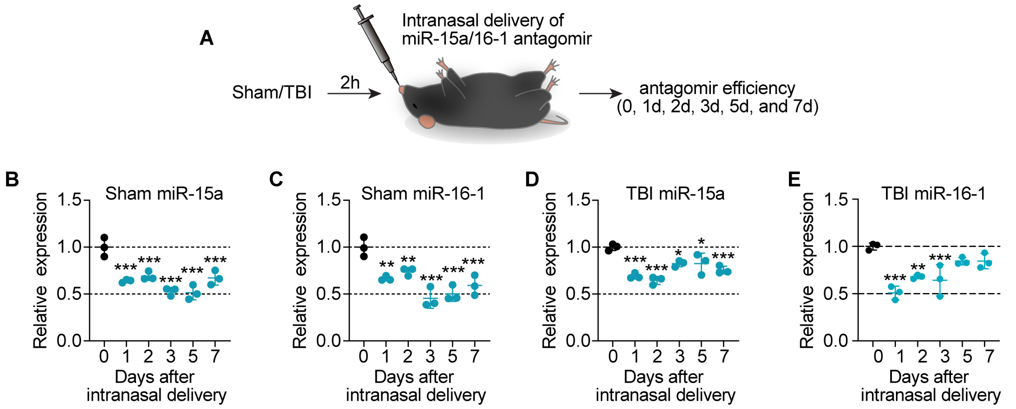

Figure S4 Intranasal delivery of the miR-15a/16-1 antagomir reduces cerebral miR-15a/16-1 expression under sham or TBI conditions. (A) A total of 1 nmol (8.1μg)/24 μl miR-15a/16-1 antagomir was intranasally delivered to C57BL/6J mice 2 h after TBI/sham operation and the miR-15a/16-1 inhibitory efficiency was examined in mouse brains up to 7 d after administration. Quantitative PCR was used to detect the relative expression of miR-15a and miR-16-1 in mouse brains at pre, 1, 2, 3, 5, and 7 d after intranasal delivery of the miR-15a/16-1 antagomir. (B, C) Quantitative analysis of the relative expression of miR-15a and miR-16-1 in mouse brains under sham conditions. (D, E) Quantitative analysis of the relative expression of miR-15a and miR-16-1 in mouse brains under TBI conditions.

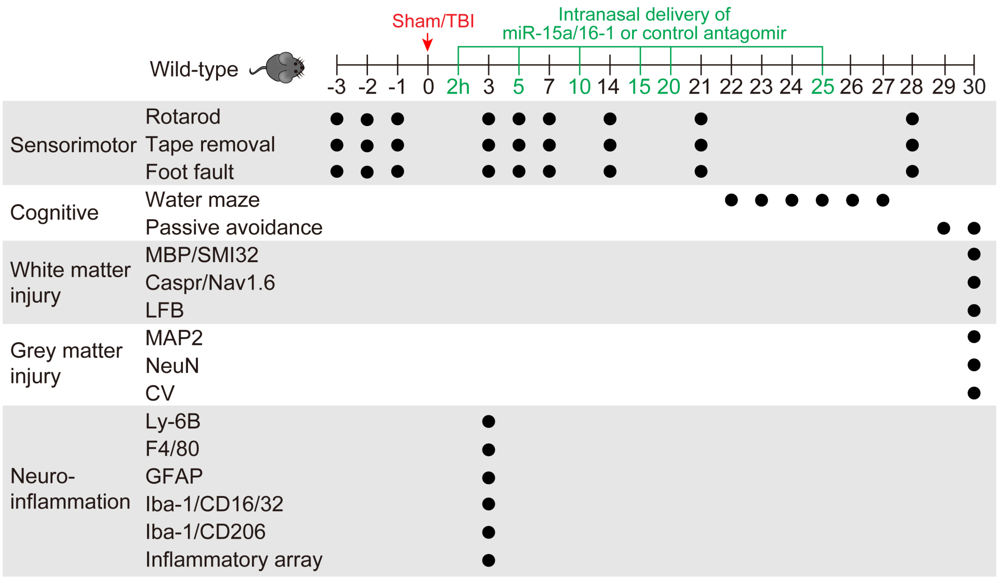

Figure S5 Schematic diagram of experimental design. C57BL/6J mice were subjected to TBI or sham operation. MiR-15a/16-1 antagomir or control antagomir was intranasally delivered to the mice at 2 h, 5 d, 10 d, 15 d, 20 d, and 25 d after CCI surgery. Long-term sensorimotor function was examined by rotarod test, adhesive tape removal test, and foot fault test in experimental mice before and up to 28 d after operation. Cognitive function was examined by the Morris water maze test and passive avoidance test in mice 23-30 d after operation. White matter injury was evaluated by histological staining of LFB, double immunostaining of MBP/SMI32, and Caspr/Nav1.6 in mouse brain sections at 30 d after operation. Grey matter injury was evaluated by histological staining of CV, and immunostaining of MAP2 and NeuN. Neuroinflammation was examined by Ly-6B, F4/80, GFAP, Iba-1/CD16/32, Iba-1/CD206 immunostaining, and inflammatory array.

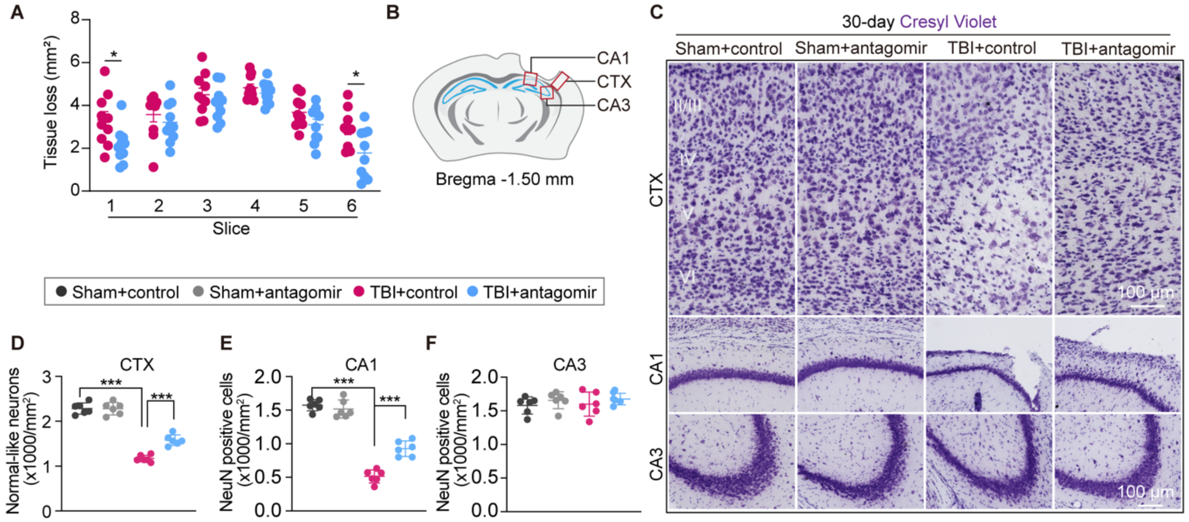

Figure S6 Intranasal delivery of miR-15a/16-1 antagomir reduces TBI-induced neuronal loss. C57BL/6J mice were subjected to experimental TBI and intranasally treated with miR-15a/16-1 antagomir or control antagomir at 2 h, 5 d, 10 d, 15 d, 20 d, and 25 d after CCI surgery. MAP2 immunostaining and CV staining were performed to examine brain tissue loss and neuronal loss in the peri-lesional cerebral cortex (CTX), and hippocampal CA1 and CA3 areas at 30 d after TBI. (A) Quantitative analysis of brain tissue loss in each cross-sectional area immunostained by MAP2. (B) Coordinates and brain regions for CV staining. (C) Representative images of CV staining in peri-lesional CTX, CA1, and CA3 regions. (D-F) Quantitative analysis of CV-stained cells in peri-lesional CTX, CA1, and CA3 regions.

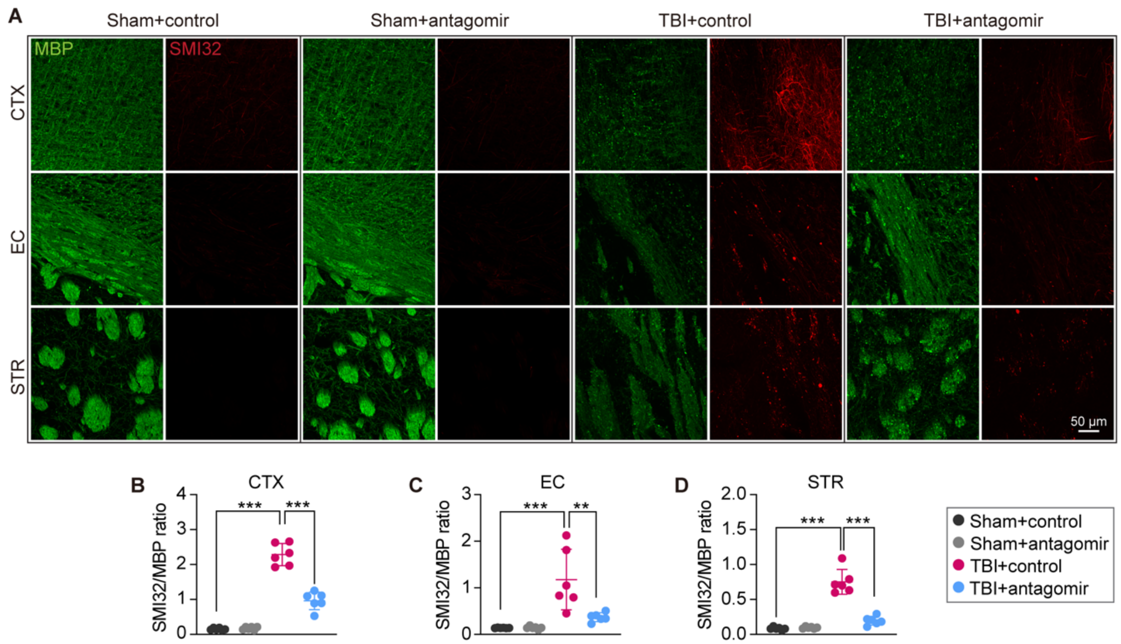

Figure S7 Intranasal delivery of the miR-15a/16-1 antagomir reduces axonal damage in mouse brains at 30 d after TBI. (A) Representative immunofluorescence staining images of MBP (green) and SMI32 (red) in the peri-lesional cerebral cortex (CTX), external capsule (EC), and striatum (STR) areas. (B-D). Quantitative analysis of the SMI32/MBP ratio in peri-lesional CTX, EC, and STR areas showed less axonal damage in miR-15a/16-1 antagomir-treated mice 30 d after TBI compared with control antagomir-treated mice. Data are represented as mean ± SD, n=6/group. *p<0.05, **p<0.01, or ***p<0.001 versus TBI+control antagomir group. Statistical analyses were performed by one-way ANOVA and Tukey's tests. Related to Figure 6.

	A	B	C	D	E	F	G	H	I	J	K	L
1–2	POS	POS	NEG	NEG	Blank	BLC	CD30L	Eotaxin	Eotaxin-2	FAS ligand	Fractal--kine	G-CSF
3–4	GM-CSF	IFN-γ	IL1-α	IL1-β	IL2	IL3	IL4	IL6	IL9	IL10	IL12-p40/p70	IL12-p70
5–6	IL13	IL17	I-TAC	KC	Leptin	LIX	Lympho--tactin	MCP-1	M-CSF	MIG	MIP-1-α	MIP-1-γ
7–8	RANTES	SDF-1	TCA-3	TECK	TIMP-1	TIMP-2	TNF-α	sTNF RI	sTNF RII	Blank	Blank	POS

Figure S8 Array map of 40 inflammatory mediators. Related to Figure 8.

Table S1. List of primary antibodies used in this study

Antibody	Host species	Dilution	Company	Cat #
MAP2	Rabbit	1:500	Millipore	AB5622
NeuN	Rabbit	1:500	EMD Millipore	ABN78
MBP	Rabbit	1:500	Abcam	Ab40390
SMI32	Mouse	1:500	BioLegend	801701
Caspr	Mouse	1:200	EMD Millipore	MABN69
Nav1.6	Rabbit	1:200	Millipore	AB5580-50UL
GFAP	Rabbit	1:500	Sigma-Aldrich	G9269
Iba-1	Rabbit	1:500	Wako	019-19741
CD16/32	Mouse	1:200	BD Biosciences	553124
CD206	Goat	1:200	R&D Systems	AF2535
Ly-6B	Rat	1:100	Abcam	Ab53453
F4/80	Mouse	1:200	BioLegend	123102

Table S2. Pearson correlation analysis (r value) in Figure 2

	Latency to fall	Time to touch	Time to remove	Forepaw foot fault	Hindpaw foot fault	Latency to platform	Target time	Latency to dark
CV-CTX	0.86706	-0.79914	-0.85740	-0.80001	-0.82078	-0.70096	0.47817	0.58136
CV-CA1	0.89831	-0.92213	-0.87595	-0.91672	-0.87386	-0.85406	0.62714	0.71896
CV-CA3	0.16612	-0.06261	-0.21223	-0.18359	-0.20059	-0.04923	0.15444	0.38573
NeuN-CTX	0.87773	-0.88180	-0.89025	-0.90132	-0.88108	-0.81867	0.60380	0.73205
NeuN-CA1	0.90530	-0.80652	-0.82693	-0.78134	-0.81959	-0.63697	0.63905	0.73602
NeuN-CA3	0.31180	-0.34527	-0.52110	-0.32519	-0.34287	-0.27249	-0.05297	0.31715

Table S3. Pearson correlation analysis (p value) in Figure 2

	Latency to fall	Time to touch	Time to remove	Forepaw foot fault	Hindpaw foot fault	Latency to platform	Target time	Latency to dark
CV-CTX	4.22E-08	2.82E-06	8.70E-08	2.71E-06	8.98E-07	0.00013603	0.01810675	0.00288747
CV-CA1	2.57E-09	1.53E-10	2.05E-08	3.12E-10	2.45E-08	1.10E-07	0.00103854	7.55E-05
CV-CA3	0.43787916	0.77131998	0.31944405	0.39050289	0.34731557	0.81928733	0.47118979	0.06266417
NeuN-CTX	1.77E-08	1.24E-08	5.72E-09	1.88E-09	1.32E-08	1.01E-06	0.00178284	4.78E-05
NeuN-CA1	1.22E-09	1.94E-06	6.30E-07	6.57E-06	9.60E-07	0.00081651	0.00077513	4.14E-05
NeuN-CA3	0.13801319	0.09844227	0.00902517	0.12101023	0.10096218	0.19768005	0.8058364	0.1310258

Table S4. Pearson correlation analysis (r value) in Figure 3

	Latency to fall	Time to touch	Time to remove	Forepaw foot fault	Hindpaw foot fault	Latency to platform	Target time	Latency to dark
LFB-CTX	0.863232	-0.86484	-0.85019	-0.87763	-0.84367	-0.76888	0.592332	0.666777
LFB-EC	0.899337	-0.86566	-0.80047	-0.87721	-0.86472	-0.79596	0.643032	0.820506
LFB-STR	0.865641	-0.85245	-0.83466	-0.86384	-0.91838	-0.80759	0.647759	0.654122
MBP-CTX	0.850161	-0.90457	-0.86175	-0.83624	-0.88277	-0.79752	0.653146	0.587781
MBP-EC	0.897282	-0.9075	-0.84445	-0.82299	-0.87717	-0.74958	0.636873	0.687698
MBP-STR	0.858782	-0.80766	-0.77746	-0.81569	-0.81134	-0.74908	0.588445	0.628437
SMI32-CTX	-0.85907	0.778746	0.785814	0.905284	0.866624	0.849342	-0.69787	-0.669
SMI32-EC	-0.82218	0.821646	0.738757	0.884964	0.89308	0.887689	-0.74307	-0.69979
SMI32-STR	-0.79243	0.846477	0.73011	0.730578	0.833625	0.691158	-0.64264	-0.6924
SMI32/MBP ratio-CTX	-0.88523	0.836236	0.841945	0.914872	0.903435	0.864767	-0.70525	-0.6644
SMI32/MBP ratio-EC	-0.84391	0.863386	0.757162	0.873439	0.904244	0.874154	-0.73936	-0.71122
SMI32/MBP ratio-STR	-0.72316	0.792744	0.685113	0.683667	0.781045	0.68314	-0.60691	-0.58597
Number of NOR	0.904024	-0.93031	-0.87743	-0.88967	-0.89668	-0.84476	0.65075	0.665149
Paranode length	0.84846	-0.90487	-0.88875	-0.89932	-0.88921	-0.87335	0.621393	0.696302

Length of paranode gap	-0.61963	0.635958	0.585451	0.523172	0.57521	0.407121	-0.48093	-0.60414

Table S5. Pearson correlation analysis (p value) in Figure 3

	Latency to fall	Time to touch	Time to remove	Forepaw foot fault	Hindpaw foot fault	Latency to platform	Target time	Latency to dark
LFB-CTX	5.65E-08	5.00E-08	1.45E-07	1.78E-08	2.24E-07	1.14E-05	0.002291	0.000373
LFB-EC	2.31E-09	4.70E-08	2.64E-06	1.85E-08	5.05E-08	3.30E-06	0.000701	9.12E-07
LFB-STR	4.71E-08	1.24E-07	3.96E-07	5.40E-08	2.52E-10	1.84E-06	0.000621	0.000526
MBP-CTX	1.45E-07	1.32E-09	6.32E-08	3.59E-07	1.14E-08	3.06E-06	0.00054	0.002524
MBP-EC	2.86E-09	9.48E-10	2.13E-07	7.92E-07	1.85E-08	2.49E-05	0.000818	0.000204
MBP-STR	7.87E-08	1.83E-06	7.82E-06	1.19E-06	1.51E-06	2.53E-05	0.002489	0.001007
SMI32-CTX	7.70E-08	7.39E-06	5.36E-06	1.22E-09	4.36E-08	1.53E-07	0.00015	0.000351
SMI32-EC	8.29E-07	8.55E-07	3.74E-05	9.36E-09	4.35E-09	7.29E-09	3.19E-05	0.000141
SMI32-STR	3.92E-06	1.86E-07	5.12E-05	5.04E-05	4.22E-07	0.000184	0.000708	0.000177
SMI32/MBP ratio-CTX	9.14E-09	3.59E-07	2.50E-07	3.94E-10	1.49E-09	5.03E-08	0.000119	0.000399
SMI32/MBP ratio-EC	2.20E-07	5.59E-08	1.84E-05	2.53E-08	1.37E-09	2.39E-08	3.66E-05	9.77E-05
SMI32/MBP ratio-STR	6.54E-05	3.86E-06	0.000221	0.00023	6.66E-06	0.000234	0.001663	0.002623
Number of NOR	1.40E-09	4.69E-11	1.81E-08	6.05E-09	3.04E-09	2.08E-07	0.000575	0.000391
Paranode length	1.63E-07	1.27E-09	6.60E-09	2.32E-09	6.32E-09	2.55E-08	0.001191	0.000157
Length of paranode gap	0.001241	0.000837	0.002651	0.008707	0.003276	0.048326	0.017358	0.001769

Table S6. Pearson correlation analysis (r value) in Figure 5

	Latency to fall	Time to touch	Time to remove	Forepaw foot fault	Hindpaw foot fault	Latency to platform	Target time	Latency to dark
CV-CTX	0.90772	-0.88519	-0.91923	-0.95170	-0.93755	-0.78547	0.77350	0.76386
CV-CA1	0.80187	-0.83018	-0.84218	-0.86008	-0.91102	-0.68542	0.61121	0.62419
CV-CA3	0.42378	-0.25336	-0.29438	-0.39200	-0.33771	-0.40436	0.32606	0.50319

NeuN-CTX	0.89929	-0.91071	-0.93377	-0.93444	-0.95229	-0.78147	0.74774	0.70779
NeuN-CA1	0.90834	-0.89306	-0.91729	-0.95125	-0.94676	-0.77581	0.73029	0.74079
NeuN-CA3	0.09714	0.09747	0.08542	-0.00440	-0.08921	-0.08876	-0.05075	0.09244

Table S7. Pearson correlation analysis (p value) in Figure 5

	Latency to fall	Time to touch	Time to remove	Forepaw foot fault	Hindpaw foot fault	Latency to platform	Target time	Latency to dark
CV-CTX	9.24E-10	9.17E-09	2.26E-10	9.20E-13	1.45E-11	5.44E-06	9.31E-06	1.40E-05
CV-CA1	2.46E-06	5.20E-07	2.46E-07	7.15E-08	6.29E-10	0.00021884	0.00150879	0.00111471
CV-CA3	0.0390509	0.23225277	0.16261237	0.05815806	0.10654572	0.05001383	0.11996526	0.01219456
NeuN-CTX	2.32E-09	6.53E-10	2.72E-11	2.44E-11	8.04E-13	6.53E-06	2.67E-05	0.00010934
NeuN-CA1	8.61E-10	4.36E-09	2.90E-10	1.02E-12	2.62E-12	8.41E-06	5.09E-05	3.47E-05
NeuN-CA3	0.65158947	0.65049415	0.69145967	0.98371735	0.6784971	0.68001553	0.81383077	0.66749596

Table S8. Pearson correlation analysis (r value) in Figure 6

	Latency to fall	Time to touch	Time to remove	Forepaw foot fault	Hindpaw foot fault	Latency to platform	Target time	Latency to dark
LFB-CTX	0.898301	-0.87988	-0.92187	-0.93185	-0.92679	-0.7834	0.717602	0.745231
LFB-EC	0.925555	-0.92439	-0.94334	-0.95204	-0.95139	-0.79168	0.738511	0.774838
LFB-STR	0.915822	-0.89372	-0.91218	-0.93322	-0.91161	-0.79213	0.688876	0.780024
MBP-CTX	0.868032	-0.89052	-0.91431	-0.90433	-0.8987	-0.76754	0.671694	0.661861
MBP-EC	0.9075	-0.92144	-0.93302	-0.95453	-0.93058	-0.82173	0.737293	0.765639
MBP-STR	0.86401	-0.85606	-0.89456	-0.86867	-0.85964	-0.81266	0.690281	0.758786
SMI32-CTX	-0.91689	0.861622	0.89266	0.932901	0.917276	0.73467	-0.77713	-0.73852
SMI32-EC	-0.67055	0.803759	0.758704	0.699539	0.69381	0.53942	-0.54828	-0.59039
SMI32-STR	-0.88636	0.930092	0.932197	0.878643	0.853788	0.864338	-0.79471	-0.75361
SMI32/MBP ratio-CTX	-0.92546	0.908066	0.956562	0.940657	0.916054	0.853767	-0.82715	-0.82617
SMI32/MBP ratio-EC	-0.71712	0.821077	0.787198	0.738916	0.723701	0.604065	-0.57165	-0.67405
SMI32/MBP ratio-STR	-0.85875	0.897988	0.910239	0.858246	0.836226	0.853579	-0.79572	-0.75751
Number of NOR	0.882345	-0.90854	-0.92541	-0.93198	-0.93061	-0.7586	0.771114	0.696569
Paranode length	0.90686	-0.89759	-0.92943	-0.94166	-0.93256	-0.79654	0.732753	0.8276

Length of paranode gap	-0.52336	0.363205	0.475748	0.545272	0.545995	0.466124	-0.35985	-0.41916

Table S9. Pearson correlation analysis (p value) in Figure 6

	Latency to fall	Time to touch	Time to remove	Forepaw foot fault	Hindpaw foot fault	Latency to platform	Target time	Latency to dark
LFB-CTX	2.57E-09	1.47E-08	1.59E-10	3.70E-11	7.93E-11	5.98E-06	7.90E-05	2.94E-05
LFB-EC	9.48E-11	1.12E-10	5.12E-12	8.51E-13	9.84E-13	4.06E-06	3.78E-05	8.78E-06
LFB-STR	3.50E-10	4.09E-09	5.47E-10	2.97E-11	5.86E-10	3.98E-06	0.000197	6.97E-06
MBP-CTX	3.91E-08	5.58E-09	4.22E-10	1.35E-09	2.47E-09	1.20E-05	0.000325	0.000427
MBP-EC	9.48E-10	1.68E-10	3.07E-11	4.79E-13	4.50E-11	8.51E-07	3.95E-05	1.30E-05
MBP-STR	5.33E-08	9.58E-08	3.76E-09	3.72E-08	7.39E-08	1.40E-06	0.000189	1.73E-05
SMI32-CTX	3.05E-10	6.38E-08	4.54E-09	3.13E-11	2.91E-10	4.35E-05	7.93E-06	3.78E-05
SMI32-EC	0.000336	2.24E-06	1.73E-05	0.000142	0.00017	0.006523	0.005539	0.002388
SMI32-STR	8.25E-09	4.85E-11	3.50E-11	1.64E-08	1.13E-07	5.20E-08	3.51E-06	2.12E-05
SMI32/MBP ratio-CTX	9.61E-11	8.88E-10	2.93E-13	8.41E-12	3.40E-10	1.13E-07	6.22E-07	6.59E-07
SMI32/MBP ratio-EC	8.03E-05	8.83E-07	5.02E-06	3.72E-05	6.42E-05	0.001772	0.00352	0.000304
SMI32/MBP ratio-STR	7.89E-08	2.66E-09	6.90E-10	8.18E-08	3.60E-07	1.14E-07	3.34E-06	1.82E-05
Number of NOR	1.18E-08	8.41E-10	9.67E-11	3.62E-11	4.48E-11	1.74E-05	1.03E-05	0.000156
Paranode length	1.02E-09	2.77E-09	5.36E-11	6.99E-12	3.30E-11	3.21E-06	4.66E-05	6.06E-07
Length of paranode gap	0.008679	0.081074	0.018784	0.005857	0.005779	0.021687	0.084137	0.041465
