# Supplementary figures and images for: Loss of microRNA-15a/16-1 function promotes neuropathological and functional recovery in experimental traumatic brain injury
Source: JCI Insight. 2024 Jun 24;9(12):e178650. doi: 10.1172/jci.insight.178650 (PMC11383186; doi:10.1172/jci.insight.178650)

Full unedited gel for Figure 8

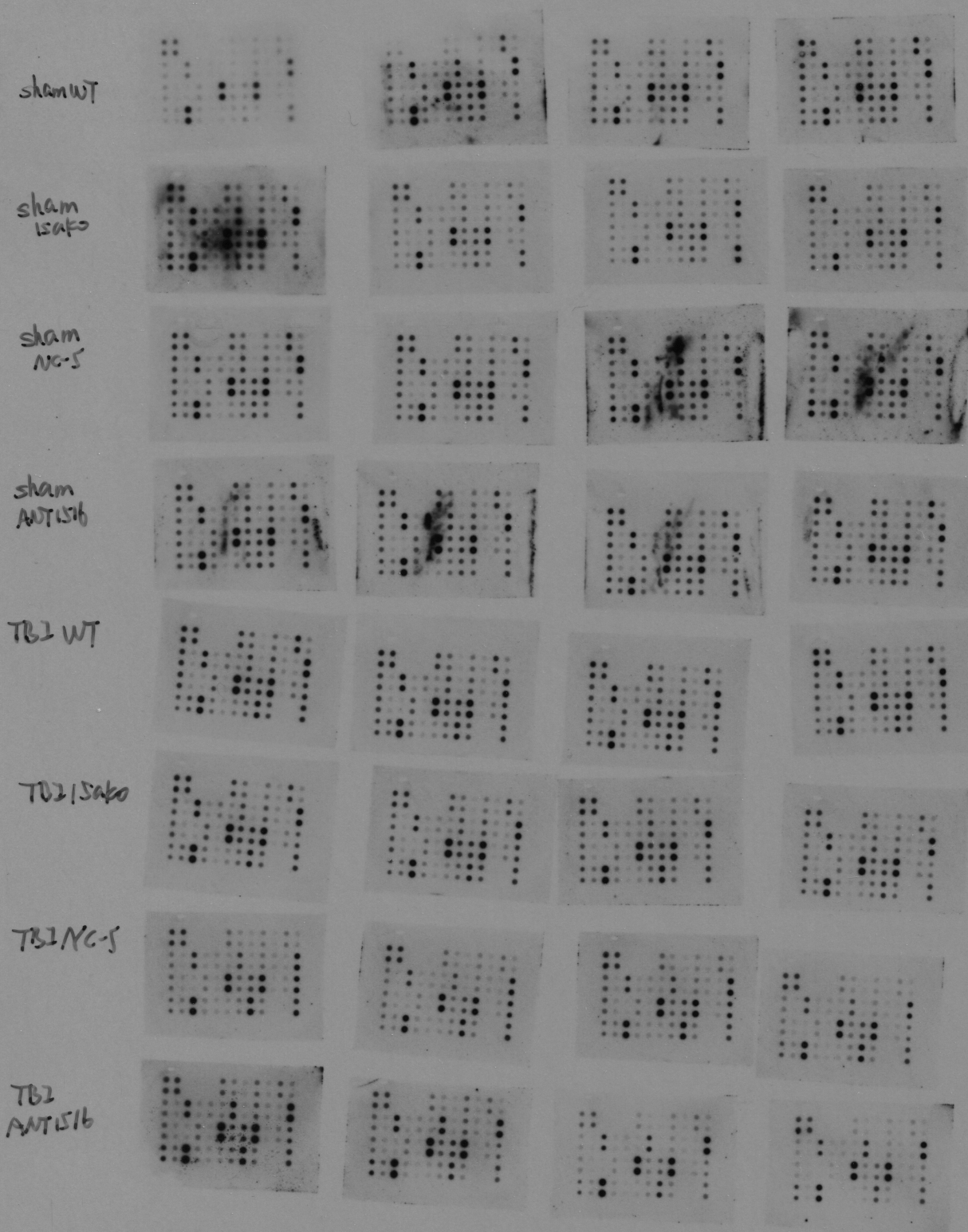

Supplement: Unedited blot and gel images [file jciinsight-9-178650-s213.pdf]
